# The role of *APOE* gene polymorphisms in lung adenocarcinoma susceptibility and lipid profile

**DOI:** 10.3389/fimmu.2024.1522761

**Published:** 2024-12-23

**Authors:** Huanhuan Bi, Dunqiang Ren, Ye Wang, Hongmei Wang, Chunling Zhang

**Affiliations:** ^1^ Department of Respiratory and Critical Care Medicine, School of Medicine, Qingdao University, Qingdao, China; ^2^ Department of Respiratory and Critical Care Medicine, The Affiliated Hospital of Qingdao University, Qingdao, Shandong, China; ^3^ Clinical Laboratory, Qingdao Central Hospital, University of Health and Rehabilitation Sciences, Qingdao, Shandong, China; ^4^ Department of Respiratory and Critical Care Medicine, Qingdao Central Hospital, University of Health and Rehabilitation Sciences, Qingdao, Shandong, China

**Keywords:** APOE, gene polymorphisms, lung adenocarcinoma, lipid metabolism, total cholesterol

## Abstract

**Background:**

APOE gene polym orphisms have been linked to Alzheimer’s disease and coronary heart diseases. However, their relationship with lung adenocarcinoma (LUAD) remains uncertain.

**Methods:**

This study analyzed a cohort of 600 individuals comprising 200 LUAD patients in the lung cancer group and 400 healthy individuals as controls. APOE gene variants were identified through Sanger sequencing. Statistical analyses were conducted to assess intergroup differences, and comparisons of lipid profiles were performed across individuals carrying different APOE alleles.

**Results:**

The *APOE* ϵ2 allele had been significantly more frequently occurring in the LUAD group than in the control group (15.5% vs. 7%, P <0.001). *APOE* ϵ2/ϵ2 and ϵ2/ϵ3 genotypes increased susceptibility to LUAD by 3.78-fold and 3.22-fold. The APOE ϵ2/ϵ3 genotype increased the risk of early-stage LUAD by 2.36-fold and advanced-stage LUAD by 4.05-fold. Individuals with the *APOE* ϵ2/ϵ2 genotype had a 3.22-fold higher susceptibility to moderately differentiated and a 6.8-fold higher susceptibility to poorly differentiated LUAD. Patients with the ϵ2 allele in LUAD exhibited disrupted lipid metabolism, characterized by reduced HDL, TC, and FFA levels, along with increased ApoB, particularly in advanced and poorly differentiated cancer stages.

**Conclusion:**

Individuals carrying the ϵ2 allele have an increased susceptibility to developing LUAD, accompanied by disrupted lipid metabolism. Additionally, the APOE ϵ2/ϵ2 and ϵ2/ϵ3 genotypes are associated with an increased risk of developing advanced and poorly differentiated LUAD.

## 1 Introduction

The apolipoprotein E (APOE) gene, located on chromosome 19q13.32, encodes the 34 KDa glycoprotein known as *APOE*, which is essential for the transportation and metabolism of lipoproteins (1). Differences at two mutation sites (rs429358 and rs7412) in the *APOE* gene give rise to three distinct alleles: ϵ2 (*APOE*2), ϵ3 (*APOE*3), and ϵ4 (*APOE*4) allele. The *APOE*3 is the most common allele, with *APOE* 2 and *APOE*4 are relatively less frequent variants. The alleles account for six genotypes: *APOE* ϵ2/ϵ2, ϵ3/ϵ3, ϵ4/ϵ4, ϵ2/ϵ3,ϵ2/ϵ4 andϵ3/ϵ4) (2).The *APOE* gene polymorphism is closely associated with Alzheimer’s disease (AD), the *APOE* ϵ2 allele is considered the most potent protective factor against AD (3, 4). *APOE* alleles are involved in the development and progression of various malignancies (5, 6), such as hepatocellular carcinoma (7), testicular cancer (8), pancreatic cancer (9), melanoma (10, 11), colorectal cancer (12) and breast cancers (13, 14). However, research on their role in lung adenocarcinoma (LUAD) remains limited.

The varying effects of different *APOE* genotypes on lipid metabolism highlight their potential impact on cardiovascular and cancer risks (15, 16).Studies show that individuals with the *APOE* ϵ2 allele demonstrate diminished receptor-binding activity, which results in lower total cholesterol (TC) levels and higher triglyceride (TG) concentrations. In contrast, *APOE* ϵ4 carriers have higher plasma TC levels compared to those with the common *APOE* ϵ3 carriers. According to a recent meta-analysis, those with the *APOE* ϵ4 allele have a 42% higher risk of developing coronary heart disease (CHD) than people with the common *APOE* ϵ3 allele (2). This association may be due to the altered expression levels of low-density lipoprotein cholesterol (LDL) and high-density lipoprotein (HDL), resulting from the efficient binding of the *APOE* ϵ2 to LDL and HDL receptor particles (17, 18). This observation underscores the potential influence of phenotypic variations in *APOE* on cancer risk through changes in serum lipoprotein levels (19). Nevertheless, the specific role of *APOE* genotypes in LUAD remains unclear.

## 2 Materials and methods

### 2.1 Study population

The Qingdao University Affiliated Hospital’s Institutional Review Boards (IRBs) approved the study. Whole blood sample were selected from our research group’s repository collected from respiratory and critical care medicine inpatients between June 2018 and June 2022 at the hospitals. Serum was extracted from anticoagulated blood samples derived from the health examination department and preserved for further study at -80°C. The study included 400 healthy control participants and 200 LUAD patients. All participants’ clinical and demographic data were recorded, along with their alcohol and tobacco use, fatty liver disease, and lipid profiles. The criteria for hypertension included systolic/diastolic blood pressure readings of 140/90 mmHg or higher or the continuous treatment with antihypertensive medications. CHD is characterized by coronary artery atherosclerosis, leading to luminal stenosis or functional changes in coronary arteries, resulting in symptoms such as myocardial ischemia and hypoxia or the management of coronary heart disease. Diabetes is diagnosed based on fasting plasma glucose levels ≥7.0 mmol/L, a random plasma glucose level ≥11.1 mmol/L, or the administration of antidiabetic therapies. Nonalcoholic fatty liver disease (NAFLD) is defined as the accumulation of liver fat in individuals who consume little to no alcohol and do not have other identifiable causes of hepatic fat accumulation (20).

### 2.2 Participant grouping

Clinical information and histological analysis, including the outcomes of biopsies or surgical resections and TNM staging in accordance with the most recent edition of the American Joint Committee on Cancer (AJCC) guidelines, were adopted to diagnose LUAD (21). The control group participants were selected to match the age range of patients with LUAD, with a deviation of ±2 years. Individuals in the control group had no relevant medical conditions pertinent to this study, no history of tumors in any organ, and no acute or chronic diseases.

### 2.3 DNA extraction and APOE genotyping

Blood samples were treated with the Blood Genomic DNA Extraction Kit (Catalog number DP348-03, Tiangen, China) to extract deoxyribonucleic acid (DNA), a spectrophotometer was used to measure the purity and concentration of the DNA (Thermo Fisher Scientific, United States). Standard PCR reactions employed the 2×Taq PCR Master Mix (dye-free) reagent kit. The primer sequences for *APOE* were the forward primer (5′- GCTTGGCACGGCTGTCCAAGGA-3)and reverse primer (5′- ATTCGCCCCGGCCTGGTACAC -3’). Following PCR amplification, the PCR products were subjected to 2% agarose gel electrophoresis to verify successful amplification. The gel was stained with ethidium bromide, and the bands were visualized under UV light to confirm the presence and expected size of the amplicons. After confirming the correct PCR products by agarose gel electrophoresis. The amplification products were transferred to Sangon Biotech (Shanghai, China) for Sanger sequencing. SnapGene software (Insightful Science, United States) was used to visualize the sequencing data of two mutation sites (rs7412 and rs 429358) to identify the *APOE* genotype.

### 2.4 Statistical analysis

For statistical analysis, we reported the data as percentages and frequency distributions using SPSS version 27.0 (IBM Corp, Armonk, NY, USA).The chi-square test or Fisher’s exact test was employed for comparing the frequency distribution of APOE genotypes and alleles in the LUAD and healthy control groups, Mann-Whitney U test was adopted for continuous or ordinal data that did not satisfy the normality assumption. To assess the relationship between genotypes and the risk of developing LUAD, binary logistic regression analysis was performed. In this analysis, odds ratios (ORs) were calculated to quantify the association between APOE genotypes and the likelihood of developing LUAD. ORs provide an intuitive measure of the strength of the relationship between predictor variables (genotypes) and the outcome (LUAD risk). This is particularly useful for understanding the magnitude of risk associated with different genotypes. For normally distributed continuous data, an independent samples t-test was performed to analyze further group differences in the *APOE* gene subtypes. P-value<0.05 was recognized as predictive of statistical significance.

## 3 Results

### 3.1 Analysis of baseline characteristics in the study groups

There were 600 participants in the whole, divided between both the LUAD group (n = 200) and the control group (n = 400). The baseline characteristics of the two groups, including an average age over 55 years, showed no statistically significant differences in gender, alcohol use, smoking history, hypertension, or diabetes (P > 0.05) (**Table 1**).

**Table 1:** Characteristics of the study population.

Characteristics	Group		*P-value*
	LUAD	Control	
Age, median (IQR)	57.5(23)	58(30)	0.933
Male/female (%)	105/95(52.5%/47.5%)	237/163(59.3%/40.8%)	0.137
Smoking	89/44.5%)	180(45%)	0.931
Drinking (%)	35(17.5%)	98(24.5%)	0.06
Hypertension (%)	63(31.5%)	124(31%)	0.926
Diabetes (%)	24(12%)	38(9.5%)	0.393
Fatty liver (%)	51(25.5%)	95(23.8%)	0.687
TC (mmol/L)	1.68(1.77)	2.67(3.5)	**<0.001**
TG (mmol/L)	2.14(0.96)	1.86(0.93)	**0.024**
LDL (mmol/L)	2.84(1.19)	2.93(1.11)	**0.030**
HDL (mmol/L)	1.71(0.75)	2.33(2.89)	**<0.001**
APOA1 (mmol/L)	1.25(0.92)	1.73(0.69)	**<0.001**
APOB (mmol/L)	1.25(0.92)	1.93(0.4)	**<0.001**
Lp(a)(mmol/L)	177(113.3)	402.5(206)	**<0.001**
FFA (mmol/L)	0.35(0.39)	0.52(0.39)	**<0.001**

IQR, Interquartile Range; LUAD, lung adenocarcinoma; TC, Total Cholesterol; TG, Triglycerides; LDL, Low-Density Lipoprotein; HDL, High-Density Lipoprotein; APOA1, Apolipoprotein A1; APOB, Apolipoprotein B; Lp(a), Lipoprotein (a); FFA, Free Fatty Acids.

Bold P-values indicate statistically significant differences (P < 0.05).

### 3.2 Analysis of APOE alleles and genotypes frequencies in LUAD and control group

The statistical analysis revealed that the LUAD group had a significantly higher frequency of ϵ2 allele expression than the control group (15.5%vs7%, P< 0.001), conversely, the ϵ4 allele’s expression frequency was significantly lower than that of the control group (2.7% vs 11.1%, P < 0.001). The frequency of ϵ3 allele expression did not differ statistically significantly between the LUAD and control groups (P > 0.05) (**Table 2**). The binary logistic regression analysis revealed strong correlations between *APOE* genotypes and the incidence of LUAD. Specifically, *APOE* ϵ2/ϵ2 and ϵ2/ϵ3 genotypes were significantly associated with a 3.78-fold (95% CI: 1.64–8.71, P<0.001) and 3.22-fold (95% CI: 1.69–6.11, P=0.02) increase in the odds of developing LUAD, respectively. Conversely, the *APOE* ϵ3/ϵ4 genotype was linked to a 0.14-fold reduction in the risk of developing LUAD (95% CI: 0.05–0.4, P<0.001). The proportion of individuals of the *APOE* ϵ3/ϵ3 and ϵ2/ϵ4 genotypes was similar between the two groups (P>0.05) (**Table 3**).

**Table 2:** Comparison of *ApoE* alleles and genotypes frequencies in LUAD and control group.

Allele, Genotype	LUAD (N = 200)	Control (N =400)	*p*-Value
ϵ2 allele, n (%)	62(15.5%)	56(7%)	**0.00**
ϵ3 allele, n (%)	327(81.8%)	655(81.9%)	0.96
ϵ4 allele, n (%)	11(2.7%)	89(11.1%)	**0.00**
*ApoE* ϵ2ϵ2	16(8.0%)	9(2.3%)	**0.00**
*ApoE* ϵ2ϵ3	25(12.5%)	17(4.3%)	**0.00**
*ApoE* ϵ3ϵ3	149(74.5%)	294(73.5%)	0.79
*ApoE* ϵ2ϵ4	5(2.5%)	21(5.3%)	0.13
*ApoE* ϵ3ϵ4	4(2.0%)	50(12.5%)	**0.00**
*ApoE* ϵ4ϵ4	1(0.5%)	9(2.3%)	0.15

OR, Odds Ratio; OR, Odds Ratio.

Bold P-values indicate statistically significant differences (P < 0.05).

**Table 3:** Binary logistic regression analysis of *ApoE* genotypes distribution in LUAD and control group.

Genotype	OR	95%CI	*p*-Value
*ApoE* ϵ2ϵ2	3.78	1.64-8.71	**0.00**
*ApoE* ϵ2ϵ3	3.22	1.69-6.11	**0.02**
*ApoE* ϵ3ϵ3	1.05	0.72-1.55	0.79
*ApoE* ϵ2ϵ4	0.46	0.17-1.25	0.12
*ApoE* ϵ3ϵ4	0.14	0.05-0.40	**0.00**
*ApoE* ϵ4ϵ4	0.22	0.03-1.74	0.15

Bold P-values indicate statistically significant differences (P < 0.05).

### 3.3 Analysis of APOE alleles and genotypes frequencies between the control and LUAD subgroups with different stages

LUAD patients were divided into early and late stages in order to investigate the relationship among *APOE* genotypes and individual LUAD stages in comparison to the control group. Both the early-stage and advanced-stage LUAD groups had a significantly higher frequency of the ϵ2 allele compared to the control group (11.6% vs. 7% and 21.2% vs. 7%, P=0.003 and P<0.01, respectively). However, the frequency of the ϵ4 allele was significantly lower in both LUAD stages compared to the control group (3.7% vs. 11.1% and 2.1% vs. 11.1%, P<0.01). The frequency of the ϵ3 allele did not differ statistically significantly between the control group and each of the other LUAD subgroups (P>0.05). Additionally, the percentage of individuals of the *APOE* ϵ2/ϵ3 genotype was higher in both LUAD subgroups as compared to control group, whereas the proportion of the *APOE* ϵ3/ϵ4 genotype was significantly lower (P<0.05) (**Table 4**).

**Table 4:** Comparison of *ApoE* alleles and genotypes frequencies between the control and LUAD different stages subgroups with different stages.

Allele, Genotype	Control (N =400)	Early (N =95)	*p*-Value	Advanced (N=105)	*p*-Value
ϵ2 allele, n (%)	56(7%)	22(11.6%)	0.003	40(21.2%)	**0.003**
ϵ3 allele, n (%)	655(81.9%)	161(84.7%)	0.366	166(87.4%)	0.370
ϵ4 allele, n (%)	89(11.1%)	7(3.7%)	0.003	4(2.1%)	**0.000**
*ApoE* ϵ2ϵ2	9(2.3%)	5(5.3%)	0.110	11(10.5%)	**0.001**
*ApoE* ϵ2ϵ3	17(4.3%)	9(9.5%)	0.040	16(15.2%)	**0.000**
*ApoE* ϵ3ϵ3	294(73.5%)	75(78.9%)	0.297	74(70.5%)	0.535
*ApoE* ϵ2ϵ4	21(5.3%)	3(3.2%)	0.393	2(1.9%)	0.191
*ApoE* ϵ3ϵ4	50(12.5%)	2(2.1%)	0.003	2(1.9%)	**0.002**
*ApoE* ϵ4ϵ4	9(2.3%)	1(1.1%)	0.695	0(0%)	0.215

Bold P-values indicate statistically significant differences (P < 0.05).

Regression analysis was conducted to examine the association between specific *APOE* genotypes and the risk of LUAD at various stages. The *APOE* ϵ2/ϵ3 genotype showed a 2.36-fold increase in susceptibility to early-stage LUAD compared to the control group (P = 0.046). In patients with advanced-stage LUAD, the frequencies of the *APOE* ϵ2/ϵ2 and ϵ2/ϵ3 genotypes were markedly higher (OR = 5.08,95%CI,2.05-12.62, and OR = 4.05, 95%CI, 1.97-8.33, P < 0.001). Furthermore, individuals with the *APOE* ϵ3/ϵ4 genotype *exhibited* a significantly lower risk of developing both early-stage and advanced-stage LUAD (OR = 0.15,95%CI,0.04-0.63, P=0.01 and OR = 0.14, 95%CI, 0.03-0.57, P =0.006) (**Table 5**).

**Table 5:** Binary logistic regression analysis of *ApoE* genotypes in the control group and early and advanced LUAD stage subgroup.

LUAD	Genotype	OR	95%CI	*p*-Value
Early stage	*ApoE* ϵ2ϵ2	2.41	0.79-7.37	0.122
	*ApoE* ϵ2ϵ3	2.36	1.02-5.47	**0.046**
	*ApoE* ϵ3ϵ3	1.35	0.79-2.32	0.274
	*ApoE* ϵ2ϵ4	0.59	0.17-2.02	0.399
	*ApoE* ϵ3ϵ4	0.15	0.04-0.63	**0.010**
	*ApoE* ϵ4ϵ4	0.46	0.06-3.69	0.467
Advanced stage	*ApoE* ϵ2ϵ2	5.08	2.05-12.62	**0.000**
	*ApoE* ϵ2ϵ3	4.05	1.97-8.33	**0.000**
	*ApoE* ϵ3ϵ3	0.86	0.54-1.38	0.535
	*ApoE* ϵ2ϵ4	0.35	0.08-1.52	0.161
	*ApoE* ϵ3ϵ4	0.14	0.03-0.57	**0.006**
	*ApoE* ϵ4ϵ4	–	–	–

Bold P-values indicate statistically significant differences (P < 0.05).

### 3.4 Analysis of the prevalence of APOE alleles and genotypes frequencies among the control group and LUAD subgroups by differentiation level

Following the degree of pathological differentiation, patients with LUAD were categorized into three subgroups: well, moderately, and poorly differentiated. Based on the degree of tumor differentiation, each LUAD subgroup was compared with the control group, revealing that the frequency of ϵ2 and ϵ4 allele carriers was reduced in well-differentiated LUAD patients (0.8% vs. 7% and 4.2% vs. 11.1%, P=0.01 and P=0.02), while the frequency of ϵ3 allele carriers was significantly higher (94.9% vs. 81.9%, P<0.01). The LUAD subgroup and control group a were analyzed, revealing a much higher frequency of individuals carrying the ϵ2 allele in the moderately differentiated LUAD group (79.9% vs. 81.9%, P<0.01). In the poorly differentiated LUAD group, with respect to the control group, the frequency of the ϵ4 allele was lower (2.8% vs. 11.1%, P < 0.01), whereas the frequency of the ϵ2 allele was higher (26.1% vs. 7%, P < 0.01) (**Table 6** **).**

**Table 6:** Comparison of *ApoE* alleles and genotypes frequencies among the control group and LUAD group subgroups by differentiation level.

Allele	Control (N =400)	Well (N = 59)	*p*-Value	moderately (N = 72)	*p*-Value	poorly (N =69)	*p*-Value
ϵ2 allele, n (%)	56(7%)	1 (0.8%)	**0.010**	25(17.4%)	0.000	36(26.1%)	**0.000**
ϵ3 allele, n (%)	655(81.9%)	112 (94.9%)	**0.000**	115(79.9%)	0.566	100(72.5%)	**0.010**
ϵ4 allele, n (%)	89(11.1%)	5(4.2%)	**0.021**	4(2.8%)	0.002	2(1.4%)	**0.001**
*ApoE* ϵ2ϵ2	9 (2.3%)	0 (0.0%)	0.383	7(9.8%)	0.001	9(13.3%)	**0.000**
*ApoE* ϵ2ϵ3	17 (4.3%)	0 (0.0%)	0.146	9(12.5%)	0.010	16(23.2%)	**0.000**
*ApoE* ϵ3ϵ3	294 (73.5%)	55 (93.2%)	**0.001**	52(72.2%)	0.821	42(60.9%)	**0.032**
*ApoE* ϵ2ϵ4	21(5.3%)	1 (1.7%)	0.233	2(2.8%)	0.411	2(2.9%)	0.554
*ApoE* ϵ3ϵ4	50 (12.5%)	2 (3.4%)	**0.045**	2(2.8%)	0.015	0(0)	**0.002**
*ApoE* ϵ4ϵ4	9 (2.3%)	1 (1.7%)	1.000	0(0)	0.367	0(0)	0.368

Bold P-values indicate statistically significant differences (P < 0.05).

Investigating the association between *APOE* genotypes and poorly differentiated LUAD, we discovered that those with the ϵ2/ϵ2 and ϵ2/ϵ3 genotypes had 6.8-fold and 6.52-fold increased chances of getting this condition. Conversely, individuals with the *APOE* ϵ3/ϵ3 genotype had 0.56 times the odds of developing poorly differentiated LUAD (95% CI = 0.33–0.96, P = 0.03). *APOE* ϵ3/ϵ4 and *APOE* ϵ4/ϵ4 hadn’t been found in patients with poorly differentiated LUAD. The *APOE* ϵ2/ϵ3 and ϵ3/ϵ3genotypes raised the likelihood of developing moderately differentiated LUAD by 4.68-fold and 3.22-fold, respectively, in comparison with the healthy cohort (95% CI = 1.68–13.0 and 1.38–7.54, P < 0.05). However, the probability of developing moderately differentiated LUAD were 0.2-fold lower for carriers of the *APOE* ϵ3/ϵ4 genotype (95% CI = 0.05–0.84, P = 0.03). Furthermore, *APOE* ϵ3/ϵ3 genotype carrier showed a substantial 4.96-fold increase in the odds of developing well-differentiated LUAD (95% CI = 1.75-14.01, P = 0.003). Neither the *APOE* ϵ2/ϵ2 nor the ϵ2/ϵ3 genotype was present in any patient within the well-differentiated LUAD group. The *APOE* ϵ2/ϵ4 genotype showed no significant relationship with the probability of developing LUAD with varying degrees of differentiation (**Table 7**).

**Table 7:** Binary logistic regression analysis of *ApoE* genotypes frequencies in the control group and LUAD subgroups by differentiation level.

LUAD	Genotype	OR	95%CI	*p*-Value
Poorly	*ApoE* ϵ2ϵ2	6.52	2.49-17.07	**0.000**
	*ApoE* ϵ2ϵ3	6.80	3.24-14.26	**0.000**
	*ApoE* ϵ3ϵ3	0.56	0.33-0.96	**0.033**
	*ApoE* ϵ2ϵ4	0.54	0.12-2.35	0.411
	*ApoE* ϵ3ϵ4	–	–	–
	*ApoE* ϵ4ϵ4	–	–	–
Moderately	*ApoE* ϵ2ϵ2	4.68	1.68-13.00	**0.003**
	*ApoE* ϵ2ϵ3	3.22	1.38-7.54	**0.007**
	*ApoE* ϵ3ϵ3	0.94	1.38-7.54	0.822
	*ApoE* ϵ2ϵ4	0.52	0.12-2.25	0.378
	*ApoE* ϵ3ϵ4	0.20	0.05-0.84	**0.028**
	*ApoE* ϵ4ϵ4	–	–	–
Well	*ApoE* ϵ2ϵ2	–	–	–
	*ApoE* ϵ2ϵ3	–	–	–
	*ApoE* ϵ3ϵ3	4.96	1.75-14.01	**0.003**
	*ApoE* ϵ2ϵ4	0.31	0.04-2.36	0.259
	*ApoE* ϵ3ϵ4	0.25	0.06-1.04	0.056
	*ApoE* ϵ4ϵ4	0.75	0.09-6.02	0.790

Bold P-values indicate statistically significant differences (P < 0.05).

### 3.5 The lipid profile between individuals carrying ϵ2, ϵ3, and ϵ4 alleles in the control and LUAD subgroups

In LUAD patients, individuals carrying the ϵ2 and ϵ3 allele exhibited significant alterations in lipid metabolism compared to the healthy control group, with LDL, HDL, total cholesterol (TC), APOB, and FFA levels downregulated (P<0.05). In contrast, no significant differences were observed in patients carrying the ϵ4 allele (**Table 8**; **Supplementary Figures 1A–C** **).**

**Table 8:** The lipid profile between individuals carrying ϵ2, ϵ3, and ϵ4 alleles in the control and LUAD group.

	ϵ2		ϵ3		ϵ4	
	M ± SEM (LUAD *VS* Control)	P Value	M ± SEM (LUAD *VS* Control)	P Value	M ± SEM (LUAD *VS* Control)	P Value
TG (mmol/L)	1.98 ± 0.09 vs 2.15 ± 0.14	0.671	2.13 ± 0.06 vs 1.88 ± 0.04	**0.000**	1.80 ± 0.23 vs 1.76 ± 0.07	0.874
LDL (mmol/L)	2.83 ± 0.13 vs 3.27 ± 0.17	**0.041**	2.91 ± 0.06 vs 2.95 ± 0.05	0.601	2.30 ± 0.37 vs 2.86 ± 0.11	0.156
HDL (mmol/L)	1.71 ± 0.15 vs 3.83 ± 0.31	**0.000**	2.26 ± 0.11 vs 2.65 ± 0.09	**0.025**	3.38 ± 0.63 vs 2.66 ± 0.21	0.330
TC (mmol/L)	2.16 ± 0.24 vs 3.17 ± 0.38	**0.021**	2.47 ± 0.14 vs 2.97 ± 0.11	**0.030**	4.31 ± 1.36 vs 2.74 ± 0.21	0.445
APOA1	1.39 ± 0.14 vs 1.80 ± 0.19	0.086	1.54 ± 0.08 vs 2.10 ± 0.06	**0.002**	1.46 ± 0.11 vs 2.36 ± 0.13	0.131
APOB	1.02 ± 0.07 vs 1.99 ± 0.07	**0.000**	1.36 ± 0.05 vs 1.80 ± 0.03	**0.000**	1.43 ± 0.31 vs 1.88 ± 0.07	0.070
Lp(a)(10^2mmol/L)	2.47 ± 0.28 vs 4.75 ± 0.81	**0.002**	2.85 ± 0.15 vs 3.89 ± 0.14	**0.000**	2.66 ± 0.98 vs 3.75 ± 0.25	0.231
FFA	0.38 ± 0.05 vs 0.83 ± 0.07	**0.000**	0.45 ± 0.02 vs 0.55 ± 0.02	**0.000**	0.54 ± 0.14 vs 0.64 ± 0.04	0.520

Bold P-values indicate statistically significant differences (P < 0.05).

To further explore the effects of different *APOE* alleles on lipid profiles in LUAD patients and the modulatory role of disease stage, with comparisons to a normal control group. In early-stage LUAD patients, ϵ3 and ϵ2 allele carriers were associated with the most significant alterations in lipid metabolism: ϵ3 carriers exhibited increased TG, HDL,APOA1 and ApoB levels and decreased Lp(α) and FFA levels, whereas, ϵ2 carriers had lower HDL and FFA levels and higher ApoB levels, ϵ4 carriers had significant changes only in increased ApoB levels (P < 0.05) (**Table 9A**; **Supplementary Figure 2A–C**). In late-stage LUAD patients, ϵ2 carriers primarily showed significant reductions in lipoproteins and FFA, whereas ϵ3 carriers demonstrated significant increases in TG levels along with decreased TC,APOB and FFA levels (P < 0.05) (**Table 9B**; **Supplementary Figures 2D–F**).

**Table 9:** Lipid profiles of individuals carrying ϵ2, ϵ3, and ϵ4 alleles in LUAD with different stages and control group.

A. Lipid profiles in ϵ2, ϵ3, and ϵ4 allele carriers between early LUAD and control group						
	ϵ2		ϵ3		ϵ4	
	M ± SEM (LUAD VS Control)	P Value	M ± SEM (LUAD VS Control)	P Value	M ± SEM (LUAD VS Control)	P Value
TG (mmol/L)	2.02 ± 0.18 vs 2.15 ± 0.14	0.561	2.19 ± 0.08 vs 1.88 ± 0.04	**0.000**	1.91 ± 0.28 vs 1.76 ± 0.07	0.625
LDL (mmol/L)	2.15 ± 0.14 vs 2.96 ± 0.24	0.297	1.88 ± 0.04 vs 2.96 ± 0.09	0.937	1.76 ± 0.07 vs 1.84 ± 0.34	**0.041**
HDL (mmol/L)	3.27 ± 0.17 vs 1.58 ± 0.09	**0.000**	2.95 ± 0.05 vs 2.22 ± 0.15	**0.037**	2.86 ± 0.11 vs 3.01 ± 1.04	0.713
TC (mmol/L)	3.83 ± 0.31 vs 1.89 ± 0.32	**0.029**	2.65 ± 0.09 vs 2.78 ± 0.22	0.453	2.66 ± 0.21 vs 3.39 ± 1.42	0.515
APOA1	3.17 ± 0.38 vs 1.58 ± 0.37	0.576	2.97 ± 0.11 vs 1.51 ± 0.11	**0.000**	2.74 ± 0.21 vs 1.55 ± 0.03	0.172
APOB	1.80 ± 0.19 vs 1.02 ± 0.11	**0.000**	2.10 ± 0.06 vs 1.36 ± 0.08	**0.000**	2.36 ± 0.13 vs 1.11 ± 0.43	**0.017**
Lp(a)(10^2mmol/L)	3.00 ± 0.45 vs 4.75 ± 0.81	0.126	2.82 ± 0.19 vs 3.89 ± 0.14	**0.000**	3.48 ± 1.53 vs 3.75± 0.25	0.814
FFA	0.31 ± 0.07 vs 0.83 ± 0.07	**0.000**	0.43 ± 0.03 vs 0.55 ± 0.02	**0.001**	0.45 ± 0.19 vs 0.64 ± 0.04	0.339

B. Lipid profiles of ϵ2, ϵ3, and ϵ4 allele carriers between late LUAD and control group						
	ϵ2		ϵ3		ϵ4	
	M ± SEM (LUADVS Control)	P Value	M ± SEM (LUADVS Control)	P Value	M ± SEM (LUADVS Control)	P Value
TG (mmol/L)	1.96 ± 0.10 vs 2.15 ± 0.14	0.275	2.08 ± 0.08 vs 1.88 ± 0.04	**0.021**	1.62 ± 0.47 vs 1.76 ± 0.07	0.730
LDL (mmol/L)	2.75 ± 0.15 vs 3.27 ± 0.17	**0.041**	2.86 ± 0.09 vs 2.95 ± 0.05	0.375	2.99 ± 0.49 vs 2.86 ± 0.11	0.822
HDL (mmol/L)	1.79 ± 0.24 vs 3.83 ± 0.31	**0.000**	2.29 ± 0.16 vs 2.65 ± 0.09	0.085	3.94 ± 0.49 vs 2.66 ± 0.21	0.263
TC (mmol/L)	2.31 ± 0.32 vs 3.17 ± 0.38	**0.021**	2.16 ± 0.16 vs 2.97 ± 0.11	**0.001**	5.70 ± 3.03 vs 2.74 ± 0.21	**0.020**
APOA1	1.29 ± 0.06 vs 1.80 ± 0.19	0.086	1.57 ± 0.11 vs 2.10 ± 0.06	**0.000**	1.31 ± 0.29 vs 2.36 ± 0.13	0.148
APOB	1.01 ± 0.09 vs 1.99 ± 0.07	**0.000**	1.37 ± 0.07 vs 1.80 ± 0.03	**0.000**	1.90 ± 0.00 vs 1.88 ± 0.07	0.967
Lp(a)(10^2mmol/L)	2.17 ± 0.35 vs 4.75 + 0.81	**0.002**	2.89 ± 0.23 vs 3.89 + 0.14	**0.001**	1.44 ± 0.33 vs 3.75 + 0.25	0.095
FFA	0.42 ± 0.06 vs 0.83 ± 0.07	**0.000**	0.47 ± 0.03 vs 0.55 ± 0.02	**0.018**	0.67 ± 0.24 vs 0.64 ± 0.04	0.884

Bold P-values indicate statistically significant differences (P < 0.05).

To explore the relationship between *APOE* alleles and lipid profiles in LUAD patients with different levels of differentiation, and to compare these findings with those from a normal control group. Specifically, in well-differentiated LUAD patients, ϵ3 carriers exhibited increased TG levels, along with significantly decreased levels of HDL, Apolipoprotein A1(ApoA1), Apolipoprotein B (APOB) Lipoprotein (Lp(a)), and free fatty acids (FFA). In ϵ4 carriers, significant reductions were primarily observed in LDL, ApoA1, and ApoB levels (P < 0.05) (**Table 10A**; **Supplementary Figures 3A, B**). In moderately differentiated LUAD patients, both ε2 and ε3 carriers showed notable lipid metabolic abnormalities, including HDL, APOA1, APOB and FFA levels. In contrast, no significant changes were noted in ϵ4 carriers, suggesting a minimal or insignificant effect of the ϵ4 allele on lipid metabolism in this subgroup (P < 0.05) (**Table 10B**; **Supplementary Figures 3C–E**). In poorly differentiated LUAD patients, those carrying ϵ2 and ϵ3 allele experienced significant disruptions in lipid metabolism, particularly with reductions in ApoB and Lp(a) levels, which were consistently observed in both groups (P < 0.05) (**Table 10C**; **Supplementary Figures 3F, G**). These results suggest that *APOE* alleles may significantly affect lipid metabolism, especially in individuals with poorly differentiated LUAD.

**Table 10:** Lipid profiles of APOE ϵ2, ϵ3, and ϵ4 allele carriers within LUAD subgroups by differentiation level and control group.

A. Lipid profiles of ϵ3, and ϵ4 allele carriers in well differentiated LUAD and control group				
	ϵ3		ϵ4	
	M ± SEM (LUADVS Control)	P Value	M ± SEM (LUADVS Control)	P Value
TG (mmol/L)	2.25 ± 0.08 vs 1.88 ± 0.04	**0.000**	1.84 ± 0.35 vs 1.76 ± 0.07	0.795
LDL (mmol/L)	2.88 ± 0.10 vs 2.95 ± 0.05	0.574	1.84 ± 0.35 vs 2.86 ± 0.11	**0.043**
HDL (mmol/L)	2.13 ± 0.16 vs 2.65 ± 0.09	**0.007**	2.85 ± 0.90 vs 2.66 ± 0.21	0.839
TC (mmol/L)	2.57 ± 0.24 vs 2.97 ± 0.11	0.157	3.20 ± 1.44 vs 2.74 ± 0.21	0.648
APOA1	1.51 ± 0.12 vs 2.10 ± 0.06	**0.000**	1.55 ± 0.03 vs 2.36 ± 0.13	**0.000**
APOB	1.36 ± 0.08 vs 1.80 ± 0.03	**0.000**	1.12 ± 0.44 vs 1.88 ± 0.07	**0.018**
Lp(a)(10^2mmol/L)	3.14 ± 0.27 vs 3.89 ± 0.14	**0.030**	3.03 ± 1.74 vs 3.75 ± 0.25	0.537
FFA	0.42 ± 0.04 vs 0.55 + 0.02	**0.001**	0.34 ± 0.11 vs 0.64 + 0.04	0.126

B. Lipid profiles of *APOE* ϵ2, ϵ3, and ϵ4 allele carriers with moderately differentiated LUAD and control group						
	ϵ2		ϵ3		ϵ4	
	M ± SEM (LUADVS Control)	P Value	M ± SEM (LUADVS Control)	P Value	M ± SEM (LUADVS Control)	P Value
TG (mmol/L)	2.08 ± 0.09 vs 2.15 ± 0.14	0.671	2.06 ± 0.09 vs 1.88 ± 0.04	0.070	1.73 ± 0.36 vs 1.76 ± 0.07	0.947
LDL (mmol/L)	2.76 ± 0.19 vs 3.27 ± 0.17	0.064	2.78 ± 0.11 vs 2.95 ± 0.05	0.175	2.98 ± 0.50 vs 2.86 ± 0.11	0.835
HDL (mmol/L)	2.00 ± 0.31 vs 3.83 ± 0.31	**0.000**	2.18 ± 0.18 vs 2.65 ± 0.09	**0.025**	4.17 ± 0.72 vs 2.66 ± 0.21	0.187
TC (mmol/L)	2.08 ± 0.42 vs 3.17 ± 0.38	0.072	2.41 ± 0.23 vs 2.97 ± 0.11	**0.03**	5.99 ± 2.74 vs 2.74 ± 0.21	0.445
APOA1	1.28 ± 0.06 vs 1.80 ± 0.19	**0.049**	1.57 ± 0.15 vs 2.10 ± 0.06	**0.002**	1.32 ± 0.30 vs 2.36 ± 0.13	0.131
APOB	1.06 ± 0.15 vs 1.99 ± 0.07	**0.000**	1.34 ± 0.10 vs 1.80 ± 0.03	**0.000**	1.89 ± 0.01 vs 1.88 ± 0.07	0.989
Lp(a)(10^2mmol/L)	2.60 ± 0.56 vs 4.75 ± 0.81	0.063	2.79 ± 0.25 vs 3.89 ± 0.14	**0.000**	2.11 ± 0.35 vs 3.75 ± 0.25	0.232
FFA	0.37 ± 0.07 vs 0.83 ± 0.07	**0.000**	0.42 ± 0.03 vs 0.55 ± 0.02	**0.000**	0.84 ± 0.08 vs 0.64 ± 0.04	0.394

C. Lipid profiles of ϵ2 and ϵ4 allele carriers with poorly differentiated LUAD and control group				
	ϵ2		ϵ3	
	M ± SEM (LUADVS Control)	P Value	M ± SEM (LUADVS Control)	P Value
TG (mmol/L)	1.92 ± 0.13 vs 2.15 ± 0.14	0.225	2.08 ± 0.12 vs 1.88 ± 0.04	0.070
LDL (mmol/L)	2.87 ± 0.17 vs 3.27 ± 0.17	0.107	3.10 ± 0.12 vs 2.95 ± 0.05	0.276
HDL (mmol/L)	1.53 ± 0.16 vs 3.83 ± 0.31	**0.000**	2.51 ± 0.21 vs 2.65 ± 0.09	0.599
TC (mmol/L)	2.21 ± 0.29 vs 3.17 ± 0.38	0.052	2.42 ± 0.27 vs 2.97 ± 0.11	0.079
APOA1	1.46 ± 0.22 vs 1.80 ± 0.19	0.255	1.55 ± 0.11 vs 2.10 ± 0.06	**0.000**
APOB	0.99 ± 0.07 vs 1.99 ± 0.07	**0.000**	1.41 ± 0.09 vs 1.80 ± 0.03	**0.000**
Lp(a)(10^2mmol/L)	2.39 ± 0.30 vs 4.75 ± 0.81	**0.008**	2.57 ± 0.24 vs 3.89 ± 0.14	**0.001**
FFA	0.38 ± 0.06 vs 0.83 ± 0.07	**0.000**	0.53 ± 0.05 vs 0.55 ± 0.02	0.625

Bold P-values indicate statistically significant differences (P < 0.05).

## 4 Discussion

Our research focused on assessing the role of *APOE* gene polymorphisms in LUAD susceptibility and their influence on lipid profiles. Previous reports have primarily focused on APOE protein expression levels in patients with lung cancer. This study demonstrates revealed that the presence of the ϵ2 allele increases the risk of LUAD, while the ϵ4 allele provide a protective effect. However, the *APOE* ϵ3/ϵ3 genotype did not exhibit a significant association with LUAD risk. The *APOE* ϵ2/ϵ4 genotype demonstrated no statistical difference between the healthy control and LUAD groups. Furthermore, the *APOE* ϵ2/ϵ2 genotype displayed a higher risk of cancer development compared to *APOE* ϵ2/ϵ3, particularly in patients with advanced stages of lung cancer, suggesting a potential association with cancer progression stages. Similar studies have identified the *APOE* ϵ4 allele as a lung cancer risk factor. However, they overlook important differences in pathological subtypes and fail to address the unique metabolic characteristics of LUAD (19). The role of the *APOE* alleles and their genotypes varies across different tumor types. For instance, *APOE* ϵ2 reduces the risk of developing colorectal cancer, while the *APOE* ϵ4 allele does not significantly impact colorectal cancer risk (22), In contrast, the *APOE* ϵ3 allele is linked to a higher risk of laryngeal squamous cell carcinoma (LSCC) (23). Different cancer susceptibility results are caused by the interaction between *APOE* allele carriers and their genotypes, which has a substantial impact on cancer development and progression.

The frequencies of different *APOE* genotype within the population are affected by gender, age and genetic variations (24). This study was conducted with baseline data such as gender, age, and medical history being consistent across the groups, which means that the potential confounding effects of these factors were eliminated in this study. Individuals carrying the *APOE* ϵ2/ϵ4 genotype do not have a significantly increased risk of LUAD, we hypothesize an allelic dosage effect for *APOE* ϵ2 and ϵ4, suggesting that the number of alleles correlates with the extent of their impact on phenotype and function. Specifically, ϵ2 appears to have a detrimental effect, while ϵ4 seems to exhibit a protective effect. The adverse impact of homozygous ϵ2/ϵ2 is more pronounced than that of ϵ2/ϵ3, whereas the protective effect of homozygous ϵ4/ϵ4 is stronger than that of ϵ3/ϵ4. In colorectal cancer, patients with the *APOE* ϵ2/ϵ3 genotype are more likely to progress to advanced stages once diagnosed (12). Individuals with the *APOE* ϵ2/ϵ4 and *APOE* ϵ3/ϵ4 genotypes have a reduced risk of LSCC by 2.9 and 1.5 times. Individuals with the APOE ϵ3/ϵ3 genotype have a 1.7-fold higher likelihood of developing LSCC (23). The findings align with the allelic dosage effect in cancer risk, but larger population studies are needed for definitive conclusions.

Recent studies suggest that interactions between lipid metabolism and *APOE* genotypes may increase cancer risk, particularly for *APOE* ϵ2 carriers with low TC levels (19, 25). The PROSPER-derived studies evaluated the relationship between *APOE* subtypes and cancer risk in the older population. These studies indicated a negative correlation between TC levels and cancer incidence and mortality (26). The study couldn’t establish a direct causal link between *APOE* genotype and cancer risk due to the effect of medications on TC levels in patients with vascular disease (27). The primary outcome variable of the study was cancer development, without differentiating between specific types of malignancies, however, the analysis of *APOE* gene polymorphism and tumor risk did not include the Chinese population (28). *APOE* ϵ4 female patients display elevated TG levels and an increased risk of breast cancer (29, 30). Interactions between *APOE* genotypes and lipid metabolism may impact cancer risk, though direct causality and population-specific effects remain unclear. The precise mechanisms through which *APOE* genotypes influence malignant tumor progression remain unclear. Some studies suggest that the ϵ2 allele may protect against head and neck cancer, potentially because of its enhanced antioxidant abilities (31), however, overexpression of APOE2 can induce epithelial-mesenchymal transition (EMT), thereby promoting cancer progression in pancreatic cancer (32). Mice carrying the human *APOE* ϵ2 allele demonstrate prolonged progression-free survival (PFS) compared to *APOE* ϵ4 carriers, with the mechanism linked to diminished T-cell cytotoxicity against tumor cells (11).Building on mechanisms observed in other cancers, these results underscore the importance of investigating how *APOE* ϵ2 may drive tumor progression in LUAD.

APOE gene can influence the uptake and breakdown of TC and TG in lung cancer patients (33, 34). Patients with LUAD carrying the ϵ2 allele showed decreased TC levels. TC levels increase in early-stage LUAD patients and are reduced in late-stage patients compared to those with the ϵ4 allele. A condition called the cancer preclinical phenomenon when cancer causes a drop in plasma TC levels prior to a formal cancer diagnosis, this effect arises as cancer cells increase their uptake of TC from the bloodstream for growth and proliferation (35, 36). Earlier studies have indicated a heightened gastric cancer incidence in individuals carrying the ϵ2 allele with lower TC levels (37), a study conducted across seven countries found that lung cancer mortality risk rises when TC levels drop below 170 mg/dL (25). The *APOE* ϵ2 allele affects different lipid metabolism markers across various tumors, ApoA1 levels are significantly increased in patients with early-stage gastric cancer, whereas they remain relatively stable in those with advanced-stage gastric cancer (38, 39). Increased APOA1 levels can reduce the risk of lung cancer (40), but after lung cancer patients with brain metastases, APOA1 levels are up-regulated (41). Patients carrying the *APOE* ϵ2 allele tend to have lower VLDL-C levels, while those with the ϵ4 allele exhibit increased VLDL-C levels (18). This association suggests that the phenotypic variation of lipoproteins among individuals can alter the cancer risk by modulating serum lipoprotein levels. Overall, *APOE* gene polymorphisms influence lipid metabolism in LUAD patients, characterized by reduced HDL levels and elevated TG (42), which provide essential energy and biosynthetic precursors for tumor cell proliferation. Moreover, dysregulated lipid metabolism impacts immune cell function, impairing tumor immune surveillance, such as cholesterol modulating T cell receptor signaling and immune checkpoint expression (43). Thus, targeting lipid metabolism could represent a novel strategy to enhance immune response and treat tumors​. FFA and cholesterol can influence multiple stages of cancer immunity by modifying the state and function of immune cells in the tumor microenvironment (TME) (44, 45). These changes interfere with the production of tumor-associated antigens (TAAs) (43), enabling tumor cells to escape immune surveillance and impairing the normal antitumor response (46). The study indicates that macrophages with high APOE expression are enriched in metastatic gastric tumors, contributing to immune evasion by reducing CD8+ T cell infiltration (47). Targeted interventions that modulate these pathways could potentially reduce tumor growth and improve treatment outcomes for APOE ϵ2 carriers. Additionally, since APOE ϵ2 is implicated in the regulation of epithelial–mesenchymal transition (EMT), therapies aimed at inhibiting EMT may offer significant benefits for these patients. Identifying APOE ϵ2 carriers would allow clinicians to personalize treatment strategies, potentially improving prognosis and enhancing the effectiveness of therapies for advanced LUAD.

This study has certain limitations. We explored the association between *APOE* genotypes and TNM staging in patients with LUAD. However, subgroup analyses for the T stage, N stage, and M stage for each patient were not conducted. This limitation stems from the fact that not all patients could undergo accurate T, N, and M staging, resulting in significant data loss and reduced statistical power. Moreover, this study did not measure *APOE* serum concentrations. It would have been beneficial to analyze the correlation between *APOE* levels and lung cancer staging if such data had been available. This study focused on a specific population in the Qingdao area of Shandong Province, China. Future research should include data from other regions or populations with different genetic backgrounds to validate the generalizability of the association between APOE polymorphisms and LUAD.

## 5 Conclusion

Individuals carrying the ϵ2 allele have an increased susceptibility to developing LUAD, accompanied by disrupted lipid metabolism. The *APOE* ϵ2/ϵ2 and ϵ2/ϵ3 genotypes have been attributed to an increased probability of advanced and poorly differentiated LUAD.

## Data Availability

The datasets presented in this study can be found in online repositories. The names of the repository/repositories and accession number(s) can be found in the article/**Supplementary Material**.

## References

[1] Dongés B Haupt LM Lea RA Chan RC Shum DH Griffiths LR. Role of the apolipoprotein E and catechol-O-methyltransferase genes in prospective and retrospective memory traits. Gene. (2012) 506:135–40. doi: 10.1016/j.gene.2012.06.067 22771913

[2] Xu M Zhao J Zhang Y Ma X Dai Q Zhi H. Apolipoprotein E gene variants and risk of coronary heart disease: A meta-analysis. BioMed Res Int. (2016) 2016:3912175. doi: 10.1155/2016/3912175 27868062 PMC5102878

[3] Serrano-Pozo A Das S Hyman BT. APOE and Alzheimer's disease: advances in genetics, pathophysiology, and therapeutic approaches. Lancet Neurol. (2021) 20:68–80. doi: 10.1016/S1474-4422(20)30412-9 33340485 PMC8096522

[4] Jackson RJ Keiser MS Meltzer JC Fykstra DP Dierksmeier SE Hajizadeh S. APOE2 gene therapy reduces amyloid deposition and improves markers of neuroinflammation and neurodegeneration in a mouse model of Alzheimer disease. Mol Ther. (2024) 32:1373–86. doi: 10.1016/j.ymthe.2024.03.024 PMC11081918 38504517

[5] Lumsden AL Mulugeta A Zhou A Hyppönen E. Apolipoprotein E (APOE) genotype-associated disease risks: a phenome-wide, registry-based, case-control study utilising the UK Biobank. EBioMedicine. (2020) 59:102954. doi: 10.1016/j.ebiom.2020.102954 32818802 PMC7452404

[6] Rasmussen KL Tybjærg-Hansen A Nordestgaard BG Frikke-Schmidt R. Plasma levels of apolipoprotein E, APOE genotype, and all-cause and cause-specific mortality in 105 949 individuals from a white general population cohort. Eur Heart J. (2019) 40:2813–24. doi: 10.1093/eurheartj/ehz402 PMC6735871 31236578

[7] Innes H Nischalke HD Guha IN Weiss KH Irving W Gotthardt D. The rs429358 locus in apolipoprotein E is associated with hepatocellular carcinoma in patients with cirrhosis. Hepatol Commun. (2022) 6:1213–26. doi: 10.1002/hep4.1886 PMC9035556 34958182

[8] Amidi A Agerbæk M Wu LM Pedersen AD Mehlsen M Clausen CR. Changes in cognitive functions and cerebral grey matter and their associations with inflammatory markers, endocrine markers, and APOE genotypes in testicular cancer patients undergoing treatment. Brain Imaging Behav. (2017) 11:769–83. doi: 10.1007/s11682-016-9552-3 27240852

[9] Wang H Zhou HC Ren RL Du SX Guo ZK Shen XH. Apolipoprotein E2 inhibits mitochondrial apoptosis in pancreatic cancer cells through ERK1/2/CREB/BCL-2 signaling. Hepatobiliary Pancreat Dis Int. (2023) 22:179–89. doi: 10.1016/j.hbpd.2022.09.010 36243659

[10] Adaku N Ostendorf BN Mei W Tavazoie SF. Apolipoprotein E2 stimulates protein synthesis and promotes melanoma progression and metastasis. Cancer Res. (2023) 83:3013–25. doi: 10.1158/0008-5472.CAN-23-1252 PMC10740391 37335131

[11] Ostendorf BN Bilanovic J Adaku N Tafreshian KN Tavora B Vaughan RD. Common germline variants of the human APOE gene modulate melanoma progression and survival. Nat Med. (2020) 26:1048–53. doi: 10.1038/s41591-020-0879-3 PMC8058866 32451497

[12] Torres GG Dose J Hasenbein TP Nygaard M Krause-Kyora B Mengel-From J. Long-lived individuals show a lower burden of variants predisposing to age-related diseases and a higher polygenic longevity score. Int J Mol Sci. (2022) 23:10949. doi: 10.3390/ijms231810949 36142858 PMC9504529

[13] Hsiao CP Goto T Von Ah D Saligan LN. Cancer-Related Cognitive Impairment Associated with APOE rs7412 and BDNF rs6265 in Breast Cancer Survivors. Semin Oncol Nurs. (2024) 40:151721. doi: 10.1016/j.soncn.2024.151721 39198096

[14] Ahles TA Orlow I Schofield E Li Y Ryan E Root JC. The impact of APOE and smoking history on cognitive function in older, long-term breast cancer survivors. J Cancer Surviv. (2024) 18:575–85. doi: 10.1007/s11764-022-01267-z PMC10123173 36279076

[15] Bian X Liu R Meng Y Xing D Xu D Lu Z. Lipid metabolism and cancer. J Exp Med. (2021) 218:e20201606. doi: 10.1084/jem.20201606 33601415 PMC7754673

[16] Martínez-Martínez AB Torres-Perez E Devanney N Del Moral R Johnson LA Arbones-Mainar JM. Beyond the CNS: The many peripheral roles of APOE. Neurobiol Dis. (2020) 138:104809. doi: 10.1016/j.nbd.2020.104809 32087284 PMC7195217

[17] Zhang ZG Li Y Ng CT Song YQ. Inflammation in alzheimer's disease and molecular genetics: recent update. Arch Immunol Ther Exp (Warsz). (2015) 63:333–44. doi: 10.1007/s00005-015-0351-0 26232392

[18] Bennet AM Di Angelantonio E Ye Z Wensley F Dahlin A Ahlbom A. Association of apolipoprotein E genotypes with lipid levels and coronary risk. Jama. (2007) 298:1300–11. doi: 10.1001/jama.298.11.1300 17878422

[19] Gan C Zhang Y Liang F Guo X Zhong Z. Effects of APOE gene ϵ4 allele on serum lipid profiles and risk of cardiovascular disease and tumorigenesis in southern Chinese population. World J Surg Oncol. (2022) 20:280. doi: 10.1186/s12957-022-02748-2 36057714 PMC9440530

[20] Rinella ME. Nonalcoholic fatty liver disease: a systematic review. Jama. (2015) 313:2263–73. doi: 10.1001/jama.2015.5370 26057287

[21] Huang J Osarogiagbon RU Giroux DJ Nishimura KK Bille A Cardillo G. The international association for the study of lung cancer staging project for lung cancer: proposals for the revision of the N descriptors in the forthcoming ninth edition of the TNM classification for lung cancer. J Thorac Oncol. (2024) 19:766–85. doi: 10.1016/j.jtho.2023.10.012 PMC12323887 37866624

[22] Watson MA Gay L Stebbings WS Speakman CT Bingham SA Loktionov A. Apolipoprotein E gene polymorphism and colorectal cancer: gender-specific modulation of risk and prognosis. Clin Sci (Lond). (2003) 104:537–45. doi: 10.1042/CS20020329 12529167

[23] Liutkeviciene R Auzelyte J Liutkevicius V Vilkeviciute A Gedvilaite G Vaiciulis P. The role of apoE serum levels and apoE gene polymorphisms in patients with laryngeal squamous cell carcinoma. Biomolecules. (2022) 12:1013. doi: 10.3390/biom12081013 35892323 PMC9331506

[24] Kulminski AM Culminskaya I Arbeev KG Ukraintseva SV Arbeeva L Yashin AI. Trade-off in the effect of the APOE gene on the ages at onset of cardiocascular disease and cancer across ages, gender, and human generations. Rejuvenation Res. (2013) 16:28–34. doi: 10.1089/rej.2012.1362 23094790 PMC3582279

[25] Katan MB. Apolipoprotein E isoforms, serum cholesterol, and cancer. Lancet. (1986) 1:507–8. doi: 10.1016/S0140-6736(86)92972-7 2869248

[26] Trompet S Jukema JW Katan MB Blauw GJ Sattar N Buckley B. Apolipoprotein e genotype, plasma cholesterol, and cancer: a Mendelian randomization study. Am J Epidemiol. (2009) 170:1415–21. doi: 10.1093/aje/kwp294 19889709

[27] Wang C Najm R Xu Q Jeong DE Walker D Balestra ME. Gain of toxic apolipoprotein E4 effects in human iPSC-derived neurons is ameliorated by a small-molecule structure corrector. Nat Med. (2018) 24:647–57. doi: 10.1038/s41591-018-0004-z PMC5948154 29632371

[28] Keys A Aravanis C Blackburn H Buzina R Dontas AS Fidanza F. Serum cholesterol and cancer mortality in the Seven Countries Study. Am J Epidemiol. (1985) 121:870–83. doi: 10.1093/oxfordjournals.aje.a114057 4014179

[29] Moysich KB Freudenheim JL Baker JA Ambrosone CB Bowman ED Schisterman EF. Apolipoprotein E genetic polymorphism, serum lipoproteins, and breast cancer risk. Mol Carcinog. (2000) 27:2–9. doi: 10.1002/(SICI)1098-2744(200001)27:1<2::AID-MC2>3.0.CO;2-W 10642431

[30] Llanos AA Makambi KH Tucker CA Wallington SF Shields PG Adams-Campbell LL. Cholesterol, lipoproteins, and breast cancer risk in African American women. Ethn Dis. (2012) 22:281–7. Available online at: https://pubmed.ncbi.nlm.nih.gov/22870570/ PMC3830419 22870570

[31] De Feo E Rowell J Cadoni G Nicolotti N Arzani D Giorgio A. A case-control study on the effect of apoliprotein E genotype on head and neck cancer risk. Cancer Epidemiol Biomarkers Prev. (2010) 19:2839–46. doi: 10.1158/1055-9965.EPI-10-0624 20861397

[32] Wang H Du S Cai J Wang J Shen X. Apolipoprotein E2 promotes the migration and invasion of pancreatic cancer cells via activation of the ERK1/2 signaling pathway. Cancer Manag Res. (2020) 12:13161–71. doi: 10.2147/CMAR.S284115 PMC7764636 33376407

[33] Mahley RW. Apolipoprotein E: cholesterol transport protein with expanding role in cell biology. Science. (1988) 240:622–30. doi: 10.1126/science.3283935 3283935

[34] Marais AD. Apolipoprotein E in lipoprotein metabolism, health and cardiovascular disease. Pathology. (2019) 51:165–76. doi: 10.1016/j.pathol.2018.11.002 30598326

[35] Lyu Z Li N Wang G Feng X Chen S Su K. Independent and joint associations of blood lipids and lipoproteins with lung cancer risk in Chinese males: A prospective cohort study. Int J Cancer. (2019) 144:2972–84. doi: 10.1002/ijc.v144.12 30536993

[36] Kritchevsky SB Kritchevsky D. Serum cholesterol and cancer risk: an epidemiologic perspective. Annu Rev Nutr. (1992) 12:391–416. doi: 10.1146/annurev.nu.12.070192.002135 1503812

[37] Kang R Li P Wang T Li X Wei Z Zhang Z. Apolipoprotein E epsilon 2 allele and low serum cholesterol as risk factors for gastric cancer in a Chinese Han population. Sci Rep. (2016) 6:19930. doi: 10.1038/srep19930 26817942 PMC4730152

[38] Patel KK Kashfi K. Lipoproteins and cancer: The role of HDL-C, LDL-C, and cholesterol-lowering drugs. Biochem Pharmacol. (2022) 196:114654. doi: 10.1016/j.bcp.2021.114654 34129857 PMC8665945

[39] Shi F Wu H Qu K Sun Q Li F Shi C. Identification of serum proteins AHSG, FGA and APOA-I as diagnostic biomarkers for gastric cancer. Clin Proteomics. (2018) 15:18. doi: 10.1186/s12014-018-9194-0 29719494 PMC5925839

[40] Katzke VA Sookthai D Johnson T Kühn T Kaaks R. Blood lipids and lipoproteins in relation to incidence and mortality risks for CVD and cancer in the prospective EPIC-Heidelberg cohort. BMC Med. (2017) 15:218. doi: 10.1186/s12916-017-0976-4 29254484 PMC5735858

[41] Marchi N Mazzone P Fazio V Mekhail T Masaryk T Janigro D. ProApolipoprotein A1: a serum marker of brain metastases in lung cancer patients. Cancer. (2008) 112:1313–24. doi: 10.1002/cncr.v112:6 PMC2775530 18257091

[42] Yang W Bai Y Xiong Y Zhang J Chen S Zheng X. Potentiating the antitumour response of CD8(+) T cells by modulating cholesterol metabolism. Nature. (2016) 531:651–5. doi: 10.1038/nature17412 PMC4851431 26982734

[43] Zheng M Zhang W Chen X Guo H Wu H Xu Y. The impact of lipids on the cancer-immunity cycle and strategies for modulating lipid metabolism to improve cancer immunotherapy. Acta Pharm Sin B. (2023) 13:1488–97. doi: 10.1016/j.apsb.2022.10.027 PMC10149904 37139414

[44] Coutzac C Jouniaux JM Paci A Schmidt J Mallardo D Seck A. Systemic short chain fatty acids limit antitumor effect of CTLA-4 blockade in hosts with cancer. Nat Commun. (2020) 11:2168. doi: 10.1038/s41467-020-16079-x 32358520 PMC7195489

[45] King RJ Singh PK Mehla K. The cholesterol pathway: impact on immunity and cancer. Trends Immunol. (2022) 43:78–92. doi: 10.1016/j.it.2021.11.007 34942082 PMC8812650

[46] Wang Y Wang Y Ren Y Zhang Q Yi P Cheng C. Metabolic modulation of immune checkpoints and novel therapeutic strategies in cancer. Semin Cancer Biol. (2022) 86:542–65. doi: 10.1016/j.semcancer.2022.02.010 35151845

[47] Dong Y Hu K Zhang J Zhu M Liu M Yuan Y. ScRNA-seq of gastric cancer tissues reveals differences in the immune microenvironment of primary tumors and metastases. Oncogene. (2024) 43:1549–64. doi: 10.1038/s41388-024-03012-5 38555278

