# Supplementary material for: The role of APOE gene polymorphisms in lung adenocarcinoma susceptibility and lipid profile
Source: Front Immunol. 2024 Dec 23;15:1522761. doi: 10.3389/fimmu.2024.1522761 (PMC11701022; doi:10.3389/fimmu.2024.1522761)
Supplement: Supplementary file 1 [file DataSheet1.docx]

LUAD group

>Seq1 [organism=Homo sapiens] LUAD ApoE ε2ε2

ATCCTAGCGCCCAGCGGCTGGGCGCGGAATGGAGGACGTGTGCGGCCGCCTGGTGCAGTACCGCGGCGAGGTGCAGGCCATGCTCGGCCAGAGCACCGAGGAGCTGCGGGTGCGCCTCGCCTCCCACCTGCGCAAGCTGCGTAAGCGGCTCCTCCGCGATGCCGATGACCTGCAGAAGTGCCTGGCAGTGTACCAGGCCGGGGCGAAT

>Seq2 [organism=Homo sapiens] LUAD ApoE ε2ε2

GGGCGGGAGTCGGCTGGGCGCGGAATGGAGGACGTGTGCGGCCGCCTGGTGCAGTACCGCGGCGAGGTGCAGGCCATGCTCGGCCAGAGCACCGAGGAGCTGCGGGTGCGCCTCGCCTCCCACCTGCGCAAGCTGCGTAAGCGGCTCCTCCGCGATGCCGATGACCTGCAGAAGTGCCTGGCAGTGTACCAGGCCGGGGCGAATAA

>Seq3 [organism=Homo sapiens] LUAD ApoE ε2ε2

GCCCAGCGGCTGGGCGCGGAATGGAGGACGTGTGCGGCCGCCTGGTGCAGTACCGCGGCGAGGTGCAGGCCATGCTCGGCCAGAGCACCGAGGAGCTGCGGGTGCGCCTCGCCTCCCACCTGCGCAAGCTGCGTAAGCGGCTCCTCCGCGATGCCGATGACCTGCAGAAGTGCCTGGCAGTGTACCAGGCCGGGGCGAATGCGAATAA

>Seq4 [organism=Homo sapiens] LUAD ApoE ε2ε2

GGCCCAGCGGCTGGGCGCGGAATGGAGGACGTGTGCGGCCGCCTGGTGCAGTACCGCGGCGAGGTGCAGGCCATGCTCGGCCAGAGCACCGAGGAGCTGCGGGTGCGCCTCGCCTCCCACCTGCGCAAGCTGCGTAAGCGGCTCCTCCGCGATGCCGATGACCTGCAGAAGTGCCTGGCAGTGTACCAGGCCGGGGCGAATGCGAATAA

>Seq5 [organism=Homo sapiens] LUAD ApoE ε2ε2

AAGACGCGACGTGCGGCTGGGCGCGGAATGGAGGACGTGTGCGGCCGCCTGGTGCAGTACCGCGGCGAGGTGCAGGCCATGCTCGGCCAGAGCACCGAGGAGCTGCGGGTGCGCCTCGCCTCCCACCTGCGCAAGCTGCGTAAGCGGCTCCTCCGCGATGCCGATGACCTGCAGAAGTGCCTGGCAGTGTACCAGGCCGGGGCGAATAA

>Seq6 [organism=Homo sapiens] LUAD ApoE ε2ε2

CTGACGTAGCTACCGGCTGGGCGCGGAATGGAGGACGTGTGCGGCCGCCTGGTGCAGTACCGCGGCGAGGTGCAGGCCATGCTCGGCCAGAGCACCGAGGAGCTGCGGGTGCGCCTCGCCTCCCACCTGCGCAAGCTGCGTAAGCGGCTCCTCCGCGATGCCGATGACCTGCAGAAGTGCCTGGCAGTGTACCAGGCCGGGGCGAATAA

>Seq7 [organism=Homo sapiens] LUAD ApoE ε2ε2

AGCTAGCGTAGCACGGCTGGGCGCGGAATGGAGGACGTGTGCGGCCGCCTGGTGCAGTACCGCGGCGAGGTGCAGGCCATGCTCGGCCAGAGCACCGAGGAGCTGCGGGTGCGCCTCGCCTCCCACCTGCGCAAGCTGCGTAAGCGGCTCCTCCGCGATGCCGATGACCTGCAGAAGTGCCTGGCAGTGTACCAGGCCGGGGCGAATAA

>Seq8 [organism=Homo sapiens] LUAD ApoE ε2ε2

TGCGTAGCTAGCTCGGCTGGGCGCGGAATGGAGGACGTGTGCGGCCGCCTGGTGCAGTACCGCGGCGAGGTGCAGGCCATGCTCGGCCAGAGCACCGAGGAGCTGCGGGTGCGCCTCGCCTCCCACCTGCGCAAGCTGCGTAAGCGGCTCCTCCGCGATGCCGATGACCTGCAGAAGTGCCTGGCAGTGTACCAGGCCGGGGCGAATAA

>Seq9 [organism=Homo sapiens] LUAD ApoE ε2ε2

CGTAGCTAGCTAGCGGCTGGGCGCGGAATGGAGGACGTGTGCGGCCGCCTGGTGCAGTACCGCGGCGAGGTGCAGGCCATGCTCGGCCAGAGCACCGAGGAGCTGCGGGTGCGCCTCGCCTCCCACCTGCGCAAGCTGCGTAAGCGGCTCCTCCGCGATGCCGATGACCTGCAGAAGTGCCTGGCAGTGTACCAGGCCGGGGCGAATAA

>Seq10 [organism=Homo sapiens] LUAD ApoE ε2ε2

GTAGCTAGCTAGCCGGCTGGGCGCGGAATGGAGGACGTGTGCGGCCGCCTGGTGCAGTACCGCGGCGAGGTGCAGGCCATGCTCGGCCAGAGCACCGAGGAGCTGCGGGTGCGCCTCGCCTCCCACCTGCGCAAGCTGCGTAAGCGGCTCCTCCGCGATGCCGATGACCTGCAGAAGTGCCTGGCAGTGTACCAGGCCGGGGCGAATAA

>Seq11 [organism=Homo sapiens] LUAD ApoE ε2ε2

TAGCTAGCTAGCTCGGCTGGGCGCGGAATGGAGGACGTGTGCGGCCGCCTGGTGCAGTACCGCGGCGAGGTGCAGGCCATGCTCGGCCAGAGCACCGAGGAGCTGCGGGTGCGCCTCGCCTCCCACCTGCGCAAGCTGCGTAAGCGGCTCCTCCGCGATGCCGATGACCTGCAGAAGTGCCTGGCAGTGTACCAGGCCGGGGCGAATAA

>Seq12 [organism=Homo sapiens] LUAD ApoE ε2ε2

AGCTAGCTAGCTACGGCTGGGCGCGGAATGGAGGACGTGTGCGGCCGCCTGGTGCAGTACCGCGGCGAGGTGCAGGCCATGCTCGGCCAGAGCACCGAGGAGCTGCGGGTGCGCCTCGCCTCCCACCTGCGCAAGCTGCGTAAGCGGCTCCTCCGCGATGCCGATGACCTGCAGAAGTGCCTGGCAGTGTACCAGGCCGGGGCGAATAA

>Seq13 [organism=Homo sapiens] LUAD ApoE ε2ε2

GCTAGCTAGCTAGCGGCTGGGCGCGGAATGGAGGACGTGTGCGGCCGCCTGGTGCAGTACCGCGGCGAGGTGCAGGCCATGCTCGGCCAGAGCACCGAGGAGCTGCGGGTGCGCCTCGCCTCCCACCTGCGCAAGCTGCGTAAGCGGCTCCTCCGCGATGCCGATGACCTGCAGAAGTGCCTGGCAGTGTACCAGGCCGGGGCGAATAA

>Seq14 [organism=Homo sapiens] LUAD ApoE ε2ε2

CTAGCTAGCTAGCCGGCTGGGCGCGGAATGGAGGACGTGTGCGGCCGCCTGGTGCAGTACCGCGGCGAGGTGCAGGCCATGCTCGGCCAGAGCACCGAGGAGCTGCGGGTGCGCCTCGCCTCCCACCTGCGCAAGCTGCGTAAGCGGCTCCTCCGCGATGCCGATGACCTGCAGAAGTGCCTGGCAGTGTACCAGGCCGGGGCGAATAA

>Seq15 [organism=Homo sapiens] LUAD ApoE ε2ε2

CGCGCGCGCGCGCATCGCGGCTGGGCGCGGAATGGAGGACGTGTGCGGCCGCCTGGTGCAGTACCGCGGCGAGGTGCAGGCCATGCTCGGCCAGAGCACCGAGGAGCTGCGGGTGCGCCTCGCCTCCCACCTGCGCAAGCTGCGTAAGCGGCTCCTCCGCGATGCCGATGACCTGCAGAAGTGCCTGGCAGTGTACCAGGCCGGGGCGAATAA

>Seq16 [organism=Homo sapiens] LUAD ApoE ε2ε2

GGAGCCGCGCGCGCATCGCGGCTGGGCGCGGAATGGAGGACGTGTGCGGCCGCCTGGTGCAGTACCGCGGCGAGGTGCAGGCCATGCTCGGCCAGAGCACCGAGGAGCTGCGGGTGCGCCTCGCCTCCCACCTGCGCAAGCTGCGTAAGCGGCTCCTCCGCGATGCCGATGACCTGCAGAAGTGCCTGGCAGTGTACCAGGCCGGGGCGAATAA

>Seq17 [organism=Homo sapiens] LUAD ApoE ε2ε3

TCGACGGGCCGGCGCGGAATGGAGGACGTGTGCGGCCGCCTGGTGCAGTACCGCGGCGAGGTGCAGGCCATGCTCGGCCAGAGCACCGAGGAGCTGCGGGTGCGCCTCGCCTCCCACCTGCGCAAGCTGCGTAAGCGGCTCCTCCGCGATGCCGATGACCTGCAGAAGCGCCTGGCAGTGTACCAGGCCGGGGCGAAT

>Seq18 [organism=Homo sapiens] LUAD ApoE ε2ε3

TACGCGGGCCGGCGCGGAATGGAGGACGTGTGCGGCCGCCTGGTGCAGTACCGCGGCGAGGTGCAGGCCATGCTCGGCCAGAGCACCGAGGAGCTGCGGGTGCGCCTCGCCTCCCACCTGCGCAAGCTGCGTAAGCGGCTCCTCCGCGATGCCGATGACCTGCAGAAGCGCCTGGCAGTGTACCAGGCCGGGGCGAAT

>Seq19 [organism=Homo sapiens] LUAD ApoE ε2ε3

ACGTCGGGCCGGCGCGGAATGGAGGACGTGTGCGGCCGCCTGGTGCAGTACCGCGGCGAGGTGCAGGCCATGCTCGGCCAGAGCACCGAGGAGCTGCGGGTGCGCCTCGCCTCCCACCTGCGCAAGCTGCGTAAGCGGCTCCTCCGCGATGCCGATGACCTGCAGAAGCGCCTGGCAGTGTACCAGGCCGGGGCGAAT

>Seq20 [organism=Homo sapiens] LUAD ApoE ε2ε3

AGTGCGGCCGGCGCGGAATGGAGGACGTGTGCGGCCGCCTGGTGCAGTACCGCGGCGAGGTGCAGGCCATGCTCGGCCAGAGCACCGAGGAGCTGCGGGTGCGCCTCGCCTCCCACCTGCGCAAGCTGCGTAAGCGGCTCCTCCGCGATGCCGATGACCTGCAGAAGCGCCTGGCAGTGTACCAGGCCGGGGCGAAT

>Seq21 [organism=Homo sapiens] LUAD ApoE ε2ε3

ATCGGCGGCCGGCGCGGAATGGAGGACGTGTGCGGCCGCCTGGTGCAGTACCGCGGCGAGGTGCAGGCCATGCTCGGCCAGAGCACCGAGGAGCTGCGGGTGCGCCTCGCCTCCCACCTGCGCAAGCTGCGTAAGCGGCTCCTCCGCGATGCCGATGACCTGCAGAAGCGCCTGGCAGTGTACCAGGCCGGGGCGAAT

>Seq22 [organism=Homo sapiens] LUAD ApoE ε2ε3

GTCGGGCCGGCGCGGAATGGAGGACGTGTGCGGCCGCCTGGTGCAGTACCGCGGCGAGGTGCAGGCCATGCTCGGCCAGAGCACCGAGGAGCTGCGGGTGCGCCTCGCCTCCCACCTGCGCAAGCTGCGTAAGCGGCTCCTCCGCGATGCCGATGACCTGCAGAAGCGCCTGGCAGTGTACCAGGCCGGGGCGAAT

>Seq23 [organism=Homo sapiens] LUAD ApoE ε2ε3

TCACGGGCCGGCGCGGAATGGAGGACGTGTGCGGCCGCCTGGTGCAGTACCGCGGCGAGGTGCAGGCCATGCTCGGCCAGAGCACCGAGGAGCTGCGGGTGCGCCTCGCCTCCCACCTGCGCAAGCTGCGTAAGCGGCTCCTCCGCGATGCCGATGACCTGCAGAAGCGCCTGGCAGTGTACCAGGCCGGGGCGAAT

>Seq24 [organism=Homo sapiens] LUAD ApoE ε2ε3

AGGCGGCCGGCGCGGAATGGAGGACGTGTGCGGCCGCCTGGTGCAGTACCGCGGCGAGGTGCAGGCCATGCTCGGCCAGAGCACCGAGGAGCTGCGGGTGCGCCTCGCCTCCCACCTGCGCAAGCTGCGTAAGCGGCTCCTCCGCGATGCCGATGACCTGCAGAAGCGCCTGGCAGTGTACCAGGCCGGGGCGAAT

>Seq25 [organism=Homo sapiens] LUAD ApoE ε2ε3

CGCGGCGGCCGGCGCGGAATGGAGGACGTGTGCGGCCGCCTGGTGCAGTACCGCGGCGAGGTGCAGGCCATGCTCGGCCAGAGCACCGAGGAGCTGCGGGTGCGCCTCGCCTCCCACCTGCGCAAGCTGCGTAAGCGGCTCCTCCGCGATGCCGATGACCTGCAGAAGCGCCTGGCAGTGTACCAGGCCGGGGCGAAT

>Seq26 [organism=Homo sapiens] LUAD ApoE ε2ε3

AGACGGGCCGGCGCGGAATGGAGGACGTGTGCGGCCGCCTGGTGCAGTACCGCGGCGAGGTGCAGGCCATGCTCGGCCAGAGCACCGAGGAGCTGCGGGTGCGCCTCGCCTCCCACCTGCGCAAGCTGCGTAAGCGGCTCCTCCGCGATGCCGATGACCTGCAGAAGCGCCTGGCAGTGTACCAGGCCGGGGCGAAT

>Seq27 [organism=Homo sapiens] LUAD ApoE ε2ε3

CACGCGGCCGGCGCGGAATGGAGGACGTGTGCGGCCGCCTGGTGCAGTACCGCGGCGAGGTGCAGGCCATGCTCGGCCAGAGCACCGAGGAGCTGCGGGTGCGCCTCGCCTCCCACCTGCGCAAGCTGCGTAAGCGGCTCCTCCGCGATGCCGATGACCTGCAGAAGCGCCTGGCAGTGTACCAGGCCGGGGCGAAT

>Seq28 [organism=Homo sapiens] LUAD ApoE ε2ε3

ATGGCGGCCGGCGCGGAATGGAGGACGTGTGCGGCCGCCTGGTGCAGTACCGCGGCGAGGTGCAGGCCATGCTCGGCCAGAGCACCGAGGAGCTGCGGGTGCGCCTCGCCTCCCACCTGCGCAAGCTGCGTAAGCGGCTCCTCCGCGATGCCGATGACCTGCAGAAGCGCCTGGCAGTGTACCAGGCCGGGGCGAAT

>Seq29 [organism=Homo sapiens] LUAD ApoE ε2ε3

TGGGCGGCCGGCGCGGAATGGAGGACGTGTGCGGCCGCCTGGTGCAGTACCGCGGCGAGGTGCAGGCCATGCTCGGCCAGAGCACCGAGGAGCTGCGGGTGCGCCTCGCCTCCCACCTGCGCAAGCTGCGTAAGCGGCTCCTCCGCGATGCCGATGACCTGCAGAAGCGCCTGGCAGTGTACCAGGCCGGGGCGAAT

>Seq30 [organism=Homo sapiens] LUAD ApoE ε2ε3

CCGGCGGCCGGCGCGGAATGGAGGACGTGTGCGGCCGCCTGGTGCAGTACCGCGGCGAGGTGCAGGCCATGCTCGGCCAGAGCACCGAGGAGCTGCGGGTGCGCCTCGCCTCCCACCTGCGCAAGCTGCGTAAGCGGCTCCTCCGCGATGCCGATGACCTGCAGAAGCGCCTGGCAGTGTACCAGGCCGGGGCGAAT

>Seq31 [organism=Homo sapiens] LUAD ApoE ε2ε3

ACGCGGCCGGCGCGGAATGGAGGACGTGTGCGGCCGCCTGGTGCAGTACCGCGGCGAGGTGCAGGCCATGCTCGGCCAGAGCACCGAGGAGCTGCGGGTGCGCCTCGCCTCCCACCTGCGCAAGCTGCGTAAGCGGCTCCTCCGCGATGCCGATGACCTGCAGAAGCGCCTGGCAGTGTACCAGGCCGGGGCGAAT

>Seq32 [organism=Homo sapiens] LUAD ApoE ε2ε3

GCAGCGGCCGGCGCGGAATGGAGGACGTGTGCGGCCGCCTGGTGCAGTACCGCGGCGAGGTGCAGGCCATGCTCGGCCAGAGCACCGAGGAGCTGCGGGTGCGCCTCGCCTCCCACCTGCGCAAGCTGCGTAAGCGGCTCCTCCGCGATGCCGATGACCTGCAGAAGCGCCTGGCAGTGTACCAGGCCGGGGCGAAT

>Seq33 [organism=Homo sapiens] LUAD ApoE ε2ε3

GAGCGGCCGGCGCGGAATGGAGGACGTGTGCGGCCGCCTGGTGCAGTACCGCGGCGAGGTGCAGGCCATGCTCGGCCAGAGCACCGAGGAGCTGCGGGTGCGCCTCGCCTCCCACCTGCGCAAGCTGCGTAAGCGGCTCCTCCGCGATGCCGATGACCTGCAGAAGCGCCTGGCAGTGTACCAGGCCGGGGCGAAT

>Seq34 [organism=Homo sapiens] LUAD ApoE ε2ε3

TCGCGGCCGGCGCGGAATGGAGGACGTGTGCGGCCGCCTGGTGCAGTACCGCGGCGAGGTGCAGGCCATGCTCGGCCAGAGCACCGAGGAGCTGCGGGTGCGCCTCGCCTCCCACCTGCGCAAGCTGCGTAAGCGGCTCCTCCGCGATGCCGATGACCTGCAGAAGCGCCTGGCAGTGTACCAGGCCGGGGCGAAT

>Seq35 [organism=Homo sapiens] LUAD ApoE ε2ε3

CGTGGCGGCCGGCGCGGAATGGAGGACGTGTGCGGCCGCCTGGTGCAGTACCGCGGCGAGGTGCAGGCCATGCTCGGCCAGAGCACCGAGGAGCTGCGGGTGCGCCTCGCCTCCCACCTGCGCAAGCTGCGTAAGCGGCTCCTCCGCGATGCCGATGACCTGCAGAAGCGCCTGGCAGTGTACCAGGCCGGGGCGAAT

>Seq36 [organism=Homo sapiens] LUAD ApoE ε2ε3

CGCGGCGGCCGGCGCGGAATGGAGGACGTGTGCGGCCGCCTGGTGCAGTACCGCGGCGAGGTGCAGGCCATGCTCGGCCAGAGCACCGAGGAGCTGCGGGTGCGCCTCGCCTCCCACCTGCGCAAGCTGCGTAAGCGGCTCCTCCGCGATGCCGATGACCTGCAGAAGCGCCTGGCAGTGTACCAGGCCGGGGCGAAT

>Seq37 [organism=Homo sapiens] LUAD ApoE ε2ε3

GCTCGGCCGGCGCGGAATGGAGGACGTGTGCGGCCGCCTGGTGCAGTACCGCGGCGAGGTGCAGGCCATGCTCGGCCAGAGCACCGAGGAGCTGCGGGTGCGCCTCGCCTCCCACCTGCGCAAGCTGCGTAAGCGGCTCCTCCGCGATGCCGATGACCTGCAGAAGCGCCTGGCAGTGTACCAGGCCGGGGCGAAT

>Seq38 [organism=Homo sapiens] LUAD ApoE ε2ε3

TACGGCGGCCGGCGCGGAATGGAGGACGTGTGCGGCCGCCTGGTGCAGTACCGCGGCGAGGTGCAGGCCATGCTCGGCCAGAGCACCGAGGAGCTGCGGGTGCGCCTCGCCTCCCACCTGCGCAAGCTGCGTAAGCGGCTCCTCCGCGATGCCGATGACCTGCAGAAGCGCCTGGCAGTGTACCAGGCCGGGGCGAAT

>Seq39 [organism=Homo sapiens] LUAD ApoE ε2ε3

ACGGCGGCCGGCGCGGAATGGAGGACGTGTGCGGCCGCCTGGTGCAGTACCGCGGCGAGGTGCAGGCCATGCTCGGCCAGAGCACCGAGGAGCTGCGGGTGCGCCTCGCCTCCCACCTGCGCAAGCTGCGTAAGCGGCTCCTCCGCGATGCCGATGACCTGCAGAAGCGCCTGGCAGTGTACCAGGCCGGGGCGAAT

>Seq40 [organism=Homo sapiens] LUAD ApoE ε2ε3

GCGGCGGCCGGCGCGGAATGGAGGACGTGTGCGGCCGCCTGGTGCAGTACCGCGGCGAGGTGCAGGCCATGCTCGGCCAGAGCACCGAGGAGCTGCGGGTGCGCCTCGCCTCCCACCTGCGCAAGCTGCGTAAGCGGCTCCTCCGCGATGCCGATGACCTGCAGAAGCGCCTGGCAGTGTACCAGGCCGGGGCGAAT

>Seq41 [organism=Homo sapiens] LUAD ApoE ε2ε3

GACCGGCCGGCGCGGAATGGAGGACGTGTGCGGCCGCCTGGTGCAGTACCGCGGCGAGGTGCAGGCCATGCTCGGCCAGAGCACCGAGGAGCTGCGGGTGCGCCTCGCCTCCCACCTGCGCAAGCTGCGTAAGCGGCTCCTCCGCGATGCCGATGACCTGCAGAAGCGCCTGGCAGTGTACCAGGCCGGGGCGAAT

>Seq42 [organism=Homo sapiens] LUAD ApoE ε3ε3

CACGTAGCGGGGCGCGGAATGGAGGACGTGTGCGGCCGCCTGGTGCAGTACCGCGGCGAGGTGCAGGCCATGCTCGGCCAGAGCACCGAGGAGCTGCGGGTGCGCCTCGCCTCCCACCTGCGCAAGCTGCGTAAGCGGCTCCTCCGCGATGCCGATGACCTGCAGAAGCGCCTGGCAGTGTACCAGGCCGGGGCGAAT

>Seq43 [organism=Homo sapiens] LUAD ApoE ε3ε3

CGAGACGAGGCGCATGTCGCTGTGGGCGCGGAATGGCAGGACGTGTGCGGCCGCCTGGTGCAGTACCGGGCGCGGAATGGAGGACGTGTGCGGCCGCCTGGTGCAGTACCGCGGCGAGGTGCAGGCCATGCTCGGCCAGAGCACCGAGGAGCTGCGGGTGCGCCTCGCCTCCCACCTGCGCAAGCTGCGTAAGCGGCTCCTCCGCGATGCCGATGACCTGCAGAAGCGCCTGGCAGTGTACCAGGCCGGGGCGAAT

>Seq44 [organism=Homo sapiens] LUAD ApoE ε3ε3

GCCGTCGAGTGCATGTCGCTGTGGGCGCGGAATGGCAGGACGTGTGCGGCCGCCTGGTGCAGTACCGGGCGCGGAATGGAGGACGTGTGCGGCCGCCTGGTGCAGTACCGCGGCGAGGTGCAGGCCATGCTCGGCCAGAGCACCGAGGAGCTGCGGGTGCGCCTCGCCTCCCACCTGCGCAAGCTGCGTAAGCGGCTCCTCCGCGATGCCGATGACCTGCAGAAGCGCCTGGCAGTGTACCAGGCCGGGGCGAAT

>Seq45 [organism=Homo sapiens] LUAD ApoE ε3ε3

GACGCGAGCTGCATGTCGCTGTGGGCGCGGAATGGCAGGACGTGTGCGGCCGCCTGGTGCAGTACCGGGCGCGGAATGGAGGACGTGTGCGGCCGCCTGGTGCAGTACCGCGGCGAGGTGCAGGCCATGCTCGGCCAGAGCACCGAGGAGCTGCGGGTGCGCCTCGCCTCCCACCTGCGCAAGCTGCGTAAGCGGCTCCTCCGCGATGCCGATGACCTGCAGAAGCGCCTGGCAGTGTACCAGGCCGGGGCGAAT

>Seq46 [organism=Homo sapiens] LUAD ApoE ε3ε3

GGAGCGAGGCGCATGTCGCTGTGGGCGCGGAATGGCAGGACGTGTGCGGCCGCCTGGTGCAGTACCGGGCGCGGAATGGAGGACGTGTGCGGCCGCCTGGTGCAGTACCGCGGCGAGGTGCAGGCCATGCTCGGCCAGAGCACCGAGGAGCTGCGGGTGCGCCTCGCCTCCCACCTGCGCAAGCTGCGTAAGCGGCTCCTCCGCGATGCCGATGACCTGCAGAAGCGCCTGGCAGTGTACCAGGCCGGGGCGAAT

>Seq47 [organism=Homo sapiens] LUAD ApoE ε3ε3

GTGACGAGTGCATGTCGCTGTGGGCGCGGAATGGCAGGACGTGTGCGGCCGCCTGGTGCAGTACCGGGCGCGGAATGGAGGACGTGTGCGGCCGCCTGGTGCAGTACCGCGGCGAGGTGCAGGCCATGCTCGGCCAGAGCACCGAGGAGCTGCGGGTGCGCCTCGCCTCCCACCTGCGCAAGCTGCGTAAGCGGCTCCTCCGCGATGCCGATGACCTGCAGAAGCGCCTGGCAGTGTACCAGGCCGGGGCGAAT

>Seq48 [organism=Homo sapiens] LUAD ApoE ε3ε3

GAGGCGAGGCGCATGTCGCTGTGGGCGCGGAATGGCAGGACGTGTGCGGCCGCCTGGTGCAGTACCGGGCGCGGAATGGAGGACGTGTGCGGCCGCCTGGTGCAGTACCGCGGCGAGGTGCAGGCCATGCTCGGCCAGAGCACCGAGGAGCTGCGGGTGCGCCTCGCCTCCCACCTGCGCAAGCTGCGTAAGCGGCTCCTCCGCGATGCCGATGACCTGCAGAAGCGCCTGGCAGTGTACCAGGCCGGGGCGAAT

>Seq49 [organism=Homo sapiens] LUAD ApoE ε3ε3

GTGTCGAGTGCATGTCGCTGTGGGCGCGGAATGGCAGGACGTGTGCGGCCGCCTGGTGCAGTACCGGGCGCGGAATGGAGGACGTGTGCGGCCGCCTGGTGCAGTACCGCGGCGAGGTGCAGGCCATGCTCGGCCAGAGCACCGAGGAGCTGCGGGTGCGCCTCGCCTCCCACCTGCGCAAGCTGCGTAAGCGGCTCCTCCGCGATGCCGATGACCTGCAGAAGCGCCTGGCAGTGTACCAGGCCGGGGCGAAT

>Seq50 [organism=Homo sapiens] LUAD ApoE ε3ε3

GCTACGAGCTGCATGTCGCTGTGGGCGCGGAATGGCAGGACGTGTGCGGCCGCCTGGTGCAGTACCGGGCGCGGAATGGAGGACGTGTGCGGCCGCCTGGTGCAGTACCGCGGCGAGGTGCAGGCCATGCTCGGCCAGAGCACCGAGGAGCTGCGGGTGCGCCTCGCCTCCCACCTGCGCAAGCTGCGTAAGCGGCTCCTCCGCGATGCCGATGACCTGCAGAAGCGCCTGGCAGTGTACCAGGCCGGGGCGAAT

>Seq51 [organism=Homo sapiens] LUAD ApoE ε3ε3

CGACCGAGGCGCATGTCGCTGTGGGCGCGGAATGGCAGGACGTGTGCGGCCGCCTGGTGCAGTACCGGGCGCGGAATGGAGGACGTGTGCGGCCGCCTGGTGCAGTACCGCGGCGAGGTGCAGGCCATGCTCGGCCAGAGCACCGAGGAGCTGCGGGTGCGCCTCGCCTCCCACCTGCGCAAGCTGCGTAAGCGGCTCCTCCGCGATGCCGATGACCTGCAGAAGCGCCTGGCAGTGTACCAGGCCGGGGCGAAT

>Seq52 [organism=Homo sapiens] LUAD ApoE ε3ε3

GTAGCGAGTGCATGTCGCTGTGGGCGCGGAATGGCAGGACGTGTGCGGCCGCCTGGTGCAGTACCGGGCGCGGAATGGAGGACGTGTGCGGCCGCCTGGTGCAGTACCGCGGCGAGGTGCAGGCCATGCTCGGCCAGAGCACCGAGGAGCTGCGGGTGCGCCTCGCCTCCCACCTGCGCAAGCTGCGTAAGCGGCTCCTCCGCGATGCCGATGACCTGCAGAAGCGCCTGGCAGTGTACCAGGCCGGGGCGAAT

>Seq53 [organism=Homo sapiens] LUAD ApoE ε3ε3

GCGAGGAGGCGCATGTCGCTGTGGGCGCGGAATGGCAGGACGTGTGCGGCCGCCTGGTGCAGTACCGGGCGCGGAATGGAGGACGTGTGCGGCCGCCTGGTGCAGTACCGCGGCGAGGTGCAGGCCATGCTCGGCCAGAGCACCGAGGAGCTGCGGGTGCGCCTCGCCTCCCACCTGCGCAAGCTGCGTAAGCGGCTCCTCCGCGATGCCGATGACCTGCAGAAGCGCCTGGCAGTGTACCAGGCCGGGGCGAAT >Seq54 [organism=Homo sapiens] LUAD ApoE ε3ε3

GACAGGAGGCGCATGTCGCTGTGGGCGCGGAATGGCAGGACGTGTGCGGCCGCCTGGTGCAGTACCGGGCGCGGAATGGAGGACGTGTGCGGCCGCCTGGTGCAGTACCGCGGCGAGGTGCAGGCCATGCTCGGCCAGAGCACCGAGGAGCTGCGGGTGCGCCTCGCCTCCCACCTGCGCAAGCTGCGTAAGCGGCTCCTCCGCGATGCCGATGACCTGCAGAAGCGCCTGGCAGTGTACCAGGCCGGGGCGAAT

>Seq55 [organism=Homo sapiens] LUAD ApoE ε3ε3

GCTCGGAGGCGCATGTCGCTGTGGGCGCGGAATGGCAGGACGTGTGCGGCCGCCTGGTGCAGTACCGGGCGCGGAATGGAGGACGTGTGCGGCCGCCTGGTGCAGTACCGCGGCGAGGTGCAGGCCATGCTCGGCCAGAGCACCGAGGAGCTGCGGGTGCGCCTCGCCTCCCACCTGCGCAAGCTGCGTAAGCGGCTCCTCCGCGATGCCGATGACCTGCAGAAGCGCCTGGCAGTGTACCAGGCCGGGGCGAAT

>Seq56 [organism=Homo sapiens] LUAD ApoE ε3ε3

GCAGGGAGGCGCATGTCGCTGTGGGCGCGGAATGGCAGGACGTGTGCGGCCGCCTGGTGCAGTACCGGGCGCGGAATGGAGGACGTGTGCGGCCGCCTGGTGCAGTACCGCGGCGAGGTGCAGGCCATGCTCGGCCAGAGCACCGAGGAGCTGCGGGTGCGCCTCGCCTCCCACCTGCGCAAGCTGCGTAAGCGGCTCCTCCGCGATGCCGATGACCTGCAGAAGCGCCTGGCAGTGTACCAGGCCGGGGCGAAT

>Seq57 [organism=Homo sapiens] LUAD ApoE ε3ε3

GAGTGGAGGCGCATGTCGCTGTGGGCGCGGAATGGCAGGACGTGTGCGGCCGCCTGGTGCAGTACCGGGCGCGGAATGGAGGACGTGTGCGGCCGCCTGGTGCAGTACCGCGGCGAGGTGCAGGCCATGCTCGGCCAGAGCACCGAGGAGCTGCGGGTGCGCCTCGCCTCCCACCTGCGCAAGCTGCGTAAGCGGCTCCTCCGCGATGCCGATGACCTGCAGAAGCGCCTGGCAGTGTACCAGGCCGGGGCGAAT

>Seq58 [organism=Homo sapiens] LUAD ApoE ε3ε3

GCGTCGAGGCGCATGTCGCTGTGGGCGCGGAATGGCAGGACGTGTGCGGCCGCCTGGTGCAGTACCGGGCGCGGAATGGAGGACGTGTGCGGCCGCCTGGTGCAGTACCGCGGCGAGGTGCAGGCCATGCTCGGCCAGAGCACCGAGGAGCTGCGGGTGCGCCTCGCCTCCCACCTGCGCAAGCTGCGTAAGCGGCTCCTCCGCGATGCCGATGACCTGCAGAAGCGCCTGGCAGTGTACCAGGCCGGGGCGAAT

>Seq59 [organism=Homo sapiens] LUAD ApoE ε3ε3

GGTAGGAGGCGCATGTCGCTGTGGGCGCGGAATGGCAGGACGTGTGCGGCCGCCTGGTGCAGTACCGGGCGCGGAATGGAGGACGTGTGCGGCCGCCTGGTGCAGTACCGCGGCGAGGTGCAGGCCATGCTCGGCCAGAGCACCGAGGAGCTGCGGGTGCGCCTCGCCTCCCACCTGCGCAAGCTGCGTAAGCGGCTCCTCCGCGATGCCGATGACCTGCAGAAGCGCCTGGCAGTGTACCAGGCCGGGGCGAAT

>Seq60 [organism=Homo sapiens] LUAD ApoE ε3ε3

GGCGAGAGGCGCATGTCGCTGTGGGCGCGGAATGGCAGGACGTGTGCGGCCGCCTGGTGCAGTACCGGGCGCGGAATGGAGGACGTGTGCGGCCGCCTGGTGCAGTACCGCGGCGAGGTGCAGGCCATGCTCGGCCAGAGCACCGAGGAGCTGCGGGTGCGCCTCGCCTCCCACCTGCGCAAGCTGCGTAAGCGGCTCCTCCGCGATGCCGATGACCTGCAGAAGCGCCTGGCAGTGTACCAGGCCGGGGCGAAT

>Seq61 [organism=Homo sapiens] LUAD ApoE ε3ε3

GTAGCGAGGCGCATGTCGCTGTGGGCGCGGAATGGCAGGACGTGTGCGGCCGCCTGGTGCAGTACCGCGGCGAGGTGCAGGCCATGCTCGGCCAGAGCACCGATGAGCTGCGGGTGCGCCTCGCCTCCCACCTGCGCAAGCTGCGTAAGCGGCTCCTCCGCGATGCCGATGACCTGCAGAAGCGCCTGGCAGTGTACC

>Seq62 [organism=Homo sapiens] LUAD ApoE ε3ε3

GACGGCGTAGCGAGGCGCATGTCGCTGTGGGCGCGGAATGGCAGGACGTGTGCGGCCGCCTGGTGCAGTACCGCGGCGAGGTGCAGGCCATGCTCGGCCAGAGCACCGATGAGCTGCGGGTGCGCCTCGCCTCCCACCTGCGCAAGCTGCGTAAGCGGCTCCTCCGCGATGCCGATGACCTGCAGAAGCGCCTGGCAAT

>Seq63 [organism=Homo sapiens] LUAD ApoE ε3ε3

GCGGGGAGGCGCATGTCGCTGTGGGCGCGGAATGGCAGGACGTGTGCGGCCGCCTGGTGCAGTACCGGGCGCGGAATGGAGGACGTGTGCGGCCGCCTGGTGCAGTACCGCGGCGAGGTGCAGGCCATGCTCGGCCAGAGCACCGAGGAGCTGCGGGTGCGCCTCGCCTCCCACCTGCGCAAGCTGCGTAAGCGGCTCCTCCGCGATGCCGATGACCTGCAGAAGCGCCTGGCAGTGTACCAGGCCGGGGCGAAT

>Seq64 [organism=Homo sapiens] LUAD ApoE ε3ε3

GTCAGGAGGCGCATGTCGCTGTGGGCGCGGAATGGCAGGACGTGTGCGGCCGCCTGGTGCAGTACCGGGCGCGGAATGGAGGACGTGTGCGGCCGCCTGGTGCAGTACCGCGGCGAGGTGCAGGCCATGCTCGGCCAGAGCACCGAGGAGCTGCGGGTGCGCCTCGCCTCCCACCTGCGCAAGCTGCGTAAGCGGCTCCTCCGCGATGCCGATGACCTGCAGAAGCGCCTGGCAGTGTACCAGGCCGGGGCGAAT

>Seq65 [organism=Homo sapiens] LUAD ApoE ε3ε3

GCGTCGAGGCGCATGTCGCTGTGGGCGCGGAATGGCAGGACGTGTGCGGCCGCCTGGTGCAGTACCGGGCGCGGAATGGAGGACGTGTGCGGCCGCCTGGTGCAGTACCGCGGCGAGGTGCAGGCCATGCTCGGCCAGAGCACCGAGGAGCTGCGGGTGCGCCTCGCCTCCCACCTGCGCAAGCTGCGTAAGCGGCTCCTCCGCGATGCCGATGACCTGCAGAAGCGCCTGGCAGTGTACCAGGCCGGGGCGAAT

>Seq66 [organism=Homo sapiens] LUAD ApoE ε3ε3

GACGGGAGGCGCATGTCGCTGTGGGCGCGGAATGGCAGGACGTGTGCGGCCGCCTGGTGCAGTACCGGGCGCGGAATGGAGGACGTGTGCGGCCGCCTGGTGCAGTACCGCGGCGAGGTGCAGGCCATGCTCGGCCAGAGCACCGAGGAGCTGCGGGTGCGCCTCGCCTCCCACCTGCGCAAGCTGCGTAAGCGGCTCCTCCGCGATGCCGATGACCTGCAGAAGCGCCTGGCAGTGTACCAGGCCGGGGCGAAT

>Seq67 [organism=Homo sapiens] LUAD ApoE ε3ε3

GACAGGAGGCGCATGTCGCTGTGGGCGCGGAATGGCAGGACGTGTGCGGCCGCCTGGTGCAGTACCGGGCGCGGAATGGAGGACGTGTGCGGCCGCCTGGTGCAGTACCGCGGCGAGGTGCAGGCCATGCTCGGCCAGAGCACCGAGGAGCTGCGGGTGCGCCTCGCCTCCCACCTGCGCAAGCTGCGTAAGCGGCTCCTCCGCGATGCCGATGACCTGCAGAAGCGCCTGGCAGTGTACCAGGCCGGGGCGAAT

>Seq68 [organism=Homo sapiens] LUAD ApoE ε3ε3

GTCGGGAGGCGCATGTCGCTGTGGGCGCGGAATGGCAGGACGTGTGCGGCCGCCTGGTGCAGTACCGGGCGCGGAATGGAGGACGTGTGCGGCCGCCTGGTGCAGTACCGCGGCGAGGTGCAGGCCATGCTCGGCCAGAGCACCGAGGAGCTGCGGGTGCGCCTCGCCTCCCACCTGCGCAAGCTGCGTAAGCGGCTCCTCCGCGATGCCGATGACCTGCAGAAGCGCCTGGCAGTGTACCAGGCCGGGGCGAAT

>Seq69 [organism=Homo sapiens] LUAD ApoE ε3ε3

GAGCGGAGGCGCATGTCGCTGTGGGCGCGGAATGGCAGGACGTGTGCGGCCGCCTGGTGCAGTACCGGGCGCGGAATGGAGGACGTGTGCGGCCGCCTGGTGCAGTACCGCGGCGAGGTGCAGGCCATGCTCGGCCAGAGCACCGAGGAGCTGCGGGTGCGCCTCGCCTCCCACCTGCGCAAGCTGCGTAAGCGGCTCCTCCGCGATGCCGATGACCTGCAGAAGCGCCTGGCAGTGTACCAGGCCGGGGCGAAT

>Seq70 [organism=Homo sapiens] LUAD ApoE ε3ε3

GGTCGGAGGCGCATGTCGCTGTGGGCGCGGAATGGCAGGACGTGTGCGGCCGCCTGGTGCAGTACCGGGCGCGGAATGGAGGACGTGTGCGGCCGCCTGGTGCAGTACCGCGGCGAGGTGCAGGCCATGCTCGGCCAGAGCACCGAGGAGCTGCGGGTGCGCCTCGCCTCCCACCTGCGCAAGCTGCGTAAGCGGCTCCTCCGCGATGCCGATGACCTGCAGAAGCGCCTGGCAGTGTACCAGGCCGGGGCGAAT

>Seq71 [organism=Homo sapiens] LUAD ApoE ε3ε3

GGTGGGAGGCGCATGTCGCTGTGGGCGCGGAATGGCAGGACGTGTGCGGCCGCCTGGTGCAGTACCGGGCGCGGAATGGAGGACGTGTGCGGCCGCCTGGTGCAGTACCGCGGCGAGGTGCAGGCCATGCTCGGCCAGAGCACCGAGGAGCTGCGGGTGCGCCTCGCCTCCCACCTGCGCAAGCTGCGTAAGCGGCTCCTCCGCGATGCCGATGACCTGCAGAAGCGCCTGGCAGTGTACCAGGCCGGGGCGAAT

>Seq72 [organism=Homo sapiens] LUAD ApoE ε3ε3

GCTGGGAGGCGCATGTCGCTGTGGGCGCGGAATGGCAGGACGTGTGCGGCCGCCTGGTGCAGTACCGGGCGCGGAATGGAGGACGTGTGCGGCCGCCTGGTGCAGTACCGCGGCGAGGTGCAGGCCATGCTCGGCCAGAGCACCGAGGAGCTGCGGGTGCGCCTCGCCTCCCACCTGCGCAAGCTGCGTAAGCGGCTCCTCCGCGATGCCGATGACCTGCAGAAGCGCCTGGCAGTGTACCAGGCCGGGGCGAAT

>Seq73 [organism=Homo sapiens] LUAD ApoE ε3ε3

GCTGTGAGGCGCATGTCGCTGTGGGCGCGGAATGGCAGGACGTGTGCGGCCGCCTGGTGCAGTACCGGGCGCGGAATGGAGGACGTGTGCGGCCGCCTGGTGCAGTACCGCGGCGAGGTGCAGGCCATGCTCGGCCAGAGCACCGAGGAGCTGCGGGTGCGCCTCGCCTCCCACCTGCGCAAGCTGCGTAAGCGGCTCCTCCGCGATGCCGATGACCTGCAGAAGCGCCTGGCAGTGTACCAGGCCGGGGCGAAT

>Seq74 [organism=Homo sapiens] LUAD ApoE ε3ε3

GAGTGGAGGCGCATGTCGCTGTGGGCGCGGAATGGCAGGACGTGTGCGGCCGCCTGGTGCAGTACCGGGCGCGGAATGGAGGACGTGTGCGGCCGCCTGGTGCAGTACCGCGGCGAGGTGCAGGCCATGCTCGGCCAGAGCACCGAGGAGCTGCGGGTGCGCCTCGCCTCCCACCTGCGCAAGCTGCGTAAGCGGCTCCTCCGCGATGCCGATGACCTGCAGAAGCGCCTGGCAGTGTACCAGGCCGGGGCGAAT

>Seq75 [organism=Homo sapiens] LUAD ApoE ε3ε3

GCGTGGAGGCGCATGTCGCTGTGGGCGCGGAATGGCAGGACGTGTGCGGCCGCCTGGTGCAGTACCGGGCGCGGAATGGAGGACGTGTGCGGCCGCCTGGTGCAGTACCGCGGCGAGGTGCAGGCCATGCTCGGCCAGAGCACCGAGGAGCTGCGGGTGCGCCTCGCCTCCCACCTGCGCAAGCTGCGTAAGCGGCTCCTCCGCGATGCCGATGACCTGCAGAAGCGCCTGGCAGTGTACCAGGCCGGGGCGAAT

>Seq76 [organism=Homo sapiens] LUAD ApoE ε3ε3

GCAGGGAGGCGCATGTCGCTGTGGGCGCGGAATGGCAGGACGTGTGCGGCCGCCTGGTGCAGTACCGGGCGCGGAATGGAGGACGTGTGCGGCCGCCTGGTGCAGTACCGCGGCGAGGTGCAGGCCATGCTCGGCCAGAGCACCGAGGAGCTGCGGGTGCGCCTCGCCTCCCACCTGCGCAAGCTGCGTAAGCGGCTCCTCCGCGATGCCGATGACCTGCAGAAGCGCCTGGCAGTGTACCAGGCCGGGGCGAAT

>Seq77 [organism=Homo sapiens] LUAD ApoE ε3ε3

GGTCGGAGGCGCATGTCGCTGTGGGCGCGGAATGGCAGGACGTGTGCGGCCGCCTGGTGCAGTACCGGGCGCGGAATGGAGGACGTGTGCGGCCGCCTGGTGCAGTACCGCGGCGAGGTGCAGGCCATGCTCGGCCAGAGCACCGAGGAGCTGCGGGTGCGCCTCGCCTCCCACCTGCGCAAGCTGCGTAAGCGGCTCCTCCGCGATGCCGATGACCTGCAGAAGCGCCTGGCAGTGTACCAGGCCGGGGCGAAT

>Seq78 [organism=Homo sapiens] LUAD ApoE ε3ε3

GGCGGGAGGCGCATGTCGCTGTGGGCGCGGAATGGCAGGACGTGTGCGGCCGCCTGGTGCAGTACCGGGCGCGGAATGGAGGACGTGTGCGGCCGCCTGGTGCAGTACCGCGGCGAGGTGCAGGCCATGCTCGGCCAGAGCACCGAGGAGCTGCGGGTGCGCCTCGCCTCCCACCTGCGCAAGCTGCGTAAGCGGCTCCTCCGCGATGCCGATGACCTGCAGAAGCGCCTGGCAGTGTACCAGGCCGGGGCGAAT

>Seq79 [organism=Homo sapiens] LUAD ApoE ε3ε3

GAGCGGAGGCGCATGTCGCTGTGGGCGCGGAATGGCAGGACGTGTGCGGCCGCCTGGTGCAGTACCGGGCGCGGAATGGAGGACGTGTGCGGCCGCCTGGTGCAGTACCGCGGCGAGGTGCAGGCCATGCTCGGCCAGAGCACCGAGGAGCTGCGGGTGCGCCTCGCCTCCCACCTGCGCAAGCTGCGTAAGCGGCTCCTCCGCGATGCCGATGACCTGCAGAAGCGCCTGGCAGTGTACCAGGCCGGGGCGAAT

>Seq80 [organism=Homo sapiens] LUAD ApoE ε3ε3

GTCGAGAGGCGCATGTCGCTGTGGGCGCGGAATGGCAGGACGTGTGCGGCCGCCTGGTGCAGTACCGGGCGCGGAATGGAGGACGTGTGCGGCCGCCTGGTGCAGTACCGCGGCGAGGTGCAGGCCATGCTCGGCCAGAGCACCGAGGAGCTGCGGGTGCGCCTCGCCTCCCACCTGCGCAAGCTGCGTAAGCGGCTCCTCCGCGATGCCGATGACCTGCAGAAGCGCCTGGCAGTGTACCAGGCCGGGGCGAAT

>Seq81 [organism=Homo sapiens] LUAD ApoE ε3ε3

GTCTGGAGGCGCATGTCGCTGTGGGCGCGGAATGGCAGGACGTGTGCGGCCGCCTGGTGCAGTACCGGGCGCGGAATGGAGGACGTGTGCGGCCGCCTGGTGCAGTACCGCGGCGAGGTGCAGGCCATGCTCGGCCAGAGCACCGAGGAGCTGCGGGTGCGCCTCGCCTCCCACCTGCGCAAGCTGCGTAAGCGGCTCCTCCGCGATGCCGATGACCTGCAGAAGCGCCTGGCAGTGTACCAGGCCGGGGCGAAT

>Seq82 [organism=Homo sapiens] LUAD ApoE ε3ε3

GTCCGGAGGCGCATGTCGCTGTGGGCGCGGAATGGCAGGACGTGTGCGGCCGCCTGGTGCAGTACCGGGCGCGGAATGGAGGACGTGTGCGGCCGCCTGGTGCAGTACCGCGGCGAGGTGCAGGCCATGCTCGGCCAGAGCACCGAGGAGCTGCGGGTGCGCCTCGCCTCCCACCTGCGCAAGCTGCGTAAGCGGCTCCTCCGCGATGCCGATGACCTGCAGAAGCGCCTGGCAGTGTACCAGGCCGGGGCGAAT

>Seq83 [organism=Homo sapiens] LUAD ApoE ε3ε3

GTCAGGAGGCGCATGTCGCTGTGGGCGCGGAATGGCAGGACGTGTGCGGCCGCCTGGTGCAGTACCGGGCGCGGAATGGAGGACGTGTGCGGCCGCCTGGTGCAGTACCGCGGCGAGGTGCAGGCCATGCTCGGCCAGAGCACCGAGGAGCTGCGGGTGCGCCTCGCCTCCCACCTGCGCAAGCTGCGTAAGCGGCTCCTCCGCGATGCCGATGACCTGCAGAAGCGCCTGGCAGTGTACCAGGCCGGGGCGAAT

>Seq84 [organism=Homo sapiens] LUAD ApoE ε3ε3

GCCGGGAGGCGCATGTCGCTGTGGGCGCGGAATGGCAGGACGTGTGCGGCCGCCTGGTGCAGTACCGGGCGCGGAATGGAGGACGTGTGCGGCCGCCTGGTGCAGTACCGCGGCGAGGTGCAGGCCATGCTCGGCCAGAGCACCGAGGAGCTGCGGGTGCGCCTCGCCTCCCACCTGCGCAAGCTGCGTAAGCGGCTCCTCCGCGATGCCGATGACCTGCAGAAGCGCCTGGCAGTGTACCAGGCCGGGGCGAAT

>Seq85 [organism=Homo sapiens] LUAD ApoE ε3ε3

GTCGGAAGGCGCATGTCGCTGTGGGCGCGGAATGGCAGGACGTGTGCGGCCGCCTGGTGCAGTACCGGGCGCGGAATGGAGGACGTGTGCGGCCGCCTGGTGCAGTACCGCGGCGAGGTGCAGGCCATGCTCGGCCAGAGCACCGAGGAGCTGCGGGTGCGCCTCGCCTCCCACCTGCGCAAGCTGCGTAAGCGGCTCCTCCGCGATGCCGATGACCTGCAGAAGCGCCTGGCAGTGTACCAGGCCGGGGCGAAT

>Seq86 [organism=Homo sapiens] LUAD ApoE ε3ε3

GTCGGGTGGCGCATGTCGCTGTGGGCGCGGAATGGCAGGACGTGTGCGGCCGCCTGGTGCAGTACCGGGCGCGGAATGGAGGACGTGTGCGGCCGCCTGGTGCAGTACCGCGGCGAGGTGCAGGCCATGCTCGGCCAGAGCACCGAGGAGCTGCGGGTGCGCCTCGCCTCCCACCTGCGCAAGCTGCGTAAGCGGCTCCTCCGCGATGCCGATGACCTGCAGAAGCGCCTGGCAGTGTACCAGGCCGGGGCGAAT

>Seq87 [organism=Homo sapiens] LUAD ApoE ε3ε3

GTCGGGATGCGCATGTCGCTGTGGGCGCGGAATGGCAGGACGTGTGCGGCCGCCTGGTGCAGTACCGGGCGCGGAATGGAGGACGTGTGCGGCCGCCTGGTGCAGTACCGCGGCGAGGTGCAGGCCATGCTCGGCCAGAGCACCGAGGAGCTGCGGGTGCGCCTCGCCTCCCACCTGCGCAAGCTGCGTAAGCGGCTCCTCCGCGATGCCGATGACCTGCAGAAGCGCCTGGCAGTGTACCAGGCCGGGGCGAAT

>Seq88 [organism=Homo sapiens] LUAD ApoE ε3ε3

GTCGGTAGGCGCATGTCGCTGTGGGCGCGGAATGGCAGGACGTGTGCGGCCGCCTGGTGCAGTACCGGGCGCGGAATGGAGGACGTGTGCGGCCGCCTGGTGCAGTACCGCGGCGAGGTGCAGGCCATGCTCGGCCAGAGCACCGAGGAGCTGCGGGTGCGCCTCGCCTCCCACCTGCGCAAGCTGCGTAAGCGGCTCCTCCGCGATGCCGATGACCTGCAGAAGCGCCTGGCAGTGTACCAGGCCGGGGCGAAT

>Seq89 [organism=Homo sapiens] LUAD ApoE ε3ε3

GGCGGGAGGCGCATGTCGCTGTGGGCGCGGAATGGCAGGACGTGTGCGGCCGCCTGGTGCAGTACCGGGCGCGGAATGGAGGACGTGTGCGGCCGCCTGGTGCAGTACCGCGGCGAGGTGCAGGCCATGCTCGGCCAGAGCACCGAGGAGCTGCGGGTGCGCCTCGCCTCCCACCTGCGCAAGCTGCGTAAGCGGCTCCTCCGCGATGCCGATGACCTGCAGAAGCGCCTGGCAGTGTACCAGGCCGGGGCGAAT

>Seq90 [organism=Homo sapiens] LUAD ApoE ε3ε3

GCCGGGAGGCGCATGTCGCTGTGGGCGCGGAATGGCAGGACGTGTGCGGCCGCCTGGTGCAGTACCGGGCGCGGAATGGAGGACGTGTGCGGCCGCCTGGTGCAGTACCGCGGCGAGGTGCAGGCCATGCTCGGCCAGAGCACCGAGGAGCTGCGGGTGCGCCTCGCCTCCCACCTGCGCAAGCTGCGTAAGCGGCTCCTCCGCGATGCCGATGACCTGCAGAAGCGCCTGGCAGTGTACCAGGCCGGGGCGAAT

>Seq91 [organism=Homo sapiens] LUAD ApoE ε3ε3

GTCGGGCGGCGCATGTCGCTGTGGGCGCGGAATGGCAGGACGTGTGCGGCCGCCTGGTGCAGTACCGGGCGCGGAATGGAGGACGTGTGCGGCCGCCTGGTGCAGTACCGCGGCGAGGTGCAGGCCATGCTCGGCCAGAGCACCGAGGAGCTGCGGGTGCGCCTCGCCTCCCACCTGCGCAAGCTGCGTAAGCGGCTCCTCCGCGATGCCGATGACCTGCAGAAGCGCCTGGCAGTGTACCAGGCCGGGGCGAAT

>Seq92 [organism=Homo sapiens] LUAD ApoE ε3ε3

GTCGGCAGGCGCATGTCGCTGTGGGCGCGGAATGGCAGGACGTGTGCGGCCGCCTGGTGCAGTACCGGGCGCGGAATGGAGGACGTGTGCGGCCGCCTGGTGCAGTACCGCGGCGAGGTGCAGGCCATGCTCGGCCAGAGCACCGAGGAGCTGCGGGTGCGCCTCGCCTCCCACCTGCGCAAGCTGCGTAAGCGGCTCCTCCGCGATGCCGATGACCTGCAGAAGCGCCTGGCAGTGTACCAGGCCGGGGCGAAT

>Seq93 [organism=Homo sapiens] LUAD ApoE ε3ε3

GTCGGCAGGCGCATGTCGCTGTGGGCGCGGAATGGCAGGACGTGTGCGGCCGCCTGGTGCAGTACCGGGCGCGGAATGGAGGACGTGTGCGGCCGCCTGGTGCAGTACCGCGGCGAGGTGCAGGCCATGCTCGGCCAGAGCACCGAGGAGCTGCGGGTGCGCCTCGCCTCCCACCTGCGCAAGCTGCGTAAGCGGCTCCTCCGCGATGCCGATGACCTGCAGAAGCGCCTGGCAGTGTACCAGGCCGGGGCGAAT

>Seq94 [organism=Homo sapiens] LUAD ApoE ε3ε3

GTCGTGAGGCGCATGTCGCTGTGGGCGCGGAATGGCAGGACGTGTGCGGCCGCCTGGTGCAGTACCGGGCGCGGAATGGAGGACGTGTGCGGCCGCCTGGTGCAGTACCGCGGCGAGGTGCAGGCCATGCTCGGCCAGAGCACCGAGGAGCTGCGGGTGCGCCTCGCCTCCCACCTGCGCAAGCTGCGTAAGCGGCTCCTCCGCGATGCCGATGACCTGCAGAAGCGCCTGGCAGTGTACCAGGCCGGGGCGAAT

>Seq95 [organism=Homo sapiens] LUAD ApoE ε3ε3

GTCGAGAGGCGCATGTCGCTGTGGGCGCGGAATGGCAGGACGTGTGCGGCCGCCTGGTGCAGTACCGGGCGCGGAATGGAGGACGTGTGCGGCCGCCTGGTGCAGTACCGCGGCGAGGTGCAGGCCATGCTCGGCCAGAGCACCGAGGAGCTGCGGGTGCGCCTCGCCTCCCACCTGCGCAAGCTGCGTAAGCGGCTCCTCCGCGATGCCGATGACCTGCAGAAGCGCCTGGCAGTGTACCAGGCCGGGGCGAAT

>Seq96 [organism=Homo sapiens] LUAD ApoE ε3ε3

GTCGGGAGGCGCATGTCGCTGTGGGCGCGGAATGGCAGGACGTGTGCGGCCGCCTGGTGCAGTACCGGGCGCGGAATGGAGGACGTGTGCGGCCGCCTGGTGCAGTACCGCGGCGAGGTGCAGGCCATGCTCGGCCAGAGCACCGAGGAGCTGCGGGTGCGCCTCGCCTCCCACCTGCGCAAGCTGCGTAAGCGGCTCCTCCGCGATGCCGATGACCTGCAGAAGCGCCTGGCAGTGTACCAGGCCGGGGCGAAT

>Seq97 [organism=Homo sapiens] LUAD ApoE ε3ε3

GTGCGGAGGCGCATGTCGCTGTGGGCGCGGAATGGCAGGACGTGTGCGGCCGCCTGGTGCAGTACCGGGCGCGGAATGGAGGACGTGTGCGGCCGCCTGGTGCAGTACCGCGGCGAGGTGCAGGCCATGCTCGGCCAGAGCACCGAGGAGCTGCGGGTGCGCCTCGCCTCCCACCTGCGCAAGCTGCGTAAGCGGCTCCTCCGCGATGCCGATGACCTGCAGAAGCGCCTGGCAGTGTACCAGGCCGGGGCGAAT

>Seq98 [organism=Homo sapiens] LUAD ApoE ε3ε3

GTGACGGGAGGCGCATGTCGCTGTGGGCGCGGAATGGCAGGACGTGTGCGGCCGCCTGGTGCAGTACCGGGCGCGGAATGGAGGACGTGTGCGGCCGCCTGGTGCAGTACCGCGGCGAGGTGCAGGCCATGCTCGGCCAGAGCACCGAGGAGCTGCGGGTGCGCCTCGCCTCCCACCTGCGCAAGCTGCGTAAGCGGCTCCTCCGCGATGCCGATGACCTGCAGAAGCGCCTGGCAGTGTACCAGGCCGGGGCGAAT

>Seq99 [organism=Homo sapiens] LUAD ApoE ε3ε3

GTAGCGGGGCGCATGTCGCTGTGGGCGCGGAATGGCAGGACGTGTGCGGCCGCCTGGTGCAGTACCGGGCGCGGAATGGAGGACGTGTGCGGCCGCCTGGTGCAGTACCGCGGCGAGGTGCAGGCCATGCTCGGCCAGAGCACCGAGGAGCTGCGGGTGCGCCTCGCCTCCCACCTGCGCAAGCTGCGTAAGCGGCTCCTCCGCGATGCCGATGACCTGCAGAAGCGCCTGGCAGTGTACCAGGCCGGGGCGAAT

>Seq100 [organism=Homo sapiens] LUAD ApoE ε3ε3

GTGGAGGCCGGCATGTCGCTGTGGGCGCGGAATGGCAGGACGTGTGCGGCCGCCTGGTGCAGTACCGGGCGCGGAATGGAGGACGTGTGCGGCCGCCTGGTGCAGTACCGCGGCGAGGTGCAGGCCATGCTCGGCCAGAGCACCGAGGAGCTGCGGGTGCGCCTCGCCTCCCACCTGCGCAAGCTGCGTAAGCGGCTCCTCCGCGATGCCGATGACCTGCAGAAGCGCCTGGCAGTGTACCAGGCCGGGGCGAAT

>Seq101 [organism=Homo sapiens] LUAD ApoE ε3ε3

GTCGAGGCGGGCATGTCGCTGTGGGCGCGGAATGGCAGGACGTGTGCGGCCGCCTGGTGCAGTACCGGGCGCGGAATGGAGGACGTGTGCGGCCGCCTGGTGCAGTACCGCGGCGAGGTGCAGGCCATGCTCGGCCAGAGCACCGAGGAGCTGCGGGTGCGCCTCGCCTCCCACCTGCGCAAGCTGCGTAAGCGGCTCCTCCGCGATGCCGATGACCTGCAGAAGCGCCTGGCAGTGTACCAGGCCGGGGCGAAT

>Seq102 [organism=Homo sapiens] LUAD ApoE ε3ε3

GTAGGGAGGCGCATGTCGCTGTGGGCGCGGAATGGCAGGACGTGTGCGGCCGCCTGGTGCAGTACCGGGCGCGGAATGGAGGACGTGTGCGGCCGCCTGGTGCAGTACCGCGGCGAGGTGCAGGCCATGCTCGGCCAGAGCACCGAGGAGCTGCGGGTGCGCCTCGCCTCCCACCTGCGCAAGCTGCGTAAGCGGCTCCTCCGCGATGCCGATGACCTGCAGAAGCGCCTGGCAGTGTACCAGGCCGGGGCGAAT

>Seq103 [organism=Homo sapiens] LUAD ApoE ε3ε3

GTGGGGAGGCGCATGTCGCTGTGGGCGCGGAATGGCAGGACGTGTGCGGCCGCCTGGTGCAGTACCGGGCGCGGAATGGAGGACGTGTGCGGCCGCCTGGTGCAGTACCGCGGCGAGGTGCAGGCCATGCTCGGCCAGAGCACCGAGGAGCTGCGGGTGCGCCTCGCCTCCCACCTGCGCAAGCTGCGTAAGCGGCTCCTCCGCGATGCCGATGACCTGCAGAAGCGCCTGGCAGTGTACCAGGCCGGGGCGAAT

>Seq104 [organism=Homo sapiens] LUAD ApoE ε3ε3

GTCGCGAGGCGCATGTCGCTGTGGGCGCGGAATGGCAGGACGTGTGCGGCCGCCTGGTGCAGTACCGGGCGCGGAATGGAGGACGTGTGCGGCCGCCTGGTGCAGTACCGCGGCGAGGTGCAGGCCATGCTCGGCCAGAGCACCGAGGAGCTGCGGGTGCGCCTCGCCTCCCACCTGCGCAAGCTGCGTAAGCGGCTCCTCCGCGATGCCGATGACCTGCAGAAGCGCCTGGCAGTGTACCAGGCCGGGGCGAAT

>Seq105 [organism=Homo sapiens] LUAD ApoE ε3ε3

GGTGGGAGGCGCATGTCGCTGTGGGCGCGGAATGGCAGGACGTGTGCGGCCGCCTGGTGCAGTACCGGGCGCGGAATGGAGGACGTGTGCGGCCGCCTGGTGCAGTACCGCGGCGAGGTGCAGGCCATGCTCGGCCAGAGCACCGAGGAGCTGCGGGTGCGCCTCGCCTCCCACCTGCGCAAGCTGCGTAAGCGGCTCCTCCGCGATGCCGATGACCTGCAGAAGCGCCTGGCAGTGTACCAGGCCGGGGCGAAT

>Seq106 [organism=Homo sapiens] LUAD ApoE ε3ε3

GTCGGCAGGCGCATGTCGCTGTGGGCGCGGAATGGCAGGACGTGTGCGGCCGCCTGGTGCAGTACCGGGCGCGGAATGGAGGACGTGTGCGGCCGCCTGGTGCAGTACCGCGGCGAGGTGCAGGCCATGCTCGGCCAGAGCACCGAGGAGCTGCGGGTGCGCCTCGCCTCCCACCTGCGCAAGCTGCGTAAGCGGCTCCTCCGCGATGCCGATGACCTGCAGAAGCGCCTGGCAGTGTACCAGGCCGGGGCGAAT

>Seq107 [organism=Homo sapiens] LUAD ApoE ε3ε3

GTCGGAAGGCGCATGTCGCTGTGGGCGCGGAATGGCAGGACGTGTGCGGCCGCCTGGTGCAGTACCGGGCGCGGAATGGAGGACGTGTGCGGCCGCCTGGTGCAGTACCGCGGCGAGGTGCAGGCCATGCTCGGCCAGAGCACCGAGGAGCTGCGGGTGCGCCTCGCCTCCCACCTGCGCAAGCTGCGTAAGCGGCTCCTCCGCGATGCCGATGACCTGCAGAAGCGCCTGGCAGTGTACCAGGCCGGGGCGAAT

>Seq108 [organism=Homo sapiens] LUAD ApoE ε3ε3

GTGGGGAGGCGCATGTCGCTGTGGGCGCGGAATGGCAGGACGTGTGCGGCCGCCTGGTGCAGTACCGGGCGCGGAATGGAGGACGTGTGCGGCCGCCTGGTGCAGTACCGCGGCGAGGTGCAGGCCATGCTCGGCCAGAGCACCGAGGAGCTGCGGGTGCGCCTCGCCTCCCACCTGCGCAAGCTGCGTAAGCGGCTCCTCCGCGATGCCGATGACCTGCAGAAGCGCCTGGCAGTGTACCAGGCCGGGGCGAAT

>Seq109 [organism=Homo sapiens] LUAD ApoE ε3ε3

GTCCGGAGGCGCATGTCGCTGTGGGCGCGGAATGGCAGGACGTGTGCGGCCGCCTGGTGCAGTACCGGGCGCGGAATGGAGGACGTGTGCGGCCGCCTGGTGCAGTACCGCGGCGAGGTGCAGGCCATGCTCGGCCAGAGCACCGAGGAGCTGCGGGTGCGCCTCGCCTCCCACCTGCGCAAGCTGCGTAAGCGGCTCCTCCGCGATGCCGATGACCTGCAGAAGCGCCTGGCAGTGTACCAGGCCGGGGCGAAT

>Seq110 [organism=Homo sapiens] LUAD ApoE ε3ε3

GTCTGGAGGCGCATGTCGCTGTGGGCGCGGAATGGCAGGACGTGTGCGGCCGCCTGGTGCAGTACCGGGCGCGGAATGGAGGACGTGTGCGGCCGCCTGGTGCAGTACCGCGGCGAGGTGCAGGCCATGCTCGGCCAGAGCACCGAGGAGCTGCGGGTGCGCCTCGCCTCCCACCTGCGCAAGCTGCGTAAGCGGCTCCTCCGCGATGCCGATGACCTGCAGAAGCGCCTGGCAGTGTACCAGGCCGGGGCGAAT

>Seq111 [organism=Homo sapiens] LUAD ApoE ε3ε3

GGTGGGAGGCGCATGTCGCTGTGGGCGCGGAATGGCAGGACGTGTGCGGCCGCCTGGTGCAGTACCGGGCGCGGAATGGAGGACGTGTGCGGCCGCCTGGTGCAGTACCGCGGCGAGGTGCAGGCCATGCTCGGCCAGAGCACCGAGGAGCTGCGGGTGCGCCTCGCCTCCCACCTGCGCAAGCTGCGTAAGCGGCTCCTCCGCGATGCCGATGACCTGCAGAAGCGCCTGGCAGTGTACCAGGCCGGGGCGAAT

>Seq112 [organism=Homo sapiens] LUAD ApoE ε3ε3

CGTGGGAGGCGCATGTCGCTGTGGGCGCGGAATGGCAGGACGTGTGCGGCCGCCTGGTGCAGTACCGGGCGCGGAATGGAGGACGTGTGCGGCCGCCTGGTGCAGTACCGCGGCGAGGTGCAGGCCATGCTCGGCCAGAGCACCGAGGAGCTGCGGGTGCGCCTCGCCTCCCACCTGCGCAAGCTGCGTAAGCGGCTCCTCCGCGATGCCGATGACCTGCAGAAGCGCCTGGCAGTGTACCAGGCCGGGGCGAAT

>Seq113 [organism=Homo sapiens] LUAD ApoE ε3ε3

TGTGGGAGGCGCATGTCGCTGTGGGCGCGGAATGGCAGGACGTGTGCGGCCGCCTGGTGCAGTACCGGGCGCGGAATGGAGGACGTGTGCGGCCGCCTGGTGCAGTACCGCGGCGAGGTGCAGGCCATGCTCGGCCAGAGCACCGAGGAGCTGCGGGTGCGCCTCGCCTCCCACCTGCGCAAGCTGCGTAAGCGGCTCCTCCGCGATGCCGATGACCTGCAGAAGCGCCTGGCAGTGTACCAGGCCGGGGCGAAT

>Seq114 [organism=Homo sapiens] LUAD ApoE ε3ε3

AGTGGGAGGCGCATGTCGCTGTGGGCGCGGAATGGCAGGACGTGTGCGGCCGCCTGGTGCAGTACCGGGCGCGGAATGGAGGACGTGTGCGGCCGCCTGGTGCAGTACCGCGGCGAGGTGCAGGCCATGCTCGGCCAGAGCACCGAGGAGCTGCGGGTGCGCCTCGCCTCCCACCTGCGCAAGCTGCGTAAGCGGCTCCTCCGCGATGCCGATGACCTGCAGAAGCGCCTGGCAGTGTACCAGGCCGGGGCGAAT

>Seq115 [organism=Homo sapiens] LUAD ApoE ε3ε3

GGTGGGAGGCGCATGTCGCTGTGGGCGCGGAATGGCAGGACGTGTGCGGCCGCCTGGTGCAGTACCGGGCGCGGAATGGAGGACGTGTGCGGCCGCCTGGTGCAGTACCGCGGCGAGGTGCAGGCCATGCTCGGCCAGAGCACCGAGGAGCTGCGGGTGCGCCTCGCCTCCCACCTGCGCAAGCTGCGTAAGCGGCTCCTCCGCGATGCCGATGACCTGCAGAAGCGCCTGGCAGTGTACCAGGCCGGGGCGAAT

>Seq116 [organism=Homo sapiens] LUAD ApoE ε3ε3

CGTGGGAGGCGCATGTCGCTGTGGGCGCGGAATGGCAGGACGTGTGCGGCCGCCTGGTGCAGTACCGGGCGCGGAATGGAGGACGTGTGCGGCCGCCTGGTGCAGTACCGCGGCGAGGTGCAGGCCATGCTCGGCCAGAGCACCGAGGAGCTGCGGGTGCGCCTCGCCTCCCACCTGCGCAAGCTGCGTAAGCGGCTCCTCCGCGATGCCGATGACCTGCAGAAGCGCCTGGCAGTGTACCAGGCCGGGGCGAAT

>Seq117 [organism=Homo sapiens] LUAD ApoE ε3ε3

TGTGGGAGGCGCATGTCGCTGTGGGCGCGGAATGGCAGGACGTGTGCGGCCGCCTGGTGCAGTACCGGGCGCGGAATGGAGGACGTGTGCGGCCGCCTGGTGCAGTACCGCGGCGAGGTGCAGGCCATGCTCGGCCAGAGCACCGAGGAGCTGCGGGTGCGCCTCGCCTCCCACCTGCGCAAGCTGCGTAAGCGGCTCCTCCGCGATGCCGATGACCTGCAGAAGCGCCTGGCAGTGTACCAGGCCGGGGCGAAT

>Seq118 [organism=Homo sapiens] LUAD ApoE ε3ε3

AGTGGGAGGCGCATGTCGCTGTGGGCGCGGAATGGCAGGACGTGTGCGGCCGCCTGGTGCAGTACCGGGCGCGGAATGGAGGACGTGTGCGGCCGCCTGGTGCAGTACCGCGGCGAGGTGCAGGCCATGCTCGGCCAGAGCACCGAGGAGCTGCGGGTGCGCCTCGCCTCCCACCTGCGCAAGCTGCGTAAGCGGCTCCTCCGCGATGCCGATGACCTGCAGAAGCGCCTGGCAGTGTACCAGGCCGGGGCGAAT

>Seq119 [organism=Homo sapiens] LUAD ApoE ε3ε3

GGTGGGAGGCGCATGTCGCTGTGGGCGCGGAATGGCAGGACGTGTGCGGCCGCCTGGTGCAGTACCGGGCGCGGAATGGAGGACGTGTGCGGCCGCCTGGTGCAGTACCGCGGCGAGGTGCAGGCCATGCTCGGCCAGAGCACCGAGGAGCTGCGGGTGCGCCTCGCCTCCCACCTGCGCAAGCTGCGTAAGCGGCTCCTCCGCGATGCCGATGACCTGCAGAAGCGCCTGGCAGTGTACCAGGCCGGGGCGAAT

>Seq120 [organism=Homo sapiens] LUAD ApoE ε3ε3

GTCGGGGGCGCATGTCGCTGTGGGCGCGGAATGGCAGGACGTGTGCGGCCGCCTGGTGCAGTACCGCGGCGAGGTGCAGGCCATGCTCGGCCAGAGCACCGAGGAGCTGCGGGTGCGCCTCGCCTCCCACCTGCGCAAGCTGCGTAAGCGGCTCCTCCGCGATGCCGATGACCTGCAGAAGCGCCTGGCAGTGTACCAGGCCGGGGCGAATA

>Seq121 [organism=Homo sapiens] LUAD ApoE ε3ε3

GTGGGAGGCGCATGTCGCTGTGGGCGCGGAATGGCAGGACGTGTGCGGCCGCCTGGTGCAGTACCGCGGCGAGGTGCAGGCCATGCTCGGCCAGAGCACCGAGGAGCTGCGGGTGCGCCTCGCCTCCCACCTGCGCAAGCTGCGTAAGCGGCTCCTCCGCGATGCCGATGACCTGCAGAAGCGCCTGGCAGTGTACCAGGCCGGGGCGAATA

>Seq122 [organism=Homo sapiens] LUAD ApoE ε3ε3

GAGTCGTCGCGTTCCTGGTATACGGTCCCGGAATGGCAGGACGTGTGCGGCCGCCTGGTGCAGTACCGCGGCGAGGTGCAGGCCATGCTCGGCCAGAGCACCGAGGAGCTGCGGGTGCGCCTCGCCTCCCACCTGCGCAAGCTGCGTAAGCGGCTCCTCCGCGATGCCGATGACCTGCAGAAGCGCCTGGCAGTGTACCAGGCCGGGGCGAATA

>Seq123 [organism=Homo sapiens] LUAD ApoE ε3ε3

CGTCGCGCGTTCCTGGTATACGGTCCCGGAATGGCAGGACGTGTGCGGCCGCCTGGTGCAGTACCGCGGCGAGGTGCAGGCCATGCTCGGCCAGAGCACCGAGGAGCTGCGGGTGCGCCTCGCCTCCCACCTGCGCAAGCTGCGTAAGCGGCTCCTCCGCGATGCCGATGACCTGCAGAAGCGCCTGGCAGTGTACCAGGCCGGGGCGAATA

>Seq124 [organism=Homo sapiens] LUAD ApoE ε3ε3

GGGAGGCGCATGTCGCTGTGGGCGCGGAATGGCAGGACGTGTGCGGCCGCCTGGTGCAGTACCGCGGCGAGGTGCAGGCCATGCTCGGCCAGAGCACCGAGGAGCTGCGGGTGCGCCTCGCCTCCCACCTGCGCAAGCTGCGTAAGCGGCTCCTCCGCGATGCCGATGACCTGCAGAAGCGCCTGGCAGTGTACCAGGCCGGGGCGAATA

>Seq125 [organism=Homo sapiens] LUAD ApoE ε3ε3

GTTCGTCGCGTTCCTGGTATACGGTCCCGGAATGGCAGGACGTGTGCGGCCGCCTGGTGCAGTACCGCGGCGAGGTGCAGGCCATGCTCGGCCAGAGCACCGAGGAGCTGCGGGTGCGCCTCGCCTCCCACCTGCGCAAGCTGCGTAAGCGGCTCCTCCGCGATGCCGATGACCTGCAGAAGCGCCTGGCAGTGTACCAGGCCGGGGCGAATA

>Seq126 [organism=Homo sapiens] LUAD ApoE ε3ε3

GTGGGAGGCGCATGTCGCTGTGGGCGCGGAATGGCAGGACGTGTGCGGCCGCCTGGTGCAGTACCGCGGCGAGGTGCAGGCCATGCTCGGCCAGAGCACCGAGGAGCTGCGGGTGCGCCTCGCCTCCCACCTGCGCAAGCTGCGTAAGCGGCTCCTCCGCGATGCCGATGACCTGCAGAAGCGCCTGGCAGTGTACCAGGCCGGGGCGAATA

>Seq127 [organism=Homo sapiens] LUAD ApoE ε3ε3

CAGTGACGCTCCAGTCGTCTTACGTATCGGAATGGCAGGACGTGTGCGGCCGCCTGGTGCAGTACCGCGGCGAGGTGCAGGCCATGCTCGGCCAGAGCACCGAGGAGCTGCGGGTGCGCCTCGCCTCCCACCTGCGCAAGCTGCGTAAGCGGCTCCTCCGCGATGCCGATGACCTGCAGAAGCGCCTGGCAGTGTACCAGGCCGGGGCGAATA

>Seq128 [organism=Homo sapiens] LUAD ApoE ε3ε3

CGTCGCGCGTTCCTGGTATACGGTCCCGGAATGGCAGGACGTGTGCGGCCGCCTGGTGCAGTACCGCGGCGAGGTGCAGGCCATGCTCGGCCAGAGCACCGAGGAGCTGCGGGTGCGCCTCGCCTCCCACCTGCGCAAGCTGCGTAAGCGGCTCCTCCGCGATGCCGATGACCTGCAGAAGCGCCTGGCAGTGTACCAGGCCGGGGCGAATA

>Seq129 [organism=Homo sapiens] LUAD ApoE ε3ε3

GAGTCGTCGCGTTCCTGGTATACGGTCCCGGAATGGCAGGACGTGTGCGGCCGCCTGGTGCAGTACCGCGGCGAGGTGCAGGCCATGCTCGGCCAGAGCACCGAGGAGCTGCGGGTGCGCCTCGCCTCCCACCTGCGCAAGCTGCGTAAGCGGCT

>Seq130 [organism=Homo sapiens] LUAD ApoE ε3ε3

GGTGGGAGGCGCATGTCGCTGTGGGCGCGGAATGGCAGGACGTGTGCGGCCGCCTGGTGCAGTACCGCGGCGAGGTGCAGGCCATGCTCGGCCAGAGCACCGAGGAGCTGCGGGTGCGCCTCGCCTCCCACCTGCGCAAGCTGCGTAAGCGGCTCCTCCGCGATGCCGATGACCTGCAGAAGCGCCTGGCAGTGTACCAGGCCGGGGCGAATA

>Seq131 [organism=Homo sapiens] LUAD ApoE ε3ε3

CAGTGACGCTCCAGTCGTCTTACGTATCGGAATGGCAGGACGTGTGCGGCCGCCTGGTGCAGTACCGCGGCGAGGTGCAGGCCATGCTCGGCCAGAGCACCGAGGAGCTGCGGGTGCGCCTCGCCTCCCACCTGCGCAAGCTGCGTAAGCGGCTCCTCCGCGATGCCGATGACCTGCAGAAGCGCCTGGCAGTGTACCAGGCCGGGGCGAATA

>Seq132 [organism=Homo sapiens] LUAD ApoE ε3ε3

GTTCGTCGCGTTCCTGGTATACGGTCCCGGAATGGCAGGACGTGTGCGGCCGCCTGGTGCAGTACCGCGGCGAGGTGCAGGCCATGCTCGGCCAGAGCACCGAGGAGCTGCGGGTGCGCCTCGCCTCCCACCTGCGCAAGCTGCGTAAGCGGCTCCTCCGCGATGCCGATGACCTGCAGAAGCGCCTGGCAGTGTACCAGGCCGGGGCGAATA

>Seq133 [organism=Homo sapiens] LUAD ApoE ε3ε3

GTGGGAGGCGCATGTCGCTGTGGGCGCGGAATGGCAGGACGTGTGCGGCCGCCTGGTGCAGTACCGCGGCGAGGTGCAGGCCATGCTCGGCCAGAGCACCGAGGAGCTGCGGGTGCGCCTCGCCTCCCACCTGCGCAAGCTGCGTAAGCGGCTCCTCCGCGATGCCGATGACCTGCAGAAGCGCCTGGCAGTGTACCAGGCCGGGGCGAATA

>Seq134 [organism=Homo sapiens] LUAD ApoE ε3ε3

GAGTCGTCGCGTTCCTGGTATACGGTCCCGGAATGGCAGGACGTGTGCGGCCGCCTGGTGCAGTACCGCGGCGAGGTGCAGGCCATGCTCGGCCAGAGCACCGAGGAGCTGCGGGTGCGCCTCGCCTCCCACCTGCGCAAGCTGCGTAAGCGGCTCCTCCGCGATGCCGATGACCTGCAGAAGCGCCTGGCAGTGTACCAGGCCGGGGCGAATA

>Seq135 [organism=Homo sapiens] LUAD ApoE ε3ε3

CAGTGACGCTCCAGTCGTCTTACGTATCGGAATGGCAGGACGTGTGCGGCCGCCTGGTGCAGTACCGCGGCGAGGTGCAGGCCATGCTCGGCCAGAGCACCGAGGAGCTGCGGGTGCGCCTCGCCTCCCACCTGCGCAAGCTGCGTAAGCGGCTCCTCCGCGATGCCGATGACCTGCAGAAGCGCCTGGCAGTGTACCAGGCCGGGGCGAATA

>Seq136 [organism=Homo sapiens] LUAD ApoE ε3ε3

CAGTGACGCTCCAGTCGTCTTACGTATCGGAATGGCAGGACGTGTGCGGCCGCCTGGTGCAGTACCGCGGCGAGGTGCAGGCCATGCTCGGCCAGAGCACCGAGGAGCTGCGGGTGCGCCTCGCCTCCCACCTGCGCAAGCTGCGTAAGCGGCTCCTCCGCGATGCCGATGACCTGCAGAAGCGCCTGGCAGTGTACCAGGCCGGGGCGAATA

>Seq137 [organism=Homo sapiens] LUAD ApoE ε3ε3

GTGGGAGGCGCATGTCGCTGTGGGCGCGGAATGGCAGGACGTGTGCGGCCGCCTGGTGCAGTACCGCGGCGAGGTGCAGGCCATGCTCGGCCAGAGCACCGAGGAGCTGCGGGTGCGCCTCGCCTCCCACCTGCGCAAGCTGCGTAAGCGGCTCCTCCGCGATGCCGATGACCTGCAGAAGCGCCTGGCAGTGTACCAGGCCGGGGCGAATA

>Seq138 [organism=Homo sapiens] LUAD ApoE ε3ε3

GAGTCGTCGCGTTCCTGGTATACGGTCCCGGAATGGCAGGACGTGTGCGGCCGCCTGGTGCAGTACCGCGGCGAGGTGCAGGCCATGCTCGGCCAGAGCACCGAGGAGCTGCGGGTGCGCCTCGCCTCCCACCTGCGCAAGCTGCGTAAGCGGCTCCTCCGCGATGCCGATGACCTGCAGAAGCGCCTGGCAGTGTACCAGGCCGGGGCGAATA

>Seq139 [organism=Homo sapiens] LUAD ApoE ε3ε3

CGTCGCGCGTTCCTGGTATACGGTCCCGGAATGGCAGGACGTGTGCGGCCGCCTGGTGCAGTACCGCGGCGAGGTGCAGGCCATGCTCGGCCAGAGCACCGAGGAGCTGCGGGTGCGCCTCGCCTCCCACCTGCGCAAGCTGCGTAAGCGGCTCCTCCGCGATGCCGATGACCTGCAGAAGCGCCTGGCAGTGTACCAGGCCGGGGCGAATA

>Seq140 [organism=Homo sapiens] LUAD ApoE ε3ε3

GTGGGAGGTTCCTGGTATACGGTCCCGGAATGGCAGGACGTGTGCGGCCGCCTGGTGCAGTACCGCGGCGAGGTGCAGGCCATGCTCGGCCAGAGCACCGAGGAGCTGCGGGTGCGCCTCGCCTCCCACCTGCGCAAGCTGCGTAAGCGGCTCCTCCGCGATGCCGATGACCTGCAGAAGCGCCTGGCAGTGTACCAGGCCGGGGCGAATA

>Seq141 [organism=Homo sapiens] LUAD ApoE ε3ε3

GTGGGAGGCGCATGTCGCTGTGGGCGCGGAATGGCAGGACGTGTGCGGCCGCCTGGTGCAGTACCGCGGCGAGGTGCAGGCCATGCTCGGCCAGAGCACCGAGGAGCTGCGGGTGCGCCTCGCCTCCCACCTGCGCAAGCTGCGTAAGCGGCTCCTCCGCGATGCCGATGACCTGCAGAAGCGCCTGGCAGTGTACCAGGCCGGGGCGAATA

>Seq142 [organism=Homo sapiens] LUAD ApoE ε3ε3

GAGTCGTCGCGTTCCTGGTATACGGTCCCGGAATGGCAGGACGTGTGCGGCCGCCTGGTGCAGTACCGCGGCGAGGTGCAGGCCATGCTCGGCCAGAGCACCGAGGAGCTGCGGGTGCGCCTCGCCTCCCACCTGCGCAAGCTGCGTAAGCGGCTCCTCCGCGATGCCGATGACCTGCAGAAGCGCCTGGCAGTGTACCAGGCCGGGGCGAATA

>Seq143 [organism=Homo sapiens] LUAD ApoE ε3ε3

CGTCGCGCGTTCCTGGTATACGGTCCCGGAATGGCAGGACGTGTGCGGCCGCCTGGTGCAGTACCGCGGCGAGGTGCAGGCCATGCTCGGCCAGAGCACCGAGGAGCTGCGGGTGCGCCTCGCCTCCCACCTGCGCAAGCTGCGTAAGCGGCTCCTCCGCGATGCCGATGACCTGCAGAAGCGCCTGGCAGTGTACCAGGCCGGGGCGAATA

>Seq144 [organism=Homo sapiens] LUAD ApoE ε3ε3

GGGAGGCGCATGTCGCTGTGGGCGCGGAATGGCAGGACGTGTGCGGCCGCCTGGTGCAGTACCGCGGCGAGGTGCAGGCCATGCTCGGCCAGAGCACCGAGGAGCTGCGGGTGCGCCTCGCCTCCCACCTGCGCAAGCTGCGTAAGCGGCTCCTCCGCGATGCCGATGACCTGCAGAAGCGCCTGGCAGTGTACCAGGCCGGGGCGAATA

>Seq145 [organism=Homo sapiens] LUAD ApoE ε3ε3

GTTCGTCGCGTTCCTGGTATACGGTCCCGGAATGGCAGGACGTGTGCGGCCGCCTGGTGCAGTACCGCGGCGAGGTGCAGGCCATGCTCGGCCAGAGCACCGAGGAGCTGCGGGTGCGCCTCGCCTCCCACCTGCGCAAGCTGCGTAAGCGGCTCCTCCGCGATGCCGATGACCTGCAGAAGCGCCTGGCAGTGTACCAGGCCGGGGCGAATA

>Seq146 [organism=Homo sapiens] LUAD ApoE ε3ε3

GTGGGAGGCGCATGTCGCTGTGGGCGCGGAATGGCAGGACGTGTGCGGCCGCCTGGTGCAGTACCGCGGCGAGGTGCAGGCCATGCTCGGCCAGAGCACCGAGGAGCTGCGGGTGCGCCTCGCCTCCCACCTGCGCAAGCTGCGTAAGCGGCTCCTCCGCGATGCCGATGACCTGCAGAAGCGCCTGGCAGTGTACCAGGCCGGGGCGAATA

>Seq147 [organism=Homo sapiens] LUAD ApoE ε3ε3

CAGTGACGCTCCAGTCGTCTTACGTATCGGAATGGCAGGACGTGTGCGGCCGCCTGGTGCAGTACCGCGGCGAGGTGCAGGCCATGCTCGGCCAGAGCACCGAGGAGCTGCGGGTGCGCCTCGCCTCCCACCTGCGCAAGCTGCGTAAGCGGCTCCTCCGCGATGCCGATGACCTGCAGAAGCGCCTGGCAGTGTACCAGGCCGGGGCGAATA

>Seq148 [organism=Homo sapiens] LUAD ApoE ε3ε3

CGTCGCGCGTTCCTGGTATACGGTCCCGGAATGGCAGGACGTGTGCGGCCGCCTGGTGCAGTACCGCGGCGAGGTGCAGGCCATGCTCGGCCAGAGCACCGAGGAGCTGCGGGTGCGCCTCGCCTCCCACCTGCGCAAGCTGCGTAAGCGGCTCCTCCGCGATGCCGATGACCTGCAGAAGCGCCTGGCAGTGTACCAGGCCGGGGCGAATA

>Seq149 [organism=Homo sapiens] LUAD ApoE ε3ε3

GAGTCGTCGCGTTCCTGGTATACGGTCCCGGAATGGCAGGACGTGTGCGGCCGCCTGGTGCAGTACCGCGGCGAGGTGCAGGCCATGCTCGGCCAGAGCACCGAGGAGCTGCGGGTGCGCCTCGCCTCCCACCTGCGCAAGCTGCGTAAGCGGCTCCTCCGCGATGCCGATGACCTGCAGAAGCGCCTGGCAGTGTACCAGGCCGGGGCGAATA

>Seq150 [organism=Homo sapiens] LUAD ApoE ε3ε3

CACGTAGCGGGGCGCGGAATGGAGGACGTGTGCGGCCGCCTGGTGCAGTACCGCGGCGAGGTGCAGGCCATGCTCGGCCAGAGCACCGAGGAGCTGCGGGTGCGCCTCGCCTCCCACCTGCGCAAGCTGCGTAAGCGGCTCCTCCGCGATGCCGATGACCTGCAGAAGCGCCTGGCAGTGTACCAGGCCGGGGCGAAT

>Seq151 [organism=Homo sapiens] LUAD ApoE ε3ε3

TACGTAGCGGGGCGCGGAATGGAGGACGTGTGCGGCCGCCTGGTGCAGTACCGCGGCGAGGTGCAGGCCATGCTCGGCCAGAGCACCGAGGAGCTGCGGGTGCGCCTCGCCTCCCACCTGCGCAAGCTGCGTAAGCGGCTCCTCCGCGATGCCGATGACCTGCAGAAGCGCCTGGCAGTGTACCAGGCCGGGGCGAAT

>Seq152 [organism=Homo sapiens] LUAD ApoE ε3ε3

GACGTAGCGGGGCGCGGAATGGAGGACGTGTGCGGCCGCCTGGTGCAGTACCGCGGCGAGGTGCAGGCCATGCTCGGCCAGAGCACCGAGGAGCTGCGGGTGCGCCTCGCCTCCCACCTGCGCAAGCTGCGTAAGCGGCTCCTCCGCGATGCCGATGACCTGCAGAAGCGCCTGGCAGTGTACCAGGCCGGGGCGAAT

>Seq153 [organism=Homo sapiens] LUAD ApoE ε3ε3

TCGTAGCGGGGCGCGGAATGGAGGACGTGTGCGGCCGCCTGGTGCAGTACCGCGGCGAGGTGCAGGCCATGCTCGGCCAGAGCACCGAGGAGCTGCGGGTGCGCCTCGCCTCCCACCTGCGCAAGCTGCGTAAGCGGCTCCTCCGCGATGCCGATGACCTGCAGAAGCGCCTGGCAGTGTACCAGGCCGGGGCGAAT

>Seq154 [organism=Homo sapiens] LUAD ApoE ε3ε3

TACGTGCGGGGCGCGGAATGGAGGACGTGTGCGGCCGCCTGGTGCAGTACCGCGGCGAGGTGCAGGCCATGCTCGGCCAGAGCACCGAGGAGCTGCGGGTGCGCCTCGCCTCCCACCTGCGCAAGCTGCGTAAGCGGCTCCTCCGCGATGCCGATGACCTGCAGAAGCGCCTGGCAGTGTACCAGGCCGGGGCGAAT

>Seq155 [organism=Homo sapiens] LUAD ApoE ε3ε3

GACGTAGCGGGGCGCGGAATGGAGGACGTGTGCGGCCGCCTGGTGCAGTACCGCGGCGAGGTGCAGGCCATGCTCGGCCAGAGCACCGAGGAGCTGCGGGTGCGCCTCGCCTCCCACCTGCGCAAGCTGCGTAAGCGGCTCCTCCGCGATGCCGATGACCTGCAGAAGCGCCTGGCAGTGTACCAGGCCGGGGCGAAT

>Seq156 [organism=Homo sapiens] LUAD ApoE ε3ε3

TACGTAGCGGGGCGCGGAATGGAGGACGTGTGCGGCCGCCTGGTGCAGTACCGCGGCGAGGTGCAGGCCATGCTCGGCCAGAGCACCGAGGAGCTGCGGGTGCGCCTCGCCTCCCACCTGCGCAAGCTGCGTAAGCGGCTCCTCCGCGATGCCGATGACCTGCAGAAGCGCCTGGCAGTGTACCAGGCCGGGGCGAAT

>Seq157 [organism=Homo sapiens] LUAD ApoE ε3ε3

GACGTAGCGGGGCGCGGAATGGAGGACGTGTGCGGCCGCCTGGTGCAGTACCGCGGCGAGGTGCAGGCCATGCTCGGCCAGAGCACCGAGGAGCTGCGGGTGCGCCTCGCCTCCCACCTGCGCAAGCTGCGTAAGCGGCTCCTCCGCGATGCCGATGACCTGCAGAAGCGCCTGGCAGTGTACCAGGCCGGGGCGAAT

>Seq158 [organism=Homo sapiens] LUAD ApoE ε3ε3

GACGTAGCGGGGCGCGGAATGGAGGACGTGTGCGGCCGCCTGGTGCAGTACCGCGGCGAGGTGCAGGCCATGCTCGGCCAGAGCACCGAGGAGCTGCGGGTGCGCCTCGCCTCCCACCTGCGCAAGCTGCGTAAGCGGCTCCTCCGCGATGCCGATGACCTGCAGAAGCGCCTGGCAGTGTACCAGGCCGGGGCGAAT

>Seq159 [organism=Homo sapiens] LUAD ApoE ε3ε3

GACGTAGCGGGGCGCGGAATGGAGGACGTGTGCGGCCGCCTGGTGCAGTACCGCGGCGAGGTGCAGGCCATGCTCGGCCAGAGCACCGAGGAGCTGCGGGTGCGCCTCGCCTCCCACCTGCGCAAGCTGCGTAAGCGGCTCCTCCGCGATGCCGATGACCTGCAGAAGCGCCTGGCAGTGTACCAGGCCGGGGCGAAT

>Seq160 [organism=Homo sapiens] LUAD ApoE ε3ε3

GAGGCCCTTCCTGGTATACGGTCCCGGAATGGCAGGACGTGTGCGGCCGCCTGGTGCAGTACCGCGGCGAGGTGCAGGCCATGCTCGGCCAGAGCACCGAGGAGCTGCGGGTGCGCCTCGCCTCCCACCTGCGCAAGCTGCGTAAGCGGCTCCTCCGCGATGCCGATGACCTGCAGAAGCGCCTGGCAGTGTACCAGGCCGGGGCGAATA

>Seq161 [organism=Homo sapiens] LUAD ApoE ε3ε3

GAGCAGCGGCCGCCTGGTGCAGTACCGCGGCGAGGTGCAGGCCATGCTCGGCCAGAGCACCGAGGAGCTGCGGGTGCGCCTCGCCTCCCACCTGCGCAAGCTGCGTAAGCGGCTCCTCCGCGATGCCGATGACCTGCAGAAGCGCCTGGCAGTGTACCAGGCCGGGGCGAATA

>Seq162 [organism=Homo sapiens] LUAD ApoE ε3ε3

TAGCAGCGGCCGCCTGGTGCAGTACCGCGGCGAGGTGCAGGCCATGCTCGGCCAGAGCACCGAGGAGCTGCGGGTGCGCCTCGCCTCCCACCTGCGCAAGCTGCGTAAGCGGCTCCTCCGCGATGCCGATGACCTGCAGAAGCGCCTGGCAGTGTACCAGGCCGGGGCGAATA

>Seq163 [organism=Homo sapiens] LUAD ApoE ε3ε3

CAGCAGCTTCCTGGTATACGGTCCCGGAATGGCAGGACGTGTGCGGCCGCCTGGTGCAGTACCGCGGCGAGGTGCAGGCCATGCTCGGCCAGAGCACCGAGGAGCTGCGGGTGCGCCTCGCCTCCCACCTGCGCAAGCTGCGTAAGCGGCTCCTCCGCGATGCCGATGACCTGCAGAAGCGCCTGGCAGTGTACCAGGCCGGGGCGAATA

>Seq164 [organism=Homo sapiens] LUAD ApoE ε3ε3

AAGCAGCGGCCGCCTGGTGCAGTACCGCGGCGAGGTGCAGGCCATGCTCGGCCAGAGCACCGAGGAGCTGCGGGTGCGCCTCGCCTCCCACCTGCGCAAGCTGCGTAAGCGGCTCCTCCGCGATGCCGATGACCTGCAGAAGCGCCTGGCAGTGTACCAGGCCGGGGCGAATA

>Seq165 [organism=Homo sapiens] LUAD ApoE ε3ε3

GCGCAGCGGCCGCCTGGTGCAGTACCGCGGCGAGGTGCAGGCCATGCTCGGCCAGAGCACCGAGGAGCTGCGGGTGCGCCTCGCCTCCCACCTGCGCAAGCTGCGTAAGCGGCTCCTCCGCGATGCCGATGACCTGCAGAAGCGCCTGGCAGTGTACCAGGCCGGGGCGAATA

>Seq166 [organism=Homo sapiens] LUAD ApoE ε3ε3

TCGCAGCGGCCGCCTGGTGCAGTACCGCGGCGAGGTGCAGGCCATGCTCGGCCAGAGCACCGAGGAGCTGCGGGTGCGCCTCGCCTCCCACCTGCGCAAGCTGCGTAAGCGGCTCCTCCGCGATGCCGATGACCTGCAGAAGCGCCTGGCAGTGTACCAGGCCGGGGCGAATA

>Seq167 [organism=Homo sapiens] LUAD ApoE ε3ε3

ACGCAGCGGCCGCCTGGTGCAGTACCGCGGCGAGGTGCAGGCCATGCTCGGCCAGAGCACCGAGGAGCTGCGGGTGCGCCTCGCCTCCCACCTGCGCAAGCTGCGTAAGCGGCTCCTCCGCGATGCCGATGACCTGCAGAAGCGCCTGGCAGTGTACCAGGCCGGGGCGAATA

>Seq168 [organism=Homo sapiens] LUAD ApoE ε3ε3

GAGCAGCGGCCGCCTGGTGCAGTACCGCGGCGAGGTGCAGGCCATGCTCGGCCAGAGCACCGAGGAGCTGCGGGTGCGCCTCGCCTCCCACCTGCGCAAGCTGCGTAAGCGGCTCCTCCGCGATGCCGATGACCTGCAGAAGCGCCTGGCAGTGTACCAGGCCGGGGCGAATA

>Seq169 [organism=Homo sapiens] LUAD ApoE ε3ε3

TGGCAGCTTCCTGGTATACGGTCCCGGAATGGCAGGACGTGTGCGGCCGCCTGGTGCAGTACCGCGGCGAGGTGCAGGCCATGCTCGGCCAGAGCACCGAGGAGCTGCGGGTGCGCCTCGCCTCCCACCTGCGCAAGCTGCGTAAGCGGCTCCTCCGCGATGCCGATGACCTGCAGAAGCGCCTGGCAGTGTACCAGGCCGGGGCGAATA

>Seq170 [organism=Homo sapiens] LUAD ApoE ε3ε3

CGGCAGCGGCCGCCTGGTGCAGTACCGCGGCGAGGTGCAGGCCATGCTCGGCCAGAGCACCGAGGAGCTGCGGGTGCGCCTCGCCTCCCACCTGCGCAAGCTGCGTAAGCGGCTCCTCCGCGATGCCGATGACCTGCAGAAGCGCCTGGCAGTGTACCAGGCCGGGGCGAATA

>Seq171 [organism=Homo sapiens] LUAD ApoE ε3ε3

AGGCAGCGGCCGCCTGGTGCAGTACCGCGGCGAGGTGCAGGCCATGCTCGGCCAGAGCACCGAGGAGCTGCGGGTGCGCCTCGCCTCCCACCTGCGCAAGCTGCGTAAGCGGCTCCTCCGCGATGCCGATGACCTGCAGAAGCGCCTGGCAGTGTACCAGGCCGGGGCGAATA

>Seq172 [organism=Homo sapiens] LUAD ApoE ε3ε3

TCGCAGCGGCCGCCTGGTGCAGTACCGCGGCGAGGTGCAGGCCATGCTCGGCCAGAGCACCGAGGAGCTGCGGGTGCGCCTCGCCTCCCACCTGCGCAAGCTGCGTAAGCGGCTCCTCCGCGATGCCGATGACCTGCAGAAGCGCCTGGCAGTGTACCAGGCCGGGGCGAATA

>Seq173 [organism=Homo sapiens] LUAD ApoE ε3ε3

GAGCAGCGGCCGCCTGGTGCAGTACCGCGGCGAGGTGCAGGCCATGCTCGGCCAGAGCACCGAGGAGCTGCGGGTGCGCCTCGCCTCCCACCTGCGCAAGCTGCGTAAGCGGCTCCTCCGCGATGCCGATGACCTGCAGAAGCGCCTGGCAGTGTACCAGGCCGGGGCGAATA

>Seq174 [organism=Homo sapiens] LUAD ApoE ε3ε3

CGGCAGCGGCCGCCTGGTGCAGTACCGCGGCGAGGTGCAGGCCATGCTCGGCCAGAGCACCGAGGAGCTGCGGGTGCGCCTCGCCTCCCACCTGCGCAAGCTGCGTAAGCGGCTCCTCCGCGATGCCGATGACCTGCAGAAGCGCCTGGCAGTGTACCAGGCCGGGGCGAATA

>Seq175 [organism=Homo sapiens] LUAD ApoE ε3ε3

GACGAGCGGCCGCCTGGTGCAGTACCGCGGCGAGGTGCAGGCCATGCTCGGCCAGAGCACCGAGGAGCTGCGGGTGCGCCTCGCCTCCCACCTGCGCAAGCTGCGTAAGCGGCTCCTCCGCGATGCCGATGACCTGCAGAAGCGCCTGGCAGTGTACCAGGCCGGGGCGAATA

>Seq176 [organism=Homo sapiens] LUAD ApoE ε3ε3

CTCGAGCGGCCGCCTGGTGCAGTACCGCGGCGAGGTGCAGGCCATGCTCGGCCAGAGCACCGAGGAGCTGCGGGTGCGCCTCGCCTCCCACCTGCGCAAGCTGCGTAAGCGGCTCCTCCGCGATGCCGATGACCTGCAGAAGCGCCTGGCAGTGTACCAGGCCGGGGCGAATA

>Seq177 [organism=Homo sapiens] LUAD ApoE ε3ε3

GTCGAGCGGCCGCCTGGTGCAGTACCGCGGCGAGGTGCAGGCCATGCTCGGCCAGAGCACCGAGGAGCTGCGGGTGCGCCTCGCCTCCCACCTGCGCAAGCTGCGTAAGCGGCTCCTCCGCGATGCCGATGACCTGCAGAAGCGCCTGGCAGTGTACCAGGCCGGGGCGAATA

>Seq178 [organism=Homo sapiens] LUAD ApoE ε3ε3

AAGCAGCGGCCGCCTGGTGCAGTACCGCGGCGAGGTGCAGGCCATGCTCGGCCAGAGCACCGAGGAGCTGCGGGTGCGCCTCGCCTCCCACCTGCGCAAGCTGCGTAAGCGGCTCCTCCGCGATGCCGATGACCTGCAGAAGCGCCTGGCAGTGTACCAGGCCGGGGCGAATA

>Seq179 [organism=Homo sapiens] LUAD ApoE ε3ε3

TAGCAGCGGCCGCCTGGTGCAGTACCGCGGCGAGGTGCAGGCCATGCTCGGCCAGAGCACCGAGGAGCTGCGGGTGCGCCTCGCCTCCCACCTGCGCAAGCTGCGTAAGCGGCTCCTCCGCGATGCCGATGACCTGCAGAAGCGCCTGGCAGTGTACCAGGCCGGGGCGAATA

>Seq180 [organism=Homo sapiens] LUAD ApoE ε3ε3

GCGCAGCGGCCGCCTGGTGCAGTACCGCGGCGAGGTGCAGGCCATGCTCGGCCAGAGCACCGAGGAGCTGCGGGTGCGCCTCGCCTCCCACCTGCGCAAGCTGCGTAAGCGGCTCCTCCGCGATGCCGATGACCTGCAGAAGCGCCTGGCAGTGTACCAGGCCGGGGCGAATA

>Seq181 [organism=Homo sapiens] LUAD ApoE ε3ε3

ATGCAGCGGCCGCCTGGTGCAGTACCGCGGCGAGGTGCAGGCCATGCTCGGCCAGAGCACCGAGGAGCTGCGGGTGCGCCTCGCCTCCCACCTGCGCAAGCTGCGTAAGCGGCTCCTCCGCGATGCCGATGACCTGCAGAAGCGCCTGGCAGTGTACCAGGCCGGGGCGAATA

>Seq182 [organism=Homo sapiens] LUAD ApoE ε3ε3

TCGCAGCGGCCGCCTGGTGCAGTACCGCGGCGAGGTGCAGGCCATGCTCGGCCAGAGCACCGAGGAGCTGCGGGTGCGCCTCGCCTCCCACCTGCGCAAGCTGCGTAAGCGGCTCCTCCGCGATGCCGATGACCTGCAGAAGCGCCTGGCAGTGTACCAGGCCGGGGCGAATA

>Seq183 [organism=Homo sapiens] LUAD ApoE ε3ε3

GAGCAGCGGCCGCCTGGTGCAGTACCGCGGCGAGGTGCAGGCCATGCTCGGCCAGAGCACCGAGGAGCTGCGGGTGCGCCTCGCCTCCCACCTGCGCAAGCTGCGTAAGCGGCTCCTCCGCGATGCCGATGACCTGCAGAAGCGCCTGGCAGTGTACCAGGCCGGGGCGAATA

>Seq184 [organism=Homo sapiens] LUAD ApoE ε3ε3

CGGCAGCGGCCGCCTGGTGCAGTACCGCGGCGAGGTGCAGGCCATGCTCGGCCAGAGCACCGAGGAGCTGCGGGTGCGCCTCGCCTCCCACCTGCGCAAGCTGCGTAAGCGGCTCCTCCGCGATGCCGATGACCTGCAGAAGCGCCTGGCAGTGTACCAGGCCGGGGCGAATA

>Seq185 [organism=Homo sapiens] LUAD ApoE ε3ε3

GACGAGCGGCCGCCTGGTGCAGTACCGCGGCGAGGTGCAGGCCATGCTCGGCCAGAGCACCGAGGAGCTGCGGGTGCGCCTCGCCTCCCACCTGCGCAAGCTGCGTAAGCGGCTCCTCCGCGATGCCGATGACCTGCAGAAGCGCCTGGCAGTGTACCAGGCCGGGGCGAATA

>Seq186 [organism=Homo sapiens] LUAD ApoE ε3ε3

CTCGAGCGGCCGCCTGGTGCAGTACCGCGGCGAGGTGCAGGCCATGCTCGGCCAGAGCACCGAGGAGCTGCGGGTGCGCCTCGCCTCCCACCTGCGCAAGCTGCGTAAGCGGCTCCTCCGCGATGCCGATGACCTGCAGAAGCGCCTGGCAGTGTACCAGGCCGGGGCGAATA

>Seq188 [organism=Homo sapiens] LUAD ApoE ε3ε3

CGGCGAGGTTCCTGGTATACGGTCCCGGAATGGCAGGACGTGTGCGGCCGCCTGGTGCAGTACCGCGGCGAGGTGCAGGCCATGCTCGGCCAGAGCACCGAGGAGCTGCGGGTGCGCCTCGCCTCCCACCTGCGCAAGCTGCGTAAGCGGCTCCTCCGCGATGCCGATGACCTGCAGAAGCGCCTGGCAGTGTACCAGGCCGGGGCGAATA

>Seq189 [organism=Homo sapiens] LUAD ApoE ε3ε3

AGGTGCAGGTTCCTGGTATACGGTCCCGGAATGGCAGGACGTGTGCGGCCGCCTGGTGCAGTACCGCGGCGAGGTGCAGGCCATGCTCGGCCAGAGCACCGAGGAGCTGCGGGTGCGCCTCGCCTCCCACCTGCGCAAGCTGCGTAAGCGGCTCCTCCGCGATGCCGATGACCTGCAGAAGCGCCTGGCAGTGTACCAGGCCGGGGCGAATA

1>Seq190 [organism=Homo sapiens] LUAD ApoE ε3ε3

AGGCCATG TTCCTGGTATACGGTCCCGGAATGGCAGGACGTGTGCGGCCGCCTGGTGCAGTACCGCGGCGAGGTGCAGGCCATGCTCGGCCAGAGCACCGAGGAGCTGCGGGTGCGCCTCGCCTCCCACCTGCGCAAGCTGCGTAAGCGGCTCCTCCGCGATGCCGATGACCTGCAGAAGCGCCTGGCAGTGTACCAGGCCGGGGCGAATA

>Seq191 [organism=Homo sapiens] LUAD ApoE ε2ε4

TGCGCGGCCGGCGCGGAATGGAGGACGTGTGCGGCCGCCTGGTGCAGTACCGCGGCGAGGTGCAGGCCATGCTCGGCCAGAGCACCGAGGAGCTGCGGGTGCGCCTCGCCTCCCACCTGCGCAAGCTGCGTAAGCGGCTCCTCCGCGATGCCGATGACCTGCAGAAGCGCCTGGCAGTGTACCAGGCCGGGGCGAAT

>Seq192 [organism=Homo sapiens] LUAD ApoE ε2ε4

AGTCGGGCCGGCGCGGAATGGAGGACGTGTGCGGCCGCCTGGTGCAGTACCGCGGCGAGGTGCAGGCCATGCTCGGCCAGAGCACCGAGGAGCTGCGGGTGCGCCTCGCCTCCCACCTGCGCAAGCTGCGTAAGCGGCTCCTCCGCGATGCCGATGACCTGCAGAAGCGCCTGGCAGTGTACCAGGCCGGGGCGAAT

>Seq193 [organism=Homo sapiens] LUAD ApoE ε2ε4

GACCGGCCGGCGCGGAATGGAGGACGTGTGCGGCCGCCTGGTGCAGTACCGCGGCGAGGTGCAGGCCATGCTCGGCCAGAGCACCGAGGAGCTGCGGGTGCGCCTCGCCTCCCACCTGCGCAAGCTGCGTAAGCGGCTCCTCCGCGATGCCGATGACCTGCAGAAGCGCCTGGCAGTGTACCAGGCCGGGGCGAAT

>Seq194 [organism=Homo sapiens] LUAD ApoE ε2ε5

ACGCGGGCCGGCGCGGAATGGAGGACGTGTGCGGCCGCCTGGTGCAGTACCGCGGCGAGGTGCAGGCCATGCTCGGCCAGAGCACCGAGGAGCTGCGGGTGCGCCTCGCCTCCCACCTGCGCAAGCTGCGTAAGCGGCTCCTCCGCGATGCCGATGACCTGCAGAAGCGCCTGGCAGTGTACCAGGCCGGGGCGAAT

>Seq195 [organism=Homo sapiens] LUAD ApoE ε2ε3

GTACGGCCGGCGCGGAATGGAGGACGTGTGCGGCCGCCTGGTGCAGTACCGCGGCGAGGTGCAGGCCATGCTCGGCCAGAGCACCGAGGAGCTGCGGGTGCGCCTCGCCTCCCACCTGCGCAAGCTGCGTAAGCGGCTCCTCCGCGATGCCGATGACCTGCAGAAGCGCCTGGCAGTGTACCAGGCCGGGGCGAAT

>Seq196 [organism=Homo sapiens] LUAD ApoE ε3ε4

GCGCGGGCCGGCGCGGAATGGAGGACGTGTGCGGCCGCCTGGTGCAGTACCGCGGCGAGGTGCAGGCCATGCTCGGCCAGAGCACCGAGGAGCTGCGGGTGCGCCTCGCCTCCCACCTGCGCAAGCTGCGTAAGCGGCTCCTCCGCGATGCCGATGACCTGCAGAAGCGCCTGGCAGTGTACCAGGCCGGGGCGAAT

>Seq197 [organism=Homo sapiens] LUAD ApoE ε3ε4

TGCGGGGCCGGCGCGGAATGGAGGACGTGTGCGGCCGCCTGGTGCAGTACCGCGGCGAGGTGCAGGCCATGCTCGGCCAGAGCACCGAGGAGCTGCGGGTGCGCCTCGCCTCCCACCTGCGCAAGCTGCGTAAGCGGCTCCTCCGCGATGCCGATGACCTGCAGAAGCGCCTGGCAGTGTACCAGGCCGGGGCGAAT

>Seq198 [organism=Homo sapiens] LUAD ApoE ε3ε4

AGTCGGGCCGGCGCGGAATGGAGGACGTGTGCGGCCGCCTGGTGCAGTACCGCGGCGAGGTGCAGGCCATGCTCGGCCAGAGCACCGAGGAGCTGCGGGTGCGCCTCGCCTCCCACCTGCGCAAGCTGCGTAAGCGGCTCCTCCGCGATGCCGATGACCTGCAGAAGCGCCTGGCAGTGTACCAGGCCGGGGCGAAT

>Seq199 [organism=Homo sapiens] LUAD ApoE ε3ε4

GTCGGGGCGGCGCGGAATGGAGGACGTGTGCGGCCGCCTGGTGCAGTACCGCGGCGAGGTGCAGGCCATGCTCGGCCAGAGCACCGAGGAGCTGCGGGTGCGCCTCGCCTCCCACCTGCGCAAGCTGCGTAAGCGGCTCCTCCGCGATGCCGATGACCTGCAGAAGCGCCTGGCAGTGTACCAGGCCGGGGCGAAT

>Seq200 [organism=Homo sapiens] LUAD ApoE ε4ε4

CAGGCGGCGGGCGCGCGGAATGGAGGACGTGCGCGGCCGCCTGGTGCAGTACCGCGGCGAGGTGCAGGCCATGCTCGGCCAGAGCACCGAGGAGCTGCGGGTGCGCCTCGCCTCCCACCTGCGCAAGCTGCGTAAGCGGCTCCTCCGCGATGCCGATGACCTGCAGAAGCGCCTGGCAGTGTACCAGGCCGGGGCGAAT

Control group

>Seq1 [organism=Homo sapiens] Control ApoE ε2ε2

ATGCTAGCTAGCTACGGCTGGGCGCGGAATGGAGGACGTGTGCGGCCGCCTGGTGCAGTACCGCGGCGAGGTGCAGGCCATGCTCGGCCAGAGCACCGAGGAGCTGCGGGTGCGCCTCGCCTCCCACCTGCGCAAGCTGCGTAAGCGGCTCCTCCGCGATGCCGATGACCTGCAGAAGTGCCTGGCAGTGTACCAGGCCGGGGCGAATAA

>Seq2 [organism=Homo sapiens] Control ApoE ε2ε2

GCTAGCTAGCTAGCCGGCTGGGCGCGGAATGGAGGACGTGTGCGGCCGCCTGGTGCAGTACCGCGGCGAGGTGCAGGCCATGCTCGGCCAGAGCACCGAGGAGCTGCGGGTGCGCCTCGCCTCCCACCTGCGCAAGCTGCGTAAGCGGCTCCTCCGCGATGCCGATGACCTGCAGAAGTGCCTGGCAGTGTACCAGGCCGGGGCGAATAA

>Seq3 [organism=Homo sapiens] Control ApoE ε2ε2

TAGCTAGCTAGCTACGGCTGGGCGCGGAATGGAGGACGTGTGCGGCCGCCTGGTGCAGTACCGCGGCGAGGTGCAGGCCATGCTCGGCCAGAGCACCGAGGAGCTGCGGGTGCGCCTCGCCTCCCACCTGCGCAAGCTGCGTAAGCGGCTCCTCCGCGATGCCGATGACCTGCAGAAGTGCCTGGCAGTGTACCAGGCCGGGGCGAATAA

>Seq4 [organism=Homo sapiens] Control ApoE ε2ε2

AGCTAGCTAGCTAGCGGCTGGGCGCGGAATGGAGGACGTGTGCGGCCGCCTGGTGCAGTACCGCGGCGAGGTGCAGGCCATGCTCGGCCAGAGCACCGAGGAGCTGCGGGTGCGCCTCGCCTCCCACCTGCGCAAGCTGCGTAAGCGGCTCCTCCGCGATGCCGATGACCTGCAGAAGTGCCTGGCAGTGTACCAGGCCGGGGCGAATAA

>Seq5 [organism=Homo sapiens] Control ApoE ε2ε2

GCTAGCTAGCTAGCCGGCTGGGCGCGGAATGGAGGACGTGTGCGGCCGCCTGGTGCAGTACCGCGGCGAGGTGCAGGCCATGCTCGGCCAGAGCACCGAGGAGCTGCGGGTGCGCCTCGCCTCCCACCTGCGCAAGCTGCGTAAGCGGCTCCTCCGCGATGCCGATGACCTGCAGAAGTGCCTGGCAGTGTACCAGGCCGGGGCGAATAA

>Seq6 [organism=Homo sapiens] Control ApoE ε2ε2

TAGCTAGCTAGCTACGGCTGGGCGCGGAATGGAGGACGTGTGCGGCCGCCTGGTGCAGTACCGCGGCGAGGTGCAGGCCATGCTCGGCCAGAGCACCGAGGAGCTGCGGGTGCGCCTCGCCTCCCACCTGCGCAAGCTGCGTAAGCGGCTCCTCCGCGATGCCGATGACCTGCAGAAGTGCCTGGCAGTGTACCAGGCCGGGGCGAATAA

>Seq7 [organism=Homo sapiens] Control ApoE ε2ε2

AGCTAGCTAGCTAGCGGCTGGGCGCGGAATGGAGGACGTGTGCGGCCGCCTGGTGCAGTACCGCGGCGAGGTGCAGGCCATGCTCGGCCAGAGCACCGAGGAGCTGCGGGTGCGCCTCGCCTCCCACCTGCGCAAGCTGCGTAAGCGGCTCCTCCGCGATGCCGATGACCTGCAGAAGTGCCTGGCAGTGTACCAGGCCGGGGCGAATAA

>Seq8 [organism=Homo sapiens] Control ApoE ε2ε2

CTAGCTAGCTAGCTCGGCTGGGCGCGGAATGGAGGACGTGTGCGGCCGCCTGGTGCAGTACCGCGGCGAGGTGCAGGCCATGCTCGGCCAGAGCACCGAGGAGCTGCGGGTGCGCCTCGCCTCCCACCTGCGCAAGCTGCGTAAGCGGCTCCTCCGCGATGCCGATGACCTGCAGAAGTGCCTGGCAGTGTACCAGGCCGGGGCGAATAA

>Seq9 [organism=Homo sapiens] Control ApoE ε2ε2

CAAATCCCCGAAGCGGGCAGACCTAGCGCCCAGCGGCTGGGCGCGGAATGGAGGACGTGTGCGGCCGCCTGGTGCAGTACCGCGGCGAGGTGCAGGCCATGCTCGGCCAGAGCACCGAGGAGCTGCGGGTGCGCCTCGCCTCCCACCTGCGCAAGCTGCGTAAGCGGCTCCTCCGCGATGCCGATGACCTGCAGAAGTGCCTGGCAGTGTACCAGGCCGGGGCGAATGCGAATAGCCTGGACGAGGTGA

>Seq10 [organism=Homo sapiens] Control ApoE ε2ε3

AGGCTCCTGCCCCGAAGCGGGCAGACCTAGCGCCCAGCGGCTGGGCGCGGAATGGAGGACGTGTGCGGCCGCCTGGTGCAGTACCGCGGCGAGGTGCAGGCCATGCTCGGCCAGAGCACCGAGGAGCTGCGGGTGCGCCTCGCCTCCCACCTGCGCAAGCTGCGTAAGCGGCTCCTCCGCGATGCCGATGACCTGCAGAAGTGCCTGGCAGTGTACCAGGCCGGGGCGAATGCGAATAAGTGTACCAGGCCGGGGCGAATA

>Seq11 [organism=Homo sapiens] Control ApoE ε2ε3

TCGCTCCTGCCCCGAAGCGGGCAGACCTAGCGCCCAGCGGCTGGGCGCGGAATGGAGGACGTGTGCGGCCGCCTGGTGCAGTACCGCGGCGAGGTGCAGGCCATGCTCGGCCAGAGCACCGAGGAGCTGCGGGTGCGCCTCGCCTCCCACCTGCGCAAGCTGCGTAAGCGGCTCCTCCGCGATGCCGATGACCTGCAGAAGTGCCTGGCAGTGTACCAGGCCGGGGCGAATGCGAATAAGTGTACCAGGCCGGGGCGAATA

>Seq12 [organism=Homo sapiens] Control ApoE ε2ε3

AGGATCCTGCCCCGAAGCGGGCAGACCTAGCGCCCAGCGGCTGGGCGCGGAATGGAGGACGTGTGCGGCCGCCTGGTGCAGTACCGCGGCGAGGTGCAGGCCATGCTCGGCCAGAGCACCGAGGAGCTGCGGGTGCGCCTCGCCTCCCACCTGCGCAAGCTGCGTAAGCGGCTCCTCCGCGATGCCGATGACCTGCAGAAGTGCCTGGCAGTGTACCAGGCCGGGGCGAATGCGAATAAGTGTACCAGGCCGGGGCGAATA

>Seq13 [organism=Homo sapiens] Control ApoE ε2ε3

TCCCTCCTGCCCCGAAGCGGGCAGACCTAGCGCCCAGCGGCTGGGCGCGGAATGGAGGACGTGTGCGGCCGCCTGGTGCAGTACCGCGGCGAGGTGCAGGCCATGCTCGGCCAGAGCACCGAGGAGCTGCGGGTGCGCCTCGCCTCCCACCTGCGCAAGCTGCGTAAGCGGCTCCTCCGCGATGCCGATGACCTGCAGAAGTGCCTGGCAGTGTACCAGGCCGGGGCGAATGCGAATAAGTGTACCAGGCCGGGGCGAATA

>Seq14 [organism=Homo sapiens] Control ApoE ε2ε3

GGACTCCTGCCCCGAAGCGGGCAGACCTAGCGCCCAGCGGCTGGGCGCGGAATGGAGGACGTGTGCGGCCGCCTGGTGCAGTACCGCGGCGAGGTGCAGGCCATGCTCGGCCAGAGCACCGAGGAGCTGCGGGTGCGCCTCGCCTCCCACCTGCGCAAGCTGCGTAAGCGGCTCCTCCGCGATGCCGATGACCTGCAGAAGTGCCTGGCAGTGTACCAGGCCGGGGCGAATGCGAATAAGTGTACCAGGCCGGGGCGAATA

>Seq15 [organism=Homo sapiens] Control ApoE ε2ε3

CAGATCCTGCCCCGAAGCGGGCAGACCTAGCGCCCAGCGGCTGGGCGCGGAATGGAGGACGTGTGCGGCCGCCTGGTGCAGTACCGCGGCGAGGTGCAGGCCATGCTCGGCCAGAGCACCGAGGAGCTGCGGGTGCGCCTCGCCTCCCACCTGCGCAAGCTGCGTAAGCGGCTCCTCCGCGATGCCGATGACCTGCAGAAGTGCCTGGCAGTGTACCAGGCCGGGGCGAATGCGAATAAGTGTACCAGGCCGGGGCGAATA

>Seq16 [organism=Homo sapiens] Control ApoE ε2ε3

GACATCCTGCCCCGAAGCGGGCAGACCTAGCGCCCAGCGGCTGGGCGCGGAATGGAGGACGTGTGCGGCCGCCTGGTGCAGTACCGCGGCGAGGTGCAGGCCATGCTCGGCCAGAGCACCGAGGAGCTGCGGGTGCGCCTCGCCTCCCACCTGCGCAAGCTGCGTAAGCGGCTCCTCCGCGATGCCGATGACCTGCAGAAGTGCCTGGCAGTGTACCAGGCCGGGGCGAATGCGAATAAGTGTACCAGGCCGGGGCGAATA

>Seq17 [organism=Homo sapiens] Control ApoE ε2ε3

TGACTCCTGCCCCGAAGCGGGCAGACCTAGCGCCCAGCGGCTGGGCGCGGAATGGAGGACGTGTGCGGCCGCCTGGTGCAGTACCGCGGCGAGGTGCAGGCCATGCTCGGCCAGAGCACCGAGGAGCTGCGGGTGCGCCTCGCCTCCCACCTGCGCAAGCTGCGTAAGCGGCTCCTCCGCGATGCCGATGACCTGCAGAAGTGCCTGGCAGTGTACCAGGCCGGGGCGAATGCGAATAAGTGTACCAGGCCGGGGCGAATA

>Seq18 [organism=Homo sapiens] Control ApoE ε2ε3

AGGCTCCTGCCCCGAAGCGGGCAGACCTAGCGCCCAGCGGCTGGGCGCGGAATGGAGGACGTGTGCGGCCGCCTGGTGCAGTACCGCGGCGAGGTGCAGGCCATGCTCGGCCAGAGCACCGAGGAGCTGCGGGTGCGCCTCGCCTCCCACCTGCGCAAGCTGCGTAAGCGGCTCCTCCGCGATGCCGATGACCTGCAGAAGTGCCTGGCAGTGTACCAGGCCGGGGCGAATGCGAATAAGTGTACCAGGCCGGGGCGAATA

>Seq19 [organism=Homo sapiens] Control ApoE ε2ε3

TCGGTCCTGCCCCGAAGCGGGCAGACCTAGCGCCCAGCGGCTGGGCGCGGAATGGAGGACGTGTGCGGCCGCCTGGTGCAGTACCGCGGCGAGGTGCAGGCCATGCTCGGCCAGAGCACCGAGGAGCTGCGGGTGCGCCTCGCCTCCCACCTGCGCAAGCTGCGTAAGCGGCTCCTCCGCGATGCCGATGACCTGCAGAAGTGCCTGGCAGTGTACCAGGCCGGGGCGAATGCGAATAAGTGTACCAGGCCGGGGCGAATA

>Seq20 [organism=Homo sapiens] Control ApoE ε2ε3

AGCATCCTGCCCCGAAGCGGGCAGACCTAGCGCCCAGCGGCTGGGCGCGGAATGGAGGACGTGTGCGGCCGCCTGGTGCAGTACCGCGGCGAGGTGCAGGCCATGCTCGGCCAGAGCACCGAGGAGCTGCGGGTGCGCCTCGCCTCCCACCTGCGCAAGCTGCGTAAGCGGCTCCTCCGCGATGCCGATGACCTGCAGAAGTGCCTGGCAGTGTACCAGGCCGGGGCGAATGCGAATAAGTGTACCAGGCCGGGGCGAATA

>Seq21 [organism=Homo sapiens] Control ApoE ε2ε3

GGGCTCCTGCCCCGAAGCGGGCAGACCTAGCGCCCAGCGGCTGGGCGCGGAATGGAGGACGTGTGCGGCCGCCTGGTGCAGTACCGCGGCGAGGTGCAGGCCATGCTCGGCCAGAGCACCGAGGAGCTGCGGGTGCGCCTCGCCTCCCACCTGCGCAAGCTGCGTAAGCGGCTCCTCCGCGATGCCGATGACCTGCAGAAGTGCCTGGCAGTGTACCAGGCCGGGGCGAATGCGAATAAGTGTACCAGGCCGGGGCGAATA

>Seq22 [organism=Homo sapiens] Control ApoE ε2ε3

ACGCTCCTGCCCCGAAGCGGGCAGACCTAGCGCCCAGCGGCTGGGCGCGGAATGGAGGACGTGTGCGGCCGCCTGGTGCAGTACCGCGGCGAGGTGCAGGCCATGCTCGGCCAGAGCACCGAGGAGCTGCGGGTGCGCCTCGCCTCCCACCTGCGCAAGCTGCGTAAGCGGCTCCTCCGCGATGCCGATGACCTGCAGAAGTGCCTGGCAGTGTACCAGGCCGGGGCGAATGCGAATAAGTGTACCAGGCCGGGGCGAATA

>Seq23 [organism=Homo sapiens] Control ApoE ε2ε3

GTCATCCTGCCCCGAAGCGGGCAGACCTAGCGCCCAGCGGCTGGGCGCGGAATGGAGGACGTGTGCGGCCGCCTGGTGCAGTACCGCGGCGAGGTGCAGGCCATGCTCGGCCAGAGCACCGAGGAGCTGCGGGTGCGCCTCGCCTCCCACCTGCGCAAGCTGCGTAAGCGGCTCCTCCGCGATGCCGATGACCTGCAGAAGTGCCTGGCAGTGTACCAGGCCGGGGCGAATGCGAATAAGTGTACCAGGCCGGGGCGAATA

>Seq24 [organism=Homo sapiens] Control ApoE ε2ε3

TACCTCCTGCCCCGAAGCGGGCAGACCTAGCGCCCAGCGGCTGGGCGCGGAATGGAGGACGTGTGCGGCCGCCTGGTGCAGTACCGCGGCGAGGTGCAGGCCATGCTCGGCCAGAGCACCGAGGAGCTGCGGGTGCGCCTCGCCTCCCACCTGCGCAAGCTGCGTAAGCGGCTCCTCCGCGATGCCGATGACCTGCAGAAGTGCCTGGCAGTGTACCAGGCCGGGGCGAATGCGAATAAGTGTACCAGGCCGGGGCGAATA

>Seq25 [organism=Homo sapiens] Control ApoE ε2ε3

AGTCTCCTGCCCCGAAGCGGGCAGACCTAGCGCCCAGCGGCTGGGCGCGGAATGGAGGACGTGTGCGGCCGCCTGGTGCAGTACCGCGGCGAGGTGCAGGCCATGCTCGGCCAGAGCACCGAGGAGCTGCGGGTGCGCCTCGCCTCCCACCTGCGCAAGCTGCGTAAGCGGCTCCTCCGCGATGCCGATGACCTGCAGAAGTGCCTGGCAGTGTACCAGGCCGGGGCGAATGCGAATAAGTGTACCAGGCCGGGGCGAATA

>Seq26 [organism=Homo sapiens] Control ApoE ε2ε3

GACCTCCTGCCCCGAAGCGGGCAGACCTAGCGCCCAGCGGCTGGGCGCGGAATGGAGGACGTGTGCGGCCGCCTGGTGCAGTACCGCGGCGAGGTGCAGGCCATGCTCGGCCAGAGCACCGAGGAGCTGCGGGTGCGCCTCGCCTCCCACCTGCGCAAGCTGCGTAAGCGGCTCCTCCGCGATGCCGATGACCTGCAGAAGTGCCTGGCAGTGTACCAGGCCGGGGCGAATGCGAATAAGTGTACCAGGCCGGGGCGAATA

27>Seq27 [organism=Homo sapiens] Control ApoE ε3ε3

TACGTAGCGGGGCGCGGAATGGAGGACGTGTGCGGCCGCCTGGTGCAGTACCGCGGCGAGGTGCAGGCCATGCTCGGCCAGAGCACCGAGGAGCTGCGGGTGCGCCTCGCCTCCCACCTGCGCAAGCTGCGTAAGCGGCTCCTCCGCGATGCCGATGACCTGCAGAAGCGCCTGGCAGTGTACCAGGCCGGGGCGAAT

28>Seq28 [organism=Homo sapiens] Control ApoE ε3ε3

AGCCTAGCGGGGCGCGGAATGGAGGACGTGTGCGGCCGCCTGGTGCAGTACCGCGGCGAGGTGCAGGCCATGCTCGGCCAGAGCACCGAGGAGCTGCGGGTGCGCCTCGCCTCCCACCTGCGCAAGCTGCGTAAGCGGCTCCTCCGCGATGCCGATGACCTGCAGAAGCGCCTGGCAGTGTACCAGGCCGGGGCGAAT

29>Seq29 [organism=Homo sapiens] Control ApoE ε3ε3

GCGTAGCGGGGCGCGGAATGGAGGACGTGTGCGGCCGCCTGGTGCAGTACCGCGGCGAGGTGCAGGCCATGCTCGGCCAGAGCACCGAGGAGCTGCGGGTGCGCCTCGCCTCCCACCTGCGCAAGCTGCGTAAGCGGCTCCTCCGCGATGCCGATGACCTGCAGAAGCGCCTGGCAGTGTACCAGGCCGGGGCGAAT

30>Seq30 [organism=Homo sapiens] Control ApoE ε3ε3

AACGTAGCGGGGCGCGGAATGGAGGACGTGTGCGGCCGCCTGGTGCAGTACCGCGGCGAGGTGCAGGCCATGCTCGGCCAGAGCACCGAGGAGCTGCGGGTGCGCCTCGCCTCCCACCTGCGCAAGCTGCGTAAGCGGCTCCTCCGCGATGCCGATGACCTGCAGAAGCGCCTGGCAGTGTACCAGGCCGGGGCGAAT

>Seq31 [organism=Homo sapiens] Control ApoE ε3ε3

TCCGTAGCGGGGCGCGGAATGGAGGACGTGTGCGGCCGCCTGGTGCAGTACCGCGGCGAGGTGCAGGCCATGCTCGGCCAGAGCACCGAGGAGCTGCGGGTGCGCCTCGCCTCCCACCTGCGCAAGCTGCGTAAGCGGCTCCTCCGCGATGCCGATGACCTGCAGAAGCGCCTGGCAGTGTACCAGGCCGGGGCGAAT

>Seq32 [organism=Homo sapiens] Control ApoE ε3ε3

GTTGTAGCGGGGCGCGGAATGGAGGACGTGTGCGGCCGCCTGGTGCAGTACCGCGGCGAGGTGCAGGCCATGCTCGGCCAGAGCACCGAGGAGCTGCGGGTGCGCCTCGCCTCCCACCTGCGCAAGCTGCGTAAGCGGCTCCTCCGCGATGCCGATGACCTGCAGAAGCGCCTGGCAGTGTACCAGGCCGGGGCGAAT

>Seq33 [organism=Homo sapiens] Control ApoE ε3ε3

TGCCTAGCGGGGCGCGGAATGGAGGACGTGTGCGGCCGCCTGGTGCAGTACCGCGGCGAGGTGCAGGCCATGCTCGGCCAGAGCACCGAGGAGCTGCGGGTGCGCCTCGCCTCCCACCTGCGCAAGCTGCGTAAGCGGCTCCTCCGCGATGCCGATGACCTGCAGAAGCGCCTGGCAGTGTACCAGGCCGGGGCGAAT

>Seq34 [organism=Homo sapiens] Control ApoE ε3ε3

CGAATAGCGGGGCGCGGAATGGAGGACGTGTGCGGCCGCCTGGTGCAGTACCGCGGCGAGGTGCAGGCCATGCTCGGCCAGAGCACCGAGGAGCTGCGGGTGCGCCTCGCCTCCCACCTGCGCAAGCTGCGTAAGCGGCTCCTCCGCGATGCCGATGACCTGCAGAAGCGCCTGGCAGTGTACCAGGCCGGGGCGAAT

>Seq35 [organism=Homo sapiens] Control ApoE ε3ε3

TCGATAGCGGGGCGCGGAATGGAGGACGTGTGCGGCCGCCTGGTGCAGTACCGCGGCGAGGTGCAGGCCATGCTCGGCCAGAGCACCGAGGAGCTGCGGGTGCGCCTCGCCTCCCACCTGCGCAAGCTGCGTAAGCGGCTCCTCCGCGATGCCGATGACCTGCAGAAGCGCCTGGCAGTGTACCAGGCCGGGGCGAAT

>Seq36 [organism=Homo sapiens] Control ApoE ε3ε3

AGTGTAGCGGGGCGCGGAATGGAGGACGTGTGCGGCCGCCTGGTGCAGTACCGCGGCGAGGTGCAGGCCATGCTCGGCCAGAGCACCGAGGAGCTGCGGGTGCGCCTCGCCTCCCACCTGCGCAAGCTGCGTAAGCGGCTCCTCCGCGATGCCGATGACCTGCAGAAGCGCCTGGCAGTGTACCAGGCCGGGGCGAAT

>Seq37 [organism=Homo sapiens] Control ApoE ε3

ACGATACCGGGCATGTCGCTGTGGGCGCGGAATGGCAGGACGTGTGCGGCCGCCTGGTGCAGTACCGGGCGCGGAATGGAGGACGTGTGCGGCCGCCTGGTGCAGTACCGCGGCGAGGTGCAGGCCATGCTCGGCCAGAGCACCGAGGAGCTGCGGGTGCGCCTCGCCTCCCACCTGCGCAAGCTGCGTAAGCGGCTCCTCCGCGATGCCGATGACCTGCAGAAGCGCCTGGCAGTGTACCAGGCCGGGGCGAAT

>Seq38 [organism=Homo sapiens] Control ApoE ε3ε3

ACGATAGCGGGGCGCGGAATGGAGGACGTGTGCGGCCGCCTGGTGCAGTACCGCGGCGAGGTGCAGGCCATGCTCGGCCAGAGCACCGAGGAGCTGCGGGTGCGCCTCGCCTCCCACCTGCGCAAGCTGCGTAAGCGGCTCCTCCGCGATGCCGATGACCTGCAGAAGCGCCTGGCAGTGTACCAGGCCGGGGCGAAT

>Seq39 [organism=Homo sapiens] Control ApoE ε3ε3

CGTGTAGCGGGGCGCGGAATGGAGGACGTGTGCGGCCGCCTGGTGCAGTACCGCGGCGAGGTGCAGGCCATGCTCGGCCAGAGCACCGAGGAGCTGCGGGTGCGCCTCGCCTCCCACCTGCGCAAGCTGCGTAAGCGGCTCCTCCGCGATGCCGATGACCTGCAGAAGCGCCTGGCAGTGTACCAGGCCGGGGCGAAT

>Seq40 [organism=Homo sapiens] Control ApoE ε3ε3

TGCGTAGCGGGGCGCGGAATGGAGGACGTGTGCGGCCGCCTGGTGCAGTACCGCGGCGAGGTGCAGGCCATGCTCGGCCAGAGCACCGAGGAGCTGCGGGTGCGCCTCGCCTCCCACCTGCGCAAGCTGCGTAAGCGGCTCCTCCGCGATGCCGATGACCTGCAGAAGCGCCTGGCAGTGTACCAGGCCGGGGCGAAT

>Seq41 [organism=Homo sapiens] Control ApoE ε3ε3

TGTGTAGCGGGGCGCGGAATGGAGGACGTGTGCGGCCGCCTGGTGCAGTACCGCGGCGAGGTGCAGGCCATGCTCGGCCAGAGCACCGAGGAGCTGCGGGTGCGCCTCGCCTCCCACCTGCGCAAGCTGCGTAAGCGGCTCCTCCGCGATGCCGATGACCTGCAGAAGCGCCTGGCAGTGTACCAGGCCGGGGCGAAT

>Seq42 [organism=Homo sapiens] Control ApoE ε3ε3

AGTATAGCGGGGCGCGGAATGGAGGACGTGTGCGGCCGCCTGGTGCAGTACCGCGGCGAGGTGCAGGCCATGCTCGGCCAGAGCACCGAGGAGCTGCGGGTGCGCCTCGCCTCCCACCTGCGCAAGCTGCGTAAGCGGCTCCTCCGCGATGCCGATGACCTGCAGAAGCGCCTGGCAGTGTACCAGGCCGGGGCGAAT

>Seq43 [organism=Homo sapiens] Control ApoE ε3ε3

CGTATAGCGGGGCGCGGAATGGAGGACGTGTGCGGCCGCCTGGTGCAGTACCGCGGCGAGGTGCAGGCCATGCTCGGCCAGAGCACCGAGGAGCTGCGGGTGCGCCTCGCCTCCCACCTGCGCAAGCTGCGTAAGCGGCTCCTCCGCGATGCCGATGACCTGCAGAAGCGCCTGGCAGTGTACCAGGCCGGGGCGAAT

>Seq44 [organism=Homo sapiens] Control ApoE ε3ε3

AGCGTAGCGGGGCGCGGAATGGAGGACGTGTGCGGCCGCCTGGTGCAGTACCGCGGCGAGGTGCAGGCCATGCTCGGCCAGAGCACCGAGGAGCTGCGGGTGCGCCTCGCCTCCCACCTGCGCAAGCTGCGTAAGCGGCTCCTCCGCGATGCCGATGACCTGCAGAAGCGCCTGGCAGTGTACCAGGCCGGGGCGAAT

45.>Seq45 [organism=Homo sapiens] Control ApoE ε3ε3

AGCGCAGCGGGGCGCGGAATGGAGGACGTGTGCGGCCGCCTGGTGCAGTACCGCGGCGAGGTGCAGGCCATGCTCGGCCAGAGCACCGAGGAGCTGCGGGTGCGCCTCGCCTCCCACCTGCGCAAGCTGCGTAAGCGGCTCCTCCGCGATGCCGATGACCTGCAGAAGCGCCTGGCAGTGTACCAGGCCGGGGCGAAT

46.>Seq46 [organism=Homo sapiens] Control ApoE ε3ε3

TGAGTAGCGGGGCGCGGAATGGAGGACGTGTGCGGCCGCCTGGTGCAGTACCGCGGCGAGGTGCAGGCCATGCTCGGCCAGAGCACCGAGGAGCTGCGGGTGCGCCTCGCCTCCCACCTGCGCAAGCTGCGTAAGCGGCTCCTCCGCGATGCCGATGACCTGCAGAAGCGCCTGGCAGTGTACCAGGCCGGGGCGAAT

47.>Seq47 [organism=Homo sapiens] Control ApoE ε3ε3

TGCGTAGCGGGGCGCGGAATGGAGGACGTGTGCGGCCGCCTGGTGCAGTACCGCGGCGAGGTGCAGGCCATGCTCGGCCAGAGCACCGAGGAGCTGCGGGTGCGCCTCGCCTCCCACCTGCGCAAGCTGCGTAAGCGGCTCCTCCGCGATGCCGATGACCTGCAGAAGCGCCTGGCAGTGTACCAGGCCGGGGCGAAT

48.>Seq48 [organism=Homo sapiens] Control ApoE ε3ε3

TGTCTAGCGGGGCGCGGAATGGAGGACGTGTGCGGCCGCCTGGTGCAGTACCGCGGCGAGGTGCAGGCCATGCTCGGCCAGAGCACCGAGGAGCTGCGGGTGCGCCTCGCCTCCCACCTGCGCAAGCTGCGTAAGCGGCTCCTCCGCGATGCCGATGACCTGCAGAAGCGCCTGGCAGTGTACCAGGCCGGGGCGAAT

49.>Seq49 [organism=Homo sapiens] Control ApoE ε3ε3

TGCTTAGCGGGGCGCGGAATGGAGGACGTGTGCGGCCGCCTGGTGCAGTACCGCGGCGAGGTGCAGGCCATGCTCGGCCAGAGCACCGAGGAGCTGCGGGTGCGCCTCGCCTCCCACCTGCGCAAGCTGCGTAAGCGGCTCCTCCGCGATGCCGATGACCTGCAGAAGCGCCTGGCAGTGTACCAGGCCGGGGCGAAT

50.>Seq50 [organism=Homo sapiens] Control ApoE ε3ε3

ACGTTAGCGGGGCGCGGAATGGAGGACGTGTGCGGCCGCCTGGTGCAGTACCGCGGCGAGGTGCAGGCCATGCTCGGCCAGAGCACCGAGGAGCTGCGGGTGCGCCTCGCCTCCCACCTGCGCAAGCTGCGTAAGCGGCTCCTCCGCGATGCCGATGACCTGCAGAAGCGCCTGGCAGTGTACCAGGCCGGGGCGAAT

51.>Seq51 [organism=Homo sapiens] Control ApoE ε3ε3

CGTTTAGCGGGGCGCGGAATGGAGGACGTGTGCGGCCGCCTGGTGCAGTACCGCGGCGAGGTGCAGGCCATGCTCGGCCAGAGCACCGAGGAGCTGCGGGTGCGCCTCGCCTCCCACCTGCGCAAGCTGCGTAAGCGGCTCCTCCGCGATGCCGATGACCTGCAGAAGCGCCTGGCAGTGTACCAGGCCGGGGCGAAT

52.>Seq52 [organism=Homo sapiens] Control ApoE ε3ε3

GCTGTAGCGGCCATGTCGCTGTGGGCGCGGAATGGCAGGACGTGTGCGGCCGCCTGGTGCAGTACCGCGGCGAGGTGC

>Seq53 [organism=Homo sapiens] Control ApoE ε3ε3 ATCGTAGCGGGGCGCGGAATGGAGGACGTGTGCGGCCGCCTGGTGCAGTACCGCGGCGAGGTGCAGGCCATGCTCGGCCAGAGCACCGAGGAGCTGCGGGTGCGCCTCGCCTCCCACCTGCGCAAGCTGCGTAAGCGGCTCCTCCGCGATGCCGATGACCTGCAGAAGCGCCTGGCAGTGTACCAGGCCGGGGCGAAT

>Seq54 [organism=Homo sapiens] Control ApoE ε3ε3 GACCTAGCGGGGCGCGGAATGGAGGACGTGTGCGGCCGCCTGGTGCAGTACCGCGGCGAGGTGCAGGCCATGCTCGGCCAGAGCACCGAGGAGCTGCGGGTGCGCCTCGCCTCCCACCTGCGCAAGCTGCGTAAGCGGCTCCTCCGCGATGCCGATGACCTGCAGAAGCGCCTGGCAGTGTACCAGGCCGGGGCGAAT

>Seq55 [organism=Homo sapiens] Control ApoE ε3ε3 GACGTTCGGGGGCGCGGAATGGAGGACGTGTGCGGCCGCCTGGTGCAGTACCGCGGCGAGGTGCAGGCCATGCTCGGCCAGAGCACCGAGGAGCTGCGGGTGCGCCTCGCCTCCCACCTGCGCAAGCTGCGTAAGCGGCTCCTCCGCGATGCCGATGACCTGCAGAAGCGCCTGGCAGTGTACCAGGCCGGGGCGAAT

>Seq56 [organism=Homo sapiens] Control ApoE ε3ε3 GACGTAGTGGGGCGCGGAATGGAGGACGTGTGCGGCCGCCTGGTGCAGTACCGCGGCGAGGTGCAGGCCATGCTCGGCCAGAGCACCGAGGAGCTGCGGGTGCGCCTCGCCTCCCACCTGCGCAAGCTGCGTAAGCGGCTCCTCCGCGATGCCGATGACCTGCAGAAGCGCCTGGCAGTGTACCAGGCCGGGGCGAAT

>Seq57 [organism=Homo sapiens] Control ApoE ε3ε3 GACGTAGCGGGGCGCGGAATGGAGGACGTGTGCGGCCGCCTGGTGCAGTACCGCGGCGAGGTGCAGGCCATGCTCGGCCAGAGCACCGAGGAGCTGCGGGTGCGCCTCGCCTCCCACCTGCGCAAGCTGCGTAAGCGGCTCCTCCGCGATGCCGATGACCTGCAGAAGCGCCTGGCAGTGTACCAGGCCGGGGCGAAT

>Seq58 [organism=Homo sapiens] Control ApoE ε3ε3 GTCGTAGCGGGGCGCGGAATGGAGGACGTGTGCGGCCGCCTGGTGCAGTACCGCGGCGAGGTGCAGGCCATGCTCGGCCAGAGCACCGAGGAGCTGCGGGTGCGCCTCGCCTCCCACCTGCGCAAGCTGCGTAAGCGGCTCCTCCGCGATGCCGATGACCTGCAGAAGCGCCTGGCAGTGTACCAGGCCGGGGCGAAT

>Seq59 [organism=Homo sapiens] Control ApoE ε3ε3 GACATAGCGGGGCGCGGAATGGAGGACGTGTGCGGCCGCCTGGTGCAGTACCGCGGCGAGGTGCAGGCCATGCTCGGCCAGAGCACCGAGGAGCTGCGGGTGCGCCTCGCCTCCCACCTGCGCAAGCTGCGTAAGCGGCTCCTCCGCGATGCCGATGACCTGCAGAAGCGCCTGGCAGTGTACCAGGCCGGGGCGAAT

>Seq60 [organism=Homo sapiens] Control ApoE ε3ε3 GACGGAGCGGGGCGCGGAATGGAGGACGTGTGCGGCCGCCTGGTGCAGTACCGCGGCGAGGTGCAGGCCATGCTCGGCCAGAGCACCGAGGAGCTGCGGGTGCGCCTCGCCTCCCACCTGCGCAAGCTGCGTAAGCGGCTCCTCCGCGATGCCGATGACCTGCAGAAGCGCCTGGCAGTGTACCAGGCCGGGGCGAAT

>Seq61 [organism=Homo sapiens] Control ApoE ε3ε3 GACGTAGGGGGGCGCGGAATGGAGGACGTGTGCGGCCGCCTGGTGCAGTACCGCGGCGAGGTGCAGGCCATGCTCGGCCAGAGCACCGAGGAGCTGCGGGTGCGCCTCGCCTCCCACCTGCGCAAGCTGCGTAAGCGGCTCCTCCGCGATGCCGATGACCTGCAGAAGCGCCTGGCAGTGTACCAGGCCGGGGCGAAT

>Seq62 [organism=Homo sapiens] Control ApoE ε3ε3 GACGTAGTGGGGCGCGGAATGGAGGACGTGTGCGGCCGCCTGGTGCAGTACCGCGGCGAGGTGCAGGCCATGCTCGGCCAGAGCACCGAGGAGCTGCGGGTGCGCCTCGCCTCCCACCTGCGCAAGCTGCGTAAGCGGCTCCTCCGCGATGCCGATGACCTGCAGAAGCGCCTGGCAGTGTACCAGGCCGGGGCGAAT

>Seq63 [organism=Homo sapiens] Control ApoE ε3ε3 GACGTAGCGGGGCGCGGAATGGAGGACGTGTGCGGCCGCCTGGTGCAGTACCGCGGCGAGGTGCAGGCCATGCTCGGCCAGAGCACCGAGGAGCTGCGGGTGCGCCTCGCCTCCCACCTGCGCAAGCTGCGTAAGCGGCTCCTCCGCGATGCCGATGACCTGCAGAAGCGCCTGGCAGTGTACCAGGCCGGGGCGAAT

>Seq64 [organism=Homo sapiens] Control ApoE ε3ε3 GACGTAGTGGGGCGCGGAATGGAGGACGTGTGCGGCCGCCTGGTGCAGTACCGCGGCGAGGTGCAGGCCATGCTCGGCCAGAGCACCGAGGAGCTGCGGGTGCGCCTCGCCTCCCACCTGCGCAAGCTGCGTAAGCGGCTCCTCCGCGATGCCGATGACCTGCAGAAGCGCCTGGCAGTGTACCAGGCCGGGGCGAAT

>Seq65 [organism=Homo sapiens] Control ApoE ε3ε3 GACGTAGCGGGGCGCGGAATGGAGGACGTGTGCGGCCGCCTGGTGCAGTACCGCGGCGAGGTGCAGGCCATGCTCGGCCAGAGCACCGAGGAGCTGCGGGTGCGCCTCGCCTCCCACCTGCGCAAGCTGCGTAAGCGGCTCCTCCGCGATGCCGATGACCTGCAGAAGCGCCTGGCAGTGTACCAGGCCGGGGCGAAT

>Seq66 [organism=Homo sapiens] Control ApoE ε3ε3 GACGTAGGGGGGCGCGGAATGGAGGACGTGTGCGGCCGCCTGGTGCAGTACCGCGGCGAGGTGCAGGCCATGCTCGGCCAGAGCACCGAGGAGCTGCGGGTGCGCCTCGCCTCCCACCTGCGCAAGCTGCGTAAGCGGCTCCTCCGCGATGCCGATGACCTGCAGAAGCGCCTGGCAGTGTACCAGGCCGGGGCGAAT

>Seq67 [organism=Homo sapiens] Control ApoE ε3ε3 GACGTAGCGGGGCGCGGAATGGAGGACGTGTGCGGCCGCCTGGTGCAGTACCGCGGCGAGGTGCAGGCCATGCTCGGCCAGAGCACCGAGGAGCTGCGGGTGCGCCTCGCCTCCCACCTGCGCAAGCTGCGTAAGCGGCTCCTCCGCGATGCCGATGACCTGCAGAAGCGCCTGGCAGTGTACCAGGCCGGGGCGAAT

>Seq68 [organism=Homo sapiens] Control ApoE ε3ε3 GACGTAGTGGGGCGCGGAATGGAGGACGTGTGCGGCCGCCTGGTGCAGTACCGCGGCGAGGTGCAGGCCATGCTCGGCCAGAGCACCGAGGAGCTGCGGGTGCGCCTCGCCTCCCACCTGCGCAAGCTGCGTAAGCGGCTCCTCCGCGATGCCGATGACCTGCAGAAGCGCCTGGCAGTGTACCAGGCCGGGGCGAAT

>Seq69 [organism=Homo sapiens] Control ApoE ε3ε3 GACGTAGCGGGGCGCGGAATGGAGGACGTGTGCGGCCGCCTGGTGCAGTACCGCGGCGAGGTGCAGGCCATGCTCGGCCAGAGCACCGAGGAGCTGCGGGTGCGCCTCGCCTCCCACCTGCGCAAGCTGCGTAAGCGGCTCCTCCGCGATGCCGATGACCTGCAGAAGCGCCTGGCAGTGTACCAGGCCGGGGCGAAT

>Seq70 [organism=Homo sapiens] Control ApoE ε3ε3 GACGTAGGGGGGCGCGGAATGGAGGACGTGTGCGGCCGCCTGGTGCAGTACCGCGGCGAGGTGCAGGCCATGCTCGGCCAGAGCACCGAGGAGCTGCGGGTGCGCCTCGCCTCCCACCTGCGCAAGCTGCGTAAGCGGCTCCTCCGCGATGCCGATGACCTGCAGAAGCGCCTGGCAGTGTACCAGGCCGGGGCGAAT

>Seq71 [organism=Homo sapiens] Control ApoE ε3ε3 GACGTAGTGGGGCGCGGAATGGAGGACGTGTGCGGCCGCCTGGTGCAGTACCGCGGCGAGGTGCAGGCCATGCTCGGCCAGAGCACCGAGGAGCTGCGGGTGCGCCTCGCCTCCCACCTGCGCAAGCTGCGTAAGCGGCTCCTCCGCGATGCCGATGACCTGCAGAAGCGCCTGGCAGTGTACCAGGCCGGGGCGAAT

>Seq72 [organism=Homo sapiens] Control ApoE ε3ε3 GACGTAGCGGGGCGCGGAATGGAGGACGTGTGCGGCCGCCTGGTGCAGTACCGCGGCGAGGTGCAGGCCATGCTCGGCCAGAGCACCGAGGAGCTGCGGGTGCGCCTCGCCTCCCACCTGCGCAAGCTGCGTAAGCGGCTCCTCCGCGATGCCGATGACCTGCAGAAGCGCCTGGCAGTGTACCAGGCCGGGGCGAAT

>Seq73 [organism=Homo sapiens] Control ApoE ε3ε3 ATCGTAGCGGGGCGCGGAATGGAGGACGTGTGCGGCCGCCTGGTGCAGTACCGCGGCGAGGTGCAGGCCATGCTCGGCCAGAGCACCGAGGAGCTGCGGGTGCGCCTCGCCTCCCACCTGCGCAAGCTGCGTAAGCGGCTCCTCCGCGATGCCGATGACCTGCAGAAGCGCCTGGCAGTGTACCAGGCCGGGGCGAAT

>Seq74 [organism=Homo sapiens] Control ApoE ε3ε3 GACCTAGCGGGGCGCGGAATGGAGGACGTGTGCGGCCGCCTGGTGCAGTACCGCGGCGAGGTGCAGGCCATGCTCGGCCAGAGCACCGAGGAGCTGCGGGTGCGCCTCGCCTCCCACCTGCGCAAGCTGCGTAAGCGGCTCCTCCGCGATGCCGATGACCTGCAGAAGCGCCTGGCAGTGTACCAGGCCGGGGCGAAT

>Seq75 [organism=Homo sapiens] Control ApoE ε3ε3 GACGTTCGGGGGCGCGGAATGGAGGACGTGTGCGGCCGCCTGGTGCAGTACCGCGGCGAGGTGCAGGCCATGCTCGGCCAGAGCACCGAGGAGCTGCGGGTGCGCCTCGCCTCCCACCTGCGCAAGCTGCGTAAGCGGCTCCTCCGCGATGCCGATGACCTGCAGAAGCGCCTGGCAGTGTACCAGGCCGGGGCGAAT

>Seq76 [organism=Homo sapiens] Control ApoE ε3ε3 GACGTAGTGGGGCGCGGAATGGAGGACGTGTGCGGCCGCCTGGTGCAGTACCGCGGCGAGGTGCAGGCCATGCTCGGCCAGAGCACCGAGGAGCTGCGGGTGCGCCTCGCCTCCCACCTGCGCAAGCTGCGTAAGCGGCTCCTCCGCGATGCCGATGACCTGCAGAAGCGCCTGGCAGTGTACCAGGCCGGGGCGAAT

>Seq77 [organism=Homo sapiens] Control ApoE ε3ε3 GACGTAGCGGGGCGCGGAATGGAGGACGTGTGCGGCCGCCTGGTGCAGTACCGCGGCGAGGTGCAGGCCATGCTCGGCCAGAGCACCGAGGAGCTGCGGGTGCGCCTCGCCTCCCACCTGCGCAAGCTGCGTAAGCGGCTCCTCCGCGATGCCGATGACCTGCAGAAGCGCCTGGCAGTGTACCAGGCCGGGGCGAAT

>Seq78 [organism=Homo sapiens] Control ApoE ε3ε3 GTCGTAGCGGGGCGCGGAATGGAGGACGTGTGCGGCCGCCTGGTGCAGTACCGCGGCGAGGTGCAGGCCATGCTCGGCCAGAGCACCGAGGAGCTGCGGGTGCGCCTCGCCTCCCACCTGCGCAAGCTGCGTAAGCGGCTCCTCCGCGATGCCGATGACCTGCAGAAGCGCCTGGCAGTGTACCAGGCCGGGGCGAAT

>Seq79 [organism=Homo sapiens] Control ApoE ε3ε3 GACATAGCGGGGCGCGGAATGGAGGACGTGTGCGGCCGCCTGGTGCAGTACCGCGGCGAGGTGCAGGCCATGCTCGGCCAGAGCACCGAGGAGCTGCGGGTGCGCCTCGCCTCCCACCTGCGCAAGCTGCGTAAGCGGCTCCTCCGCGATGCCGATGACCTGCAGAAGCGCCTGGCAGTGTACCAGGCCGGGGCGAAT

>Seq80 [organism=Homo sapiens] Control ApoE ε3ε3 GACGGAGCGGGGCGCGGAATGGAGGACGTGTGCGGCCGCCTGGTGCAGTACCGCGGCGAGGTGCAGGCCATGCTCGGCCAGAGCACCGAGGAGCTGCGGGTGCGCCTCGCCTCCCACCTGCGCAAGCTGCGTAAGCGGCTCCTCCGCGATGCCGATGACCTGCAGAAGCGCCTGGCAGTGTACCAGGCCGGGGCGAAT

>Seq81 [organism=Homo sapiens] Control ApoE ε3ε3 GACGTAGGGGGGCGCGGAATGGAGGACGTGTGCGGCCGCCTGGTGCAGTACCGCGGCGAGGTGCAGGCCATGCTCGGCCAGAGCACCGAGGAGCTGCGGGTGCGCCTCGCCTCCCACCTGCGCAAGCTGCGTAAGCGGCTCCTCCGCGATGCCGATGACCTGCAGAAGCGCCTGGCAGTGTACCAGGCCGGGGCGAAT

>Seq82 [organism=Homo sapiens] Control ApoE ε3ε3 GACGTAGTGGGGCGCGGAATGGAGGACGTGTGCGGCCGCCTGGTGCAGTACCGCGGCGAGGTGCAGGCCATGCTCGGCCAGAGCACCGAGGAGCTGCGGGTGCGCCTCGCCTCCCACCTGCGCAAGCTGCGTAAGCGGCTCCTCCGCGATGCCGATGACCTGCAGAAGCGCCTGGCAGTGTACCAGGCCGGGGCGAAT

>Seq83 [organism=Homo sapiens] Control ApoE ε3ε3 GACGTAGCGGGGCGCGGAATGGAGGACGTGTGCGGCCGCCTGGTGCAGTACCGCGGCGAGGTGCAGGCCATGCTCGGCCAGAGCACCGAGGAGCTGCGGGTGCGCCTCGCCTCCCACCTGCGCAAGCTGCGTAAGCGGCTCCTCCGCGATGCCGATGACCTGCAGAAGCGCCTGGCAGTGTACCAGGCCGGGGCGAAT

>Seq84 [organism=Homo sapiens] Control ApoE ε3ε3 GACGTAGTGGGGCGCGGAATGGAGGACGTGTGCGGCCGCCTGGTGCAGTACCGCGGCGAGGTGCAGGCCATGCTCGGCCAGAGCACCGAGGAGCTGCGGGTGCGCCTCGCCTCCCACCTGCGCAAGCTGCGTAAGCGGCTCCTCCGCGATGCCGATGACCTGCAGAAGCGCCTGGCAGTGTACCAGGCCGGGGCGAAT

>Seq85 [organism=Homo sapiens] Control ApoE ε3ε3 GACGTAGCGGGGCGCGGAATGGAGGACGTGTGCGGCCGCCTGGTGCAGTACCGCGGCGAGGTGCAGGCCATGCTCGGCCAGAGCACCGAGGAGCTGCGGGTGCGCCTCGCCTCCCACCTGCGCAAGCTGCGTAAGCGGCTCCTCCGCGATGCCGATGACCTGCAGAAGCGCCTGGCAGTGTACCAGGCCGGGGCGAAT

>Seq86 [organism=Homo sapiens] Control ApoE ε3ε3 GACGTAGGGGGGCGCGGAATGGAGGACGTGTGCGGCCGCCTGGTGCAGTACCGCGGCGAGGTGCAGGCCATGCTCGGCCAGAGCACCGAGGAGCTGCGGGTGCGCCTCGCCTCCCACCTGCGCAAGCTGCGTAAGCGGCTCCTCCGCGATGCCGATGACCTGCAGAAGCGCCTGGCAGTGTACCAGGCCGGGGCGAAT

>Seq87 [organism=Homo sapiens] Control ApoE ε3ε3 GACGTAGCGGGGCGCGGAATGGAGGACGTGTGCGGCCGCCTGGTGCAGTACCGCGGCGAGGTGCAGGCCATGCTCGGCCAGAGCACCGAGGAGCTGCGGGTGCGCCTCGCCTCCCACCTGCGCAAGCTGCGTAAGCGGCTCCTCCGCGATGCCGATGACCTGCAGAAGCGCCTGGCAGTGTACCAGGCCGGGGCGAAT

>Seq88 [organism=Homo sapiens] Control ApoE ε3ε3 GACGTAGTGGGGCGCGGAATGGAGGACGTGTGCGGCCGCCTGGTGCAGTACCGCGGCGAGGTGCAGGCCATGCTCGGCCAGAGCACCGAGGAGCTGCGGGTGCGCCTCGCCTCCCACCTGCGCAAGCTGCGTAAGCGGCTCCTCCGCGATGCCGATGACCTGCAGAAGCGCCTGGCAGTGTACCAGGCCGGGGCGAAT

>Seq89 [organism=Homo sapiens] Control ApoE ε3ε3

GACGTAGCGGGGCGCGGAATGGAGGACGTGTGCGGCCGCCTGGTGCAGTACCGCGGCGAGGTGCAGGCCATGCTCGGCCAGAGCACCGAGGAGCTGCGGGTGCGCCTCGCCTCCCACCTGCGCAAGCTGCGTAAGCGGCTCCTCCGCGATGCCGATGACCTGCAGAAGCGCCTGGCAGTGTACCAGGCCGGGGCGAAT

>Seq90 [organism=Homo sapiens] Control ApoE ε3ε3

GACGTAGGGGGGCGCGGAATGGAGGACGTGTGCGGCCGCCTGGTGCAGTACCGCGGCGAGGTGCAGGCCATGCTCGGCCAGAGCACCGAGGAGCTGCGGGTGCGCCTCGCCTCCCACCTGCGCAAGCTGCGTAAGCGGCTCCTCCGCGATGCCGATGACCTGCAGAAGCGCCTGGCAGTGTACCAGGCCGGGGCGAAT

>Seq91 [organism=Homo sapiens] Control ApoE ε3ε3

GACGTAGTGGGGCGCGGAATGGAGGACGTGTGCGGCCGCCTGGTGCAGTACCGCGGCGAGGTGCAGGCCATGCTCGGCCAGAGCACCGAGGAGCTGCGGGTGCGCCTCGCCTCCCACCTGCGCAAGCTGCGTAAGCGGCTCCTCCGCGATGCCGATGACCTGCAGAAGCGCCTGGCAGTGTACCAGGCCGGGGCGAAT

>Seq92 [organism=Homo sapiens] Control ApoE ε3ε3

GACGTAGCGGGGCGCGGAATGGAGGACGTGTGCGGCCGCCTGGTGCAGTACCGCGGCGAGGTGCAGGCCATGCTCGGCCAGAGCACCGAGGAGCTGCGGGTGCGCCTCGCCTCCCACCTGCGCAAGCTGCGTAAGCGGCTCCTCCGCGATGCCGATGACCTGCAGAAGCGCCTGGCAGTGTACCAGGCCGGGGCGAAT

>Seq93 [organism=Homo sapiens] Control ApoE ε3ε3

ATCGTAGCGGGGCGCGGAATGGAGGACGTGTGCGGCCGCCTGGTGCAGTACCGCGGCGAGGTGCAGGCCATGCTCGGCCAGAGCACCGAGGAGCTGCGGGTGCGCCTCGCCTCCCACCTGCGCAAGCTGCGTAAGCGGCTCCTCCGCGATGCCGATGACCTGCAGAAGCGCCTGGCAGTGTACCAGGCCGGGGCGAAT

>Seq94 [organism=Homo sapiens] Control ApoE ε3ε3

GACCTAGCGGGGCGCGGAATGGAGGACGTGTGCGGCCGCCTGGTGCAGTACCGCGGCGAGGTGCAGGCCATGCTCGGCCAGAGCACCGAGGAGCTGCGGGTGCGCCTCGCCTCCCACCTGCGCAAGCTGCGTAAGCGGCTCCTCCGCGATGCCGATGACCTGCAGAAGCGCCTGGCAGTGTACCAGGCCGGGGCGAAT

>Seq95 [organism=Homo sapiens] Control ApoE ε3ε3

GACGTTCGGGGGCGCGGAATGGAGGACGTGTGCGGCCGCCTGGTGCAGTACCGCGGCGAGGTGCAGGCCATGCTCGGCCAGAGCACCGAGGAGCTGCGGGTGCGCCTCGCCTCCCACCTGCGCAAGCTGCGTAAGCGGCTCCTCCGCGATGCCGATGACCTGCAGAAGCGCCTGGCAGTGTACCAGGCCGGGGCGAAT

>Seq96 [organism=Homo sapiens] Control ApoE ε3ε3

GACGTAGTGGGGCGCGGAATGGAGGACGTGTGCGGCCGCCTGGTGCAGTACCGCGGCGAGGTGCAGGCCATGCTCGGCCAGAGCACCGAGGAGCTGCGGGTGCGCCTCGCCTCCCACCTGCGCAAGCTGCGTAAGCGGCTCCTCCGCGATGCCGATGACCTGCAGAAGCGCCTGGCAGTGTACCAGGCCGGGGCGAAT

>Seq97 [organism=Homo sapiens] Control ApoE ε3ε3

GACGTAGCGGGGCGCGGAATGGAGGACGTGTGCGGCCGCCTGGTGCAGTACCGCGGCGAGGTGCAGGCCATGCTCGGCCAGAGCACCGAGGAGCTGCGGGTGCGCCTCGCCTCCCACCTGCGCAAGCTGCGTAAGCGGCTCCTCCGCGATGCCGATGACCTGCAGAAGCGCCTGGCAGTGTACCAGGCCGGGGCGAAT

>Seq98 [organism=Homo sapiens] Control ApoE ε3ε3

GTCGTAGCGGGGCGCGGAATGGAGGACGTGTGCGGCCGCCTGGTGCAGTACCGCGGCGAGGTGCAGGCCATGCTCGGCCAGAGCACCGAGGAGCTGCGGGTGCGCCTCGCCTCCCACCTGCGCAAGCTGCGTAAGCGGCTCCTCCGCGATGCCGATGACCTGCAGAAGCGCCTGGCAGTGTACCAGGCCGGGGCGAAT

>Seq99 [organism=Homo sapiens] Control ApoE ε3ε3

GACATAGCGGGGCGCGGAATGGAGGACGTGTGCGGCCGCCTGGTGCAGTACCGCGGCGAGGTGCAGGCCATGCTCGGCCAGAGCACCGAGGAGCTGCGGGTGCGCCTCGCCTCCCACCTGCGCAAGCTGCGTAAGCGGCTCCTCCGCGATGCCGATGACCTGCAGAAGCGCCTGGCAGTGTACCAGGCCGGGGCGAAT

>Seq100 [organism=Homo sapiens] Control ApoE ε3ε3

GACGGAGCGGGGCGCGGAATGGAGGACGTGTGCGGCCGCCTGGTGCAGTACCGCGGCGAGGTGCAGGCCATGCTCGGCCAGAGCACCGAGGAGCTGCGGGTGCGCCTCGCCTCCCACCTGCGCAAGCTGCGTAAGCGGCTCCTCCGCGATGCCGATGACCTGCAGAAGCGCCTGGCAGTGTACCAGGCCGGGGCGAAT

>Seq101 [organism=Homo sapiens] Control ApoE ε3ε3

GACGTAGGGGGGCGCGGAATGGAGGACGTGTGCGGCCGCCTGGTGCAGTACCGCGGCGAGGTGCAGGCCATGCTCGGCCAGAGCACCGAGGAGCTGCGGGTGCGCCTCGCCTCCCACCTGCGCAAGCTGCGTAAGCGGCTCCTCCGCGATGCCGATGACCTGCAGAAGCGCCTGGCAGTGTACCAGGCCGGGGCGAAT

>Seq102 [organism=Homo sapiens] Control ApoE ε3ε3

GACGTAGTGGGGCGCGGAATGGAGGACGTGTGCGGCCGCCTGGTGCAGTACCGCGGCGAGGTGCAGGCCATGCTCGGCCAGAGCACCGAGGAGCTGCGGGTGCGCCTCGCCTCCCACCTGCGCAAGCTGCGTAAGCGGCTCCTCCGCGATGCCGATGACCTGCAGAAGCGCCTGGCAGTGTACCAGGCCGGGGCGAAT

>Seq103 [organism=Homo sapiens] Control ApoE ε3ε3

GACGTAGCGGGGCGCGGAATGGAGGACGTGTGCGGCCGCCTGGTGCAGTACCGCGGCGAGGTGCAGGCCATGCTCGGCCAGAGCACCGAGGAGCTGCGGGTGCGCCTCGCCTCCCACCTGCGCAAGCTGCGTAAGCGGCTCCTCCGCGATGCCGATGACCTGCAGAAGCGCCTGGCAGTGTACCAGGCCGGGGCGAAT

>Seq104 [organism=Homo sapiens] Control ApoE ε3ε3

GACGTAGTGGGGCGCGGAATGGAGGACGTGTGCGGCCGCCTGGTGCAGTACCGCGGCGAGGTGCAGGCCATGCTCGGCCAGAGCACCGAGGAGCTGCGGGTGCGCCTCGCCTCCCACCTGCGCAAGCTGCGTAAGCGGCTCCTCCGCGATGCCGATGACCTGCAGAAGCGCCTGGCAGTGTACCAGGCCGGGGCGAAT

>Seq105 [organism=Homo sapiens] Control ApoE ε3ε3

GACGTAGCGGGGCGCGGAATGGAGGACGTGTGCGGCCGCCTGGTGCAGTACCGCGGCGAGGTGCAGGCCATGCTCGGCCAGAGCACCGAGGAGCTGCGGGTGCGCCTCGCCTCCCACCTGCGCAAGCTGCGTAAGCGGCTCCTCCGCGATGCCGATGACCTGCAGAAGCGCCTGGCAGTGTACCAGGCCGGGGCGAAT

>Seq106 [organism=Homo sapiens] Control ApoE ε3ε3

GACGTAGGGGGGCGCGGAATGGAGGACGTGTGCGGCCGCCTGGTGCAGTACCGCGGCGAGGTGCAGGCCATGCTCGGCCAGAGCACCGAGGAGCTGCGGGTGCGCCTCGCCTCCCACCTGCGCAAGCTGCGTAAGCGGCTCCTCCGCGATGCCGATGACCTGCAGAAGCGCCTGGCAGTGTACCAGGCCGGGGCGAAT

>Seq107 [organism=Homo sapiens] Control ApoE ε3ε3

GACGTAGCGGGGCGCGGAATGGAGGACGTGTGCGGCCGCCTGGTGCAGTACCGCGGCGAGGTGCAGGCCATGCTCGGCCAGAGCACCGAGGAGCTGCGGGTGCGCCTCGCCTCCCACCTGCGCAAGCTGCGTAAGCGGCTCCTCCGCGATGCCGATGACCTGCAGAAGCGCCTGGCAGTGTACCAGGCCGGGGCGAAT

>Seq108 [organism=Homo sapiens] Control ApoE ε3ε3

GACGTAGTGGGGCGCGGAATGGAGGACGTGTGCGGCCGCCTGGTGCAGTACCGCGGCGAGGTGCAGGCCATGCTCGGCCAGAGCACCGAGGAGCTGCGGGTGCGCCTCGCCTCCCACCTGCGCAAGCTGCGTAAGCGGCTCCTCCGCGATGCCGATGACCTGCAGAAGCGCCTGGCAGTGTACCAGGCCGGGGCGAAT

>Seq109 [organism=Homo sapiens] Control ApoE ε3ε3

GACGTAGCGGGGCGCGGAATGGAGGACGTGTGCGGCCGCCTGGTGCAGTACCGCGGCGAGGTGCAGGCCATGCTCGGCCAGAGCACCGAGGAGCTGCGGGTGCGCCTCGCCTCCCACCTGCGCAAGCTGCGTAAGCGGCTCCTCCGCGATGCCGATGACCTGCAGAAGCGCCTGGCAGTGTACCAGGCCGGGGCGAAT

>Seq110 [organism=Homo sapiens] Control ApoE ε3ε3

GACGTAGGGGGGCGCGGAATGGAGGACGTGTGCGGCCGCCTGGTGCAGTACCGCGGCGAGGTGCAGGCCATGCTCGGCCAGAGCACCGAGGAGCTGCGGGTGCGCCTCGCCTCCCACCTGCGCAAGCTGCGTAAGCGGCTCCTCCGCGATGCCGATGACCTGCAGAAGCGCCTGGCAGTGTACCAGGCCGGGGCGAAT

>Seq111 [organism=Homo sapiens] Control ApoE ε3ε3

GACGTAGTGGGGCGCGGAATGGAGGACGTGTGCGGCCGCCTGGTGCAGTACCGCGGCGAGGTGCAGGCCATGCTCGGCCAGAGCACCGAGGAGCTGCGGGTGCGCCTCGCCTCCCACCTGCGCAAGCTGCGTAAGCGGCTCCTCCGCGATGCCGATGACCTGCAGAAGCGCCTGGCAGTGTACCAGGCCGGGGCGAAT

>Seq112 [organism=Homo sapiens] Control ApoE ε3ε3

GACGTAGCGGGGCGCGGAATGGAGGACGTGTGCGGCCGCCTGGTGCAGTACCGCGGCGAGGTGCAGGCCATGCTCGGCCAGAGCACCGAGGAGCTGCGGGTGCGCCTCGCCTCCCACCTGCGCAAGCTGCGTAAGCGGCTCCTCCGCGATGCCGATGACCTGCAGAAGCGCCTGGCAGTGTACCAGGCCGGGGCGAAT

>Seq113 [organism=Homo sapiens] Control ApoE ε3ε3

ATCGTAGCGGGGCGCGGAATGGAGGACGTGTGCGGCCGCCTGGTGCAGTACCGCGGCGAGGTGCAGGCCATGCTCGGCCAGAGCACCGAGGAGCTGCGGGTGCGCCTCGCCTCCCACCTGCGCAAGCTGCGTAAGCGGCTCCTCCGCGATGCCGATGACCTGCAGAAGCGCCTGGCAGTGTACCAGGCCGGGGCGAAT

>Seq114 [organism=Homo sapiens] Control ApoE ε3ε3

GACCTAGCGGGGCGCGGAATGGAGGACGTGTGCGGCCGCCTGGTGCAGTACCGCGGCGAGGTGCAGGCCATGCTCGGCCAGAGCACCGAGGAGCTGCGGGTGCGCCTCGCCTCCCACCTGCGCAAGCTGCGTAAGCGGCTCCTCCGCGATGCCGATGACCTGCAGAAGCGCCTGGCAGTGTACCAGGCCGGGGCGAAT

>Seq115 [organism=Homo sapiens] Control ApoE ε3ε3

GACGTTCGGGGGCGCGGAATGGAGGACGTGTGCGGCCGCCTGGTGCAGTACCGCGGCGAGGTGCAGGCCATGCTCGGCCAGAGCACCGAGGAGCTGCGGGTGCGCCTCGCCTCCCACCTGCGCAAGCTGCGTAAGCGGCTCCTCCGCGATGCCGATGACCTGCAGAAGCGCCTGGCAGTGTACCAGGCCGGGGCGAAT

>Seq116 [organism=Homo sapiens] Control ApoE ε3ε3

GACGTAGTGGGGCGCGGAATGGAGGACGTGTGCGGCCGCCTGGTGCAGTACCGCGGCGAGGTGCAGGCCATGCTCGGCCAGAGCACCGAGGAGCTGCGGGTGCGCCTCGCCTCCCACCTGCGCAAGCTGCGTAAGCGGCTCCTCCGCGATGCCGATGACCTGCAGAAGCGCCTGGCAGTGTACCAGGCCGGGGCGAAT

>Seq117 [organism=Homo sapiens] Control ApoE ε3ε3

GACGTAGCGGGGCGCGGAATGGAGGACGTGTGCGGCCGCCTGGTGCAGTACCGCGGCGAGGTGCAGGCCATGCTCGGCCAGAGCACCGAGGAGCTGCGGGTGCGCCTCGCCTCCCACCTGCGCAAGCTGCGTAAGCGGCTCCTCCGCGATGCCGATGACCTGCAGAAGCGCCTGGCAGTGTACCAGGCCGGGGCGAAT

>Seq118 [organism=Homo sapiens] Control ApoE ε3ε3

GTCGTAGCGGGGCGCGGAATGGAGGACGTGTGCGGCCGCCTGGTGCAGTACCGCGGCGAGGTGCAGGCCATGCTCGGCCAGAGCACCGAGGAGCTGCGGGTGCGCCTCGCCTCCCACCTGCGCAAGCTGCGTAAGCGGCTCCTCCGCGATGCCGATGACCTGCAGAAGCGCCTGGCAGTGTACCAGGCCGGGGCGAAT

>Seq119 [organism=Homo sapiens] Control ApoE ε3ε3

GACATAGCGGGGCGCGGAATGGAGGACGTGTGCGGCCGCCTGGTGCAGTACCGCGGCGAGGTGCAGGCCATGCTCGGCCAGAGCACCGAGGAGCTGCGGGTGCGCCTCGCCTCCCACCTGCGCAAGCTGCGTAAGCGGCTCCTCCGCGATGCCGATGACCTGCAGAAGCGCCTGGCAGTGTACCAGGCCGGGGCGAAT

>Seq120 [organism=Homo sapiens] Control ApoE ε3ε3

GACGGAGCGGGGCGCGGAATGGAGGACGTGTGCGGCCGCCTGGTGCAGTACCGCGGCGAGGTGCAGGCCATGCTCGGCCAGAGCACCGAGGAGCTGCGGGTGCGCCTCGCCTCCCACCTGCGCAAGCTGCGTAAGCGGCTCCTCCGCGATGCCGATGACCTGCAGAAGCGCCTGGCAGTGTACCAGGCCGGGGCGAAT

>Seq121 [organism=Homo sapiens] Control ApoE ε3ε3

GACGTAGGGGGGCGCGGAATGGAGGACGTGTGCGGCCGCCTGGTGCAGTACCGCGGCGAGGTGCAGGCCATGCTCGGCCAGAGCACCGAGGAGCTGCGGGTGCGCCTCGCCTCCCACCTGCGCAAGCTGCGTAAGCGGCTCCTCCGCGATGCCGATGACCTGCAGAAGCGCCTGGCAGTGTACCAGGCCGGGGCGAAT

>Seq122 [organism=Homo sapiens] Control ApoE ε3ε3

GACGTAGTGGGGCGCGGAATGGAGGACGTGTGCGGCCGCCTGGTGCAGTACCGCGGCGAGGTGCAGGCCATGCTCGGCCAGAGCACCGAGGAGCTGCGGGTGCGCCTCGCCTCCCACCTGCGCAAGCTGCGTAAGCGGCTCCTCCGCGATGCCGATGACCTGCAGAAGCGCCTGGCAGTGTACCAGGCCGGGGCGAAT

>Seq123 [organism=Homo sapiens] Control ApoE ε3ε3

GACGTAGCGGGGCGCGGAATGGAGGACGTGTGCGGCCGCCTGGTGCAGTACCGCGGCGAGGTGCAGGCCATGCTCGGCCAGAGCACCGAGGAGCTGCGGGTGCGCCTCGCCTCCCACCTGCGCAAGCTGCGTAAGCGGCTCCTCCGCGATGCCGATGACCTGCAGAAGCGCCTGGCAGTGTACCAGGCCGGGGCGAAT

>Seq124 [organism=Homo sapiens] Control ApoE ε3ε3

GACGTAGTGGGGCGCGGAATGGAGGACGTGTGCGGCCGCCTGGTGCAGTACCGCGGCGAGGTGCAGGCCATGCTCGGCCAGAGCACCGAGGAGCTGCGGGTGCGCCTCGCCTCCCACCTGCGCAAGCTGCGTAAGCGGCTCCTCCGCGATGCCGATGACCTGCAGAAGCGCCTGGCAGTGTACCAGGCCGGGGCGAAT

>Seq125 [organism=Homo sapiens] Control ApoE ε3ε3

GACGTAGCGGGGCGCGGAATGGAGGACGTGTGCGGCCGCCTGGTGCAGTACCGCGGCGAGGTGCAGGCCATGCTCGGCCAGAGCACCGAGGAGCTGCGGGTGCGCCTCGCCTCCCACCTGCGCAAGCTGCGTAAGCGGCTCCTCCGCGATGCCGATGACCTGCAGAAGCGCCTGGCAGTGTACCAGGCCGGGGCGAAT

>Seq126 [organism=Homo sapiens] Control ApoE ε3ε3

GACGTAGGGGGGCGCGGAATGGAGGACGTGTGCGGCCGCCTGGTGCAGTACCGCGGCGAGGTGCAGGCCATGCTCGGCCAGAGCACCGAGGAGCTGCGGGTGCGCCTCGCCTCCCACCTGCGCAAGCTGCGTAAGCGGCTCCTCCGCGATGCCGATGACCTGCAGAAGCGCCTGGCAGTGTACCAGGCCGGGGCGAAT

>Seq127 [organism=Homo sapiens] Control ApoE ε3ε3

GACGTAGCGGGGCGCGGAATGGAGGACGTGTGCGGCCGCCTGGTGCAGTACCGCGGCGAGGTGCAGGCCATGCTCGGCCAGAGCACCGAGGAGCTGCGGGTGCGCCTCGCCTCCCACCTGCGCAAGCTGCGTAAGCGGCTCCTCCGCGATGCCGATGACCTGCAGAAGCGCCTGGCAGTGTACCAGGCCGGGGCGAAT

>Seq128 [organism=Homo sapiens] Control ApoE ε3ε3

GACGTAGTGGGGCGCGGAATGGAGGACGTGTGCGGCCGCCTGGTGCAGTACCGCGGCGAGGTGCAGGCCATGCTCGGCCAGAGCACCGAGGAGCTGCGGGTGCGCCTCGCCTCCCACCTGCGCAAGCTGCGTAAGCGGCTCCTCCGCGATGCCGATGACCTGCAGAAGCGCCTGGCAGTGTACCAGGCCGGGGCGAAT

>Seq129 [organism=Homo sapiens] Control ApoE ε3ε3

GACGTAGCGGGGCGCGGAATGGAGGACGTGTGCGGCCGCCTGGTGCAGTACCGCGGCGAGGTGCAGGCCATGCTCGGCCAGAGCACCGAGGAGCTGCGGGTGCGCCTCGCCTCCCACCTGCGCAAGCTGCGTAAGCGGCTCCTCCGCGATGCCGATGACCTGCAGAAGCGCCTGGCAGTGTACCAGGCCGGGGCGAAT

>Seq130 [organism=Homo sapiens] Control ApoE ε3ε3

GACGTAGGGGGGCGCGGAATGGAGGACGTGTGCGGCCGCCTGGTGCAGTACCGCGGCGAGGTGCAGGCCATGCTCGGCCAGAGCACCGAGGAGCTGCGGGTGCGCCTCGCCTCCCACCTGCGCAAGCTGCGTAAGCGGCTCCTCCGCGATGCCGATGACCTGCAGAAGCGCCTGGCAGTGTACCAGGCCGGGGCGAAT

>Seq131 [organism=Homo sapiens] Control ApoE ε3ε3

GACGTAGTGGGGCGCGGAATGGAGGACGTGTGCGGCCGCCTGGTGCAGTACCGCGGCGAGGTGCAGGCCATGCTCGGCCAGAGCACCGAGGAGCTGCGGGTGCGCCTCGCCTCCCACCTGCGCAAGCTGCGTAAGCGGCTCCTCCGCGATGCCGATGACCTGCAGAAGCGCCTGGCAGTGTACCAGGCCGGGGCGAAT

>Seq132 [organism=Homo sapiens] Control ApoE ε3ε3

GACGTAGCGGGGCGCGGAATGGAGGACGTGTGCGGCCGCCTGGTGCAGTACCGCGGCGAGGTGCAGGCCATGCTCGGCCAGAGCACCGAGGAGCTGCGGGTGCGCCTCGCCTCCCACCTGCGCAAGCTGCGTAAGCGGCTCCTCCGCGATGCCGATGACCTGCAGAAGCGCCTGGCAGTGTACCAGGCCGGGGCGAAT

>Seq133 [organism=Homo sapiens] Control ApoE ε3ε3

ATCGTAGCGGGGCGCGGAATGGAGGACGTGTGCGGCCGCCTGGTGCAGTACCGCGGCGAGGTGCAGGCCATGCTCGGCCAGAGCACCGAGGAGCTGCGGGTGCGCCTCGCCTCCCACCTGCGCAAGCTGCGTAAGCGGCTCCTCCGCGATGCCGATGACCTGCAGAAGCGCCTGGCAGTGTACCAGGCCGGGGCGAAT

>Seq134 [organism=Homo sapiens] Control ApoE ε3ε3

GACCTAGCGGGGCGCGGAATGGAGGACGTGTGCGGCCGCCTGGTGCAGTACCGCGGCGAGGTGCAGGCCATGCTCGGCCAGAGCACCGAGGAGCTGCGGGTGCGCCTCGCCTCCCACCTGCGCAAGCTGCGTAAGCGGCTCCTCCGCGATGCCGATGACCTGCAGAAGCGCCTGGCAGTGTACCAGGCCGGGGCGAAT

>Seq135 [organism=Homo sapiens] Control ApoE ε3ε3

GACGTTCGGGGGCGCGGAATGGAGGACGTGTGCGGCCGCCTGGTGCAGTACCGCGGCGAGGTGCAGGCCATGCTCGGCCAGAGCACCGAGGAGCTGCGGGTGCGCCTCGCCTCCCACCTGCGCAAGCTGCGTAAGCGGCTCCTCCGCGATGCCGATGACCTGCAGAAGCGCCTGGCAGTGTACCAGGCCGGGGCGAAT

>Seq136 [organism=Homo sapiens] Control ApoE ε3ε3

GACGTAGTGGGGCGCGGAATGGAGGACGTGTGCGGCCGCCTGGTGCAGTACCGCGGCGAGGTGCAGGCCATGCTCGGCCAGAGCACCGAGGAGCTGCGGGTGCGCCTCGCCTCCCACCTGCGCAAGCTGCGTAAGCGGCTCCTCCGCGATGCCGATGACCTGCAGAAGCGCCTGGCAGTGTACCAGGCCGGGGCGAAT

>Seq137 [organism=Homo sapiens] Control ApoE ε3ε3

GACGTAGCGGGGCGCGGAATGGAGGACGTGTGCGGCCGCCTGGTGCAGTACCGCGGCGAGGTGCAGGCCATGCTCGGCCAGAGCACCGAGGAGCTGCGGGTGCGCCTCGCCTCCCACCTGCGCAAGCTGCGTAAGCGGCTCCTCCGCGATGCCGATGACCTGCAGAAGCGCCTGGCAGTGTACCAGGCCGGGGCGAAT

>Seq138 [organism=Homo sapiens] Control ApoE ε3ε3

GTCGTAGCGGGGCGCGGAATGGAGGACGTGTGCGGCCGCCTGGTGCAGTACCGCGGCGAGGTGCAGGCCATGCTCGGCCAGAGCACCGAGGAGCTGCGGGTGCGCCTCGCCTCCCACCTGCGCAAGCTGCGTAAGCGGCTCCTCCGCGATGCCGATGACCTGCAGAAGCGCCTGGCAGTGTACCAGGCCGGGGCGAAT

>Seq139 [organism=Homo sapiens] Control ApoE ε3ε3

GACATAGCGGGGCGCGGAATGGAGGACGTGTGCGGCCGCCTGGTGCAGTACCGCGGCGAGGTGCAGGCCATGCTCGGCCAGAGCACCGAGGAGCTGCGGGTGCGCCTCGCCTCCCACCTGCGCAAGCTGCGTAAGCGGCTCCTCCGCGATGCCGATGACCTGCAGAAGCGCCTGGCAGTGTACCAGGCCGGGGCGAAT

>Seq140 [organism=Homo sapiens] Control ApoE ε3ε3

GACGGAGCGGGGCGCGGAATGGAGGACGTGTGCGGCCGCCTGGTGCAGTACCGCGGCGAGGTGCAGGCCATGCTCGGCCAGAGCACCGAGGAGCTGCGGGTGCGCCTCGCCTCCCACCTGCGCAAGCTGCGTAAGCGGCTCCTCCGCGATGCCGATGACCTGCAGAAGCGCCTGGCAGTGTACCAGGCCGGGGCGAAT

>Seq141 [organism=Homo sapiens] Control ApoE ε3ε3

GACGTAGGGGGGCGCGGAATGGAGGACGTGTGCGGCCGCCTGGTGCAGTACCGCGGCGAGGTGCAGGCCATGCTCGGCCAGAGCACCGAGGAGCTGCGGGTGCGCCTCGCCTCCCACCTGCGCAAGCTGCGTAAGCGGCTCCTCCGCGATGCCGATGACCTGCAGAAGCGCCTGGCAGTGTACCAGGCCGGGGCGAAT

>Seq142 [organism=Homo sapiens] Control ApoE ε3ε3

GACGTAGTGGGGCGCGGAATGGAGGACGTGTGCGGCCGCCTGGTGCAGTACCGCGGCGAGGTGCAGGCCATGCTCGGCCAGAGCACCGAGGAGCTGCGGGTGCGCCTCGCCTCCCACCTGCGCAAGCTGCGTAAGCGGCTCCTCCGCGATGCCGATGACCTGCAGAAGCGCCTGGCAGTGTACCAGGCCGGGGCGAAT

>Seq143 [organism=Homo sapiens] Control ApoE ε3ε3

GACGTAGCGGGGCGCGGAATGGAGGACGTGTGCGGCCGCCTGGTGCAGTACCGCGGCGAGGTGCAGGCCATGCTCGGCCAGAGCACCGAGGAGCTGCGGGTGCGCCTCGCCTCCCACCTGCGCAAGCTGCGTAAGCGGCTCCTCCGCGATGCCGATGACCTGCAGAAGCGCCTGGCAGTGTACCAGGCCGGGGCGAAT

>Seq144 [organism=Homo sapiens] Control ApoE ε3ε3

GACGTAGTGGGGCGCGGAATGGAGGACGTGTGCGGCCGCCTGGTGCAGTACCGCGGCGAGGTGCAGGCCATGCTCGGCCAGAGCACCGAGGAGCTGCGGGTGCGCCTCGCCTCCCACCTGCGCAAGCTGCGTAAGCGGCTCCTCCGCGATGCCGATGACCTGCAGAAGCGCCTGGCAGTGTACCAGGCCGGGGCGAAT

>Seq145 [organism=Homo sapiens] Control ApoE ε3ε3

GACGTAGCGGGGCGCGGAATGGAGGACGTGTGCGGCCGCCTGGTGCAGTACCGCGGCGAGGTGCAGGCCATGCTCGGCCAGAGCACCGAGGAGCTGCGGGTGCGCCTCGCCTCCCACCTGCGCAAGCTGCGTAAGCGGCTCCTCCGCGATGCCGATGACCTGCAGAAGCGCCTGGCAGTGTACCAGGCCGGGGCGAAT

>Seq146 [organism=Homo sapiens] Control ApoE ε3ε3

GACGTAGGGGGGCGCGGAATGGAGGACGTGTGCGGCCGCCTGGTGCAGTACCGCGGCGAGGTGCAGGCCATGCTCGGCCAGAGCACCGAGGAGCTGCGGGTGCGCCTCGCCTCCCACCTGCGCAAGCTGCGTAAGCGGCTCCTCCGCGATGCCGATGACCTGCAGAAGCGCCTGGCAGTGTACCAGGCCGGGGCGAAT

>Seq147 [organism=Homo sapiens] Control ApoE ε3ε3

GACGTAGCGGGGCGCGGAATGGAGGACGTGTGCGGCCGCCTGGTGCAGTACCGCGGCGAGGTGCAGGCCATGCTCGGCCAGAGCACCGAGGAGCTGCGGGTGCGCCTCGCCTCCCACCTGCGCAAGCTGCGTAAGCGGCTCCTCCGCGATGCCGATGACCTGCAGAAGCGCCTGGCAGTGTACCAGGCCGGGGCGAAT

>Seq148 [organism=Homo sapiens] Control ApoE ε3ε3

GACGTAGTGGGGCGCGGAATGGAGGACGTGTGCGGCCGCCTGGTGCAGTACCGCGGCGAGGTGCAGGCCATGCTCGGCCAGAGCACCGAGGAGCTGCGGGTGCGCCTCGCCTCCCACCTGCGCAAGCTGCGTAAGCGGCTCCTCCGCGATGCCGATGACCTGCAGAAGCGCCTGGCAGTGTACCAGGCCGGGGCGAAT

>Seq149 [organism=Homo sapiens] Control ApoE ε3ε3

GACGTAGCGGGGCGCGGAATGGAGGACGTGTGCGGCCGCCTGGTGCAGTACCGCGGCGAGGTGCAGGCCATGCTCGGCCAGAGCACCGAGGAGCTGCGGGTGCGCCTCGCCTCCCACCTGCGCAAGCTGCGTAAGCGGCTCCTCCGCGATGCCGATGACCTGCAGAAGCGCCTGGCAGTGTACCAGGCCGGGGCGAAT

>Seq150 [organism=Homo sapiens] Control ApoE ε3ε3

GACGTAGGGGGGCGCGGAATGGAGGACGTGTGCGGCCGCCTGGTGCAGTACCGCGGCGAGGTGCAGGCCATGCTCGGCCAGAGCACCGAGGAGCTGCGGGTGCGCCTCGCCTCCCACCTGCGCAAGCTGCGTAAGCGGCTCCTCCGCGATGCCGATGACCTGCAGAAGCGCCTGGCAGTGTACCAGGCCGGGGCGAAT

>Seq151 [organism=Homo sapiens] Control ApoE ε3ε3

GACGTAGTGGGGCGCGGAATGGAGGACGTGTGCGGCCGCCTGGTGCAGTACCGCGGCGAGGTGCAGGCCATGCTCGGCCAGAGCACCGAGGAGCTGCGGGTGCGCCTCGCCTCCCACCTGCGCAAGCTGCGTAAGCGGCTCCTCCGCGATGCCGATGACCTGCAGAAGCGCCTGGCAGTGTACCAGGCCGGGGCGAAT

>Seq152 [organism=Homo sapiens] Control ApoE ε3ε3

GACGTAGCGGGGCGCGGAATGGAGGACGTGTGCGGCCGCCTGGTGCAGTACCGCGGCGAGGTGCAGGCCATGCTCGGCCAGAGCACCGAGGAGCTGCGGGTGCGCCTCGCCTCCCACCTGCGCAAGCTGCGTAAGCGGCTCCTCCGCGATGCCGATGACCTGCAGAAGCGCCTGGCAGTGTACCAGGCCGGGGCGAAT

>Seq153 [organism=Homo sapiens] Control ApoE ε3ε3

ATCGTAGCGGGGCGCGGAATGGAGGACGTGTGCGGCCGCCTGGTGCAGTACCGCGGCGAGGTGCAGGCCATGCTCGGCCAGAGCACCGAGGAGCTGCGGGTGCGCCTCGCCTCCCACCTGCGCAAGCTGCGTAAGCGGCTCCTCCGCGATGCCGATGACCTGCAGAAGCGCCTGGCAGTGTACCAGGCCGGGGCGAAT

>Seq154 [organism=Homo sapiens] Control ApoE ε3ε3

GACCTAGCGGGGCGCGGAATGGAGGACGTGTGCGGCCGCCTGGTGCAGTACCGCGGCGAGGTGCAGGCCATGCTCGGCCAGAGCACCGAGGAGCTGCGGGTGCGCCTCGCCTCCCACCTGCGCAAGCTGCGTAAGCGGCTCCTCCGCGATGCCGATGACCTGCAGAAGCGCCTGGCAGTGTACCAGGCCGGGGCGAAT

>Seq155 [organism=Homo sapiens] Control ApoE ε3ε3

GACGTTCGGGGGCGCGGAATGGAGGACGTGTGCGGCCGCCTGGTGCAGTACCGCGGCGAGGTGCAGGCCATGCTCGGCCAGAGCACCGAGGAGCTGCGGGTGCGCCTCGCCTCCCACCTGCGCAAGCTGCGTAAGCGGCTCCTCCGCGATGCCGATGACCTGCAGAAGCGCCTGGCAGTGTACCAGGCCGGGGCGAAT

>Seq156 [organism=Homo sapiens] Control ApoE ε3ε3

GACGTAGTGGGGCGCGGAATGGAGGACGTGTGCGGCCGCCTGGTGCAGTACCGCGGCGAGGTGCAGGCCATGCTCGGCCAGAGCACCGAGGAGCTGCGGGTGCGCCTCGCCTCCCACCTGCGCAAGCTGCGTAAGCGGCTCCTCCGCGATGCCGATGACCTGCAGAAGCGCCTGGCAGTGTACCAGGCCGGGGCGAAT

>Seq157 [organism=Homo sapiens] Control ApoE ε3ε3

GACGTAGCGGGGCGCGGAATGGAGGACGTGTGCGGCCGCCTGGTGCAGTACCGCGGCGAGGTGCAGGCCATGCTCGGCCAGAGCACCGAGGAGCTGCGGGTGCGCCTCGCCTCCCACCTGCGCAAGCTGCGTAAGCGGCTCCTCCGCGATGCCGATGACCTGCAGAAGCGCCTGGCAGTGTACCAGGCCGGGGCGAAT

>Seq158 [organism=Homo sapiens] Control ApoE ε3ε3

GTCGTAGCGGGGCGCGGAATGGAGGACGTGTGCGGCCGCCTGGTGCAGTACCGCGGCGAGGTGCAGGCCATGCTCGGCCAGAGCACCGAGGAGCTGCGGGTGCGCCTCGCCTCCCACCTGCGCAAGCTGCGTAAGCGGCTCCTCCGCGATGCCGATGACCTGCAGAAGCGCCTGGCAGTGTACCAGGCCGGGGCGAAT

>Seq159 [organism=Homo sapiens] Control ApoE ε3ε3

GACATAGCGGGGCGCGGAATGGAGGACGTGTGCGGCCGCCTGGTGCAGTACCGCGGCGAGGTGCAGGCCATGCTCGGCCAGAGCACCGAGGAGCTGCGGGTGCGCCTCGCCTCCCACCTGCGCAAGCTGCGTAAGCGGCTCCTCCGCGATGCCGATGACCTGCAGAAGCGCCTGGCAGTGTACCAGGCCGGGGCGAAT

>Seq160 [organism=Homo sapiens] Control ApoE ε3ε3

GACGGAGCGGGGCGCGGAATGGAGGACGTGTGCGGCCGCCTGGTGCAGTACCGCGGCGAGGTGCAGGCCATGCTCGGCCAGAGCACCGAGGAGCTGCGGGTGCGCCTCGCCTCCCACCTGCGCAAGCTGCGTAAGCGGCTCCTCCGCGATGCCGATGACCTGCAGAAGCGCCTGGCAGTGTACCAGGCCGGGGCGAAT

>Seq161 [organism=Homo sapiens] Control ApoE ε3ε3

GACGTAGGGGGGCGCGGAATGGAGGACGTGTGCGGCCGCCTGGTGCAGTACCGCGGCGAGGTGCAGGCCATGCTCGGCCAGAGCACCGAGGAGCTGCGGGTGCGCCTCGCCTCCCACCTGCGCAAGCTGCGTAAGCGGCTCCTCCGCGATGCCGATGACCTGCAGAAGCGCCTGGCAGTGTACCAGGCCGGGGCGAAT

>Seq162 [organism=Homo sapiens] Control ApoE ε3ε3

GACGTAGTGGGGCGCGGAATGGAGGACGTGTGCGGCCGCCTGGTGCAGTACCGCGGCGAGGTGCAGGCCATGCTCGGCCAGAGCACCGAGGAGCTGCGGGTGCGCCTCGCCTCCCACCTGCGCAAGCTGCGTAAGCGGCTCCTCCGCGATGCCGATGACCTGCAGAAGCGCCTGGCAGTGTACCAGGCCGGGGCGAAT

>Seq163 [organism=Homo sapiens] Control ApoE ε3ε3

GACGTAGCGGGGCGCGGAATGGAGGACGTGTGCGGCCGCCTGGTGCAGTACCGCGGCGAGGTGCAGGCCATGCTCGGCCAGAGCACCGAGGAGCTGCGGGTGCGCCTCGCCTCCCACCTGCGCAAGCTGCGTAAGCGGCTCCTCCGCGATGCCGATGACCTGCAGAAGCGCCTGGCAGTGTACCAGGCCGGGGCGAAT

>Seq164 [organism=Homo sapiens] Control ApoE ε3ε3

GACGTAGTGGGGCGCGGAATGGAGGACGTGTGCGGCCGCCTGGTGCAGTACCGCGGCGAGGTGCAGGCCATGCTCGGCCAGAGCACCGAGGAGCTGCGGGTGCGCCTCGCCTCCCACCTGCGCAAGCTGCGTAAGCGGCTCCTCCGCGATGCCGATGACCTGCAGAAGCGCCTGGCAGTGTACCAGGCCGGGGCGAAT

>Seq165 [organism=Homo sapiens] Control ApoE ε3ε3

GACGTAGCGGGGCGCGGAATGGAGGACGTGTGCGGCCGCCTGGTGCAGTACCGCGGCGAGGTGCAGGCCATGCTCGGCCAGAGCACCGAGGAGCTGCGGGTGCGCCTCGCCTCCCACCTGCGCAAGCTGCGTAAGCGGCTCCTCCGCGATGCCGATGACCTGCAGAAGCGCCTGGCAGTGTACCAGGCCGGGGCGAAT

>Seq166 [organism=Homo sapiens] Control ApoE ε3ε3

GACGTAGGGGGGCGCGGAATGGAGGACGTGTGCGGCCGCCTGGTGCAGTACCGCGGCGAGGTGCAGGCCATGCTCGGCCAGAGCACCGAGGAGCTGCGGGTGCGCCTCGCCTCCCACCTGCGCAAGCTGCGTAAGCGGCTCCTCCGCGATGCCGATGACCTGCAGAAGCGCCTGGCAGTGTACCAGGCCGGGGCGAAT

>Seq167 [organism=Homo sapiens] Control ApoE ε3ε3

GACGTAGCGGGGCGCGGAATGGAGGACGTGTGCGGCCGCCTGGTGCAGTACCGCGGCGAGGTGCAGGCCATGCTCGGCCAGAGCACCGAGGAGCTGCGGGTGCGCCTCGCCTCCCACCTGCGCAAGCTGCGTAAGCGGCTCCTCCGCGATGCCGATGACCTGCAGAAGCGCCTGGCAGTGTACCAGGCCGGGGCGAAT

>Seq168 [organism=Homo sapiens] Control ApoE ε3ε3

GACGTAGTGGGGCGCGGAATGGAGGACGTGTGCGGCCGCCTGGTGCAGTACCGCGGCGAGGTGCAGGCCATGCTCGGCCAGAGCACCGAGGAGCTGCGGGTGCGCCTCGCCTCCCACCTGCGCAAGCTGCGTAAGCGGCTCCTCCGCGATGCCGATGACCTGCAGAAGCGCCTGGCAGTGTACCAGGCCGGGGCGAAT

>Seq169 [organism=Homo sapiens] Control ApoE ε3ε3

GACGTAGCGGGGCGCGGAATGGAGGACGTGTGCGGCCGCCTGGTGCAGTACCGCGGCGAGGTGCAGGCCATGCTCGGCCAGAGCACCGAGGAGCTGCGGGTGCGCCTCGCCTCCCACCTGCGCAAGCTGCGTAAGCGGCTCCTCCGCGATGCCGATGACCTGCAGAAGCGCCTGGCAGTGTACCAGGCCGGGGCGAAT

>Seq170 [organism=Homo sapiens] Control ApoE ε3ε3

GACGTAGGGGGGCGCGGAATGGAGGACGTGTGCGGCCGCCTGGTGCAGTACCGCGGCGAGGTGCAGGCCATGCTCGGCCAGAGCACCGAGGAGCTGCGGGTGCGCCTCGCCTCCCACCTGCGCAAGCTGCGTAAGCGGCTCCTCCGCGATGCCGATGACCTGCAGAAGCGCCTGGCAGTGTACCAGGCCGGGGCGAAT

>Seq171 [organism=Homo sapiens] Control ApoE ε3ε3

GACGTAGTGGGGCGCGGAATGGAGGACGTGTGCGGCCGCCTGGTGCAGTACCGCGGCGAGGTGCAGGCCATGCTCGGCCAGAGCACCGAGGAGCTGCGGGTGCGCCTCGCCTCCCACCTGCGCAAGCTGCGTAAGCGGCTCCTCCGCGATGCCGATGACCTGCAGAAGCGCCTGGCAGTGTACCAGGCCGGGGCGAAT

>Seq172 [organism=Homo sapiens] Control ApoE ε3ε3

GACGTAGCGGGGCGCGGAATGGAGGACGTGTGCGGCCGCCTGGTGCAGTACCGCGGCGAGGTGCAGGCCATGCTCGGCCAGAGCACCGAGGAGCTGCGGGTGCGCCTCGCCTCCCACCTGCGCAAGCTGCGTAAGCGGCTCCTCCGCGATGCCGATGACCTGCAGAAGCGCCTGGCAGTGTACCAGGCCGGGGCGAAT

>Seq173 [organism=Homo sapiens] Control ApoE ε3ε3

ATCGTAGCGGGGCGCGGAATGGAGGACGTGTGCGGCCGCCTGGTGCAGTACCGCGGCGAGGTGCAGGCCATGCTCGGCCAGAGCACCGAGGAGCTGCGGGTGCGCCTCGCCTCCCACCTGCGCAAGCTGCGTAAGCGGCTCCTCCGCGATGCCGATGACCTGCAGAAGCGCCTGGCAGTGTACCAGGCCGGGGCGAAT

>Seq174 [organism=Homo sapiens] Control ApoE ε3ε3

GACCTAGCGGGGCGCGGAATGGAGGACGTGTGCGGCCGCCTGGTGCAGTACCGCGGCGAGGTGCAGGCCATGCTCGGCCAGAGCACCGAGGAGCTGCGGGTGCGCCTCGCCTCCCACCTGCGCAAGCTGCGTAAGCGGCTCCTCCGCGATGCCGATGACCTGCAGAAGCGCCTGGCAGTGTACCAGGCCGGGGCGAAT

>Seq175 [organism=Homo sapiens] Control ApoE ε3ε3

GACGTTCGGGGGCGCGGAATGGAGGACGTGTGCGGCCGCCTGGTGCAGTACCGCGGCGAGGTGCAGGCCATGCTCGGCCAGAGCACCGAGGAGCTGCGGGTGCGCCTCGCCTCCCACCTGCGCAAGCTGCGTAAGCGGCTCCTCCGCGATGCCGATGACCTGCAGAAGCGCCTGGCAGTGTACCAGGCCGGGGCGAAT

>Seq176 [organism=Homo sapiens] Control ApoE ε3ε3

GACGTAGTGGGGCGCGGAATGGAGGACGTGTGCGGCCGCCTGGTGCAGTACCGCGGCGAGGTGCAGGCCATGCTCGGCCAGAGCACCGAGGAGCTGCGGGTGCGCCTCGCCTCCCACCTGCGCAAGCTGCGTAAGCGGCTCCTCCGCGATGCCGATGACCTGCAGAAGCGCCTGGCAGTGTACCAGGCCGGGGCGAAT

>Seq177 [organism=Homo sapiens] Control ApoE ε3ε3

GACGTAGCGGGGCGCGGAATGGAGGACGTGTGCGGCCGCCTGGTGCAGTACCGCGGCGAGGTGCAGGCCATGCTCGGCCAGAGCACCGAGGAGCTGCGGGTGCGCCTCGCCTCCCACCTGCGCAAGCTGCGTAAGCGGCTCCTCCGCGATGCCGATGACCTGCAGAAGCGCCTGGCAGTGTACCAGGCCGGGGCGAAT

>Seq178 [organism=Homo sapiens] Control ApoE ε3ε3

GTCGTAGCGGGGCGCGGAATGGAGGACGTGTGCGGCCGCCTGGTGCAGTACCGCGGCGAGGTGCAGGCCATGCTCGGCCAGAGCACCGAGGAGCTGCGGGTGCGCCTCGCCTCCCACCTGCGCAAGCTGCGTAAGCGGCTCCTCCGCGATGCCGATGACCTGCAGAAGCGCCTGGCAGTGTACCAGGCCGGGGCGAAT

>Seq179 [organism=Homo sapiens] Control ApoE ε3ε3

GACATAGCGGGGCGCGGAATGGAGGACGTGTGCGGCCGCCTGGTGCAGTACCGCGGCGAGGTGCAGGCCATGCTCGGCCAGAGCACCGAGGAGCTGCGGGTGCGCCTCGCCTCCCACCTGCGCAAGCTGCGTAAGCGGCTCCTCCGCGATGCCGATGACCTGCAGAAGCGCCTGGCAGTGTACCAGGCCGGGGCGAAT

>Seq180 [organism=Homo sapiens] Control ApoE ε3ε3

GACGGAGCGGGGCGCGGAATGGAGGACGTGTGCGGCCGCCTGGTGCAGTACCGCGGCGAGGTGCAGGCCATGCTCGGCCAGAGCACCGAGGAGCTGCGGGTGCGCCTCGCCTCCCACCTGCGCAAGCTGCGTAAGCGGCTCCTCCGCGATGCCGATGACCTGCAGAAGCGCCTGGCAGTGTACCAGGCCGGGGCGAAT

>Seq181 [organism=Homo sapiens] Control ApoE ε3ε3

GACGTAGGGGGGCGCGGAATGGAGGACGTGTGCGGCCGCCTGGTGCAGTACCGCGGCGAGGTGCAGGCCATGCTCGGCCAGAGCACCGAGGAGCTGCGGGTGCGCCTCGCCTCCCACCTGCGCAAGCTGCGTAAGCGGCTCCTCCGCGATGCCGATGACCTGCAGAAGCGCCTGGCAGTGTACCAGGCCGGGGCGAAT

>Seq182 [organism=Homo sapiens] Control ApoE ε3ε3

GACGTAGTGGGGCGCGGAATGGAGGACGTGTGCGGCCGCCTGGTGCAGTACCGCGGCGAGGTGCAGGCCATGCTCGGCCAGAGCACCGAGGAGCTGCGGGTGCGCCTCGCCTCCCACCTGCGCAAGCTGCGTAAGCGGCTCCTCCGCGATGCCGATGACCTGCAGAAGCGCCTGGCAGTGTACCAGGCCGGGGCGAAT

>Seq183 [organism=Homo sapiens] Control ApoE ε3ε3

GACGTAGCGGGGCGCGGAATGGAGGACGTGTGCGGCCGCCTGGTGCAGTACCGCGGCGAGGTGCAGGCCATGCTCGGCCAGAGCACCGAGGAGCTGCGGGTGCGCCTCGCCTCCCACCTGCGCAAGCTGCGTAAGCGGCTCCTCCGCGATGCCGATGACCTGCAGAAGCGCCTGGCAGTGTACCAGGCCGGGGCGAAT

>Seq184 [organism=Homo sapiens] Control ApoE ε3ε3

GACGTAGTGGGGCGCGGAATGGAGGACGTGTGCGGCCGCCTGGTGCAGTACCGCGGCGAGGTGCAGGCCATGCTCGGCCAGAGCACCGAGGAGCTGCGGGTGCGCCTCGCCTCCCACCTGCGCAAGCTGCGTAAGCGGCTCCTCCGCGATGCCGATGACCTGCAGAAGCGCCTGGCAGTGTACCAGGCCGGGGCGAAT

>Seq185 [organism=Homo sapiens] Control ApoE ε3ε3

GACGTAGCGGGGCGCGGAATGGAGGACGTGTGCGGCCGCCTGGTGCAGTACCGCGGCGAGGTGCAGGCCATGCTCGGCCAGAGCACCGAGGAGCTGCGGGTGCGCCTCGCCTCCCACCTGCGCAAGCTGCGTAAGCGGCTCCTCCGCGATGCCGATGACCTGCAGAAGCGCCTGGCAGTGTACCAGGCCGGGGCGAAT

>Seq186 [organism=Homo sapiens] Control ApoE ε3ε3

GACGTAGGGGGGCGCGGAATGGAGGACGTGTGCGGCCGCCTGGTGCAGTACCGCGGCGAGGTGCAGGCCATGCTCGGCCAGAGCACCGAGGAGCTGCGGGTGCGCCTCGCCTCCCACCTGCGCAAGCTGCGTAAGCGGCTCCTCCGCGATGCCGATGACCTGCAGAAGCGCCTGGCAGTGTACCAGGCCGGGGCGAAT

>Seq187 [organism=Homo sapiens] Control ApoE ε3ε3

GACGTAGCGGGGCGCGGAATGGAGGACGTGTGCGGCCGCCTGGTGCAGTACCGCGGCGAGGTGCAGGCCATGCTCGGCCAGAGCACCGAGGAGCTGCGGGTGCGCCTCGCCTCCCACCTGCGCAAGCTGCGTAAGCGGCTCCTCCGCGATGCCGATGACCTGCAGAAGCGCCTGGCAGTGTACCAGGCCGGGGCGAAT

>Seq188 [organism=Homo sapiens] Control ApoE ε3ε3

GACGTAGTGGGGCGCGGAATGGAGGACGTGTGCGGCCGCCTGGTGCAGTACCGCGGCGAGGTGCAGGCCATGCTCGGCCAGAGCACCGAGGAGCTGCGGGTGCGCCTCGCCTCCCACCTGCGCAAGCTGCGTAAGCGGCTCCTCCGCGATGCCGATGACCTGCAGAAGCGCCTGGCAGTGTACCAGGCCGGGGCGAAT

>Seq189 [organism=Homo sapiens] Control ApoE ε3ε3

GACGTAGCGGGGCGCGGAATGGAGGACGTGTGCGGCCGCCTGGTGCAGTACCGCGGCGAGGTGCAGGCCATGCTCGGCCAGAGCACCGAGGAGCTGCGGGTGCGCCTCGCCTCCCACCTGCGCAAGCTGCGTAAGCGGCTCCTCCGCGATGCCGATGACCTGCAGAAGCGCCTGGCAGTGTACCAGGCCGGGGCGAAT

>Seq190 [organism=Homo sapiens] Control ApoE ε3ε3

GACGTAGGGGGGCGCGGAATGGAGGACGTGTGCGGCCGCCTGGTGCAGTACCGCGGCGAGGTGCAGGCCATGCTCGGCCAGAGCACCGAGGAGCTGCGGGTGCGCCTCGCCTCCCACCTGCGCAAGCTGCGTAAGCGGCTCCTCCGCGATGCCGATGACCTGCAGAAGCGCCTGGCAGTGTACCAGGCCGGGGCGAAT

>Seq191 [organism=Homo sapiens] Control ApoE ε3ε3

GACGTAGTGGGGCGCGGAATGGAGGACGTGTGCGGCCGCCTGGTGCAGTACCGCGGCGAGGTGCAGGCCATGCTCGGCCAGAGCACCGAGGAGCTGCGGGTGCGCCTCGCCTCCCACCTGCGCAAGCTGCGTAAGCGGCTCCTCCGCGATGCCGATGACCTGCAGAAGCGCCTGGCAGTGTACCAGGCCGGGGCGAAT

>Seq192 [organism=Homo sapiens] Control ApoE ε3ε3

GACGTAGCGGGGCGCGGAATGGAGGACGTGTGCGGCCGCCTGGTGCAGTACCGCGGCGAGGTGCAGGCCATGCTCGGCCAGAGCACCGAGGAGCTGCGGGTGCGCCTCGCCTCCCACCTGCGCAAGCTGCGTAAGCGGCTCCTCCGCGATGCCGATGACCTGCAGAAGCGCCTGGCAGTGTACCAGGCCGGGGCGAAT

>Seq193 [organism=Homo sapiens] Control ApoE ε3ε3

ATCGTAGCGGGGCGCGGAATGGAGGACGTGTGCGGCCGCCTGGTGCAGTACCGCGGCGAGGTGCAGGCCATGCTCGGCCAGAGCACCGAGGAGCTGCGGGTGCGCCTCGCCTCCCACCTGCGCAAGCTGCGTAAGCGGCTCCTCCGCGATGCCGATGACCTGCAGAAGCGCCTGGCAGTGTACCAGGCCGGGGCGAAT

>Seq194 [organism=Homo sapiens] Control ApoE ε3ε3

GACCTAGCGGGGCGCGGAATGGAGGACGTGTGCGGCCGCCTGGTGCAGTACCGCGGCGAGGTGCAGGCCATGCTCGGCCAGAGCACCGAGGAGCTGCGGGTGCGCCTCGCCTCCCACCTGCGCAAGCTGCGTAAGCGGCTCCTCCGCGATGCCGATGACCTGCAGAAGCGCCTGGCAGTGTACCAGGCCGGGGCGAAT

>Seq195 [organism=Homo sapiens] Control ApoE ε3ε3

GACGTTCGGGGGCGCGGAATGGAGGACGTGTGCGGCCGCCTGGTGCAGTACCGCGGCGAGGTGCAGGCCATGCTCGGCCAGAGCACCGAGGAGCTGCGGGTGCGCCTCGCCTCCCACCTGCGCAAGCTGCGTAAGCGGCTCCTCCGCGATGCCGATGACCTGCAGAAGCGCCTGGCAGTGTACCAGGCCGGGGCGAAT

>Seq196 [organism=Homo sapiens] Control ApoE ε3ε3

GACGTAGTGGGGCGCGGAATGGAGGACGTGTGCGGCCGCCTGGTGCAGTACCGCGGCGAGGTGCAGGCCATGCTCGGCCAGAGCACCGAGGAGCTGCGGGTGCGCCTCGCCTCCCACCTGCGCAAGCTGCGTAAGCGGCTCCTCCGCGATGCCGATGACCTGCAGAAGCGCCTGGCAGTGTACCAGGCCGGGGCGAAT

>Seq197 [organism=Homo sapiens] Control ApoE ε3ε3

GACGTAGCGGGGCGCGGAATGGAGGACGTGTGCGGCCGCCTGGTGCAGTACCGCGGCGAGGTGCAGGCCATGCTCGGCCAGAGCACCGAGGAGCTGCGGGTGCGCCTCGCCTCCCACCTGCGCAAGCTGCGTAAGCGGCTCCTCCGCGATGCCGATGACCTGCAGAAGCGCCTGGCAGTGTACCAGGCCGGGGCGAAT

>Seq198 [organism=Homo sapiens] Control ApoE ε3ε3

GTCGTAGCGGGGCGCGGAATGGAGGACGTGTGCGGCCGCCTGGTGCAGTACCGCGGCGAGGTGCAGGCCATGCTCGGCCAGAGCACCGAGGAGCTGCGGGTGCGCCTCGCCTCCCACCTGCGCAAGCTGCGTAAGCGGCTCCTCCGCGATGCCGATGACCTGCAGAAGCGCCTGGCAGTGTACCAGGCCGGGGCGAAT

>Seq199 [organism=Homo sapiens] Control ApoE ε3ε3

GACATAGCGGGGCGCGGAATGGAGGACGTGTGCGGCCGCCTGGTGCAGTACCGCGGCGAGGTGCAGGCCATGCTCGGCCAGAGCACCGAGGAGCTGCGGGTGCGCCTCGCCTCCCACCTGCGCAAGCTGCGTAAGCGGCTCCTCCGCGATGCCGATGACCTGCAGAAGCGCCTGGCAGTGTACCAGGCCGGGGCGAAT

>Seq200 [organism=Homo sapiens] Control ApoE ε3ε3

GACGGAGCGGGGCGCGGAATGGAGGACGTGTGCGGCCGCCTGGTGCAGTACCGCGGCGAGGTGCAGGCCATGCTCGGCCAGAGCACCGAGGAGCTGCGGGTGCGCCTCGCCTCCCACCTGCGCAAGCTGCGTAAGCGGCTCCTCCGCGATGCCGATGACCTGCAGAAGCGCCTGGCAGTGTACCAGGCCGGGGCGAAT

>Seq201 [organism=Homo sapiens] Control ApoE ε3ε3

GACGTAGGGGGGCGCGGAATGGAGGACGTGTGCGGCCGCCTGGTGCAGTACCGCGGCGAGGTGCAGGCCATGCTCGGCCAGAGCACCGAGGAGCTGCGGGTGCGCCTCGCCTCCCACCTGCGCAAGCTGCGTAAGCGGCTCCTCCGCGATGCCGATGACCTGCAGAAGCGCCTGGCAGTGTACCAGGCCGGGGCGAAT

>Seq202 [organism=Homo sapiens] Control ApoE ε3ε3

GACGTAGTGGGGCGCGGAATGGAGGACGTGTGCGGCCGCCTGGTGCAGTACCGCGGCGAGGTGCAGGCCATGCTCGGCCAGAGCACCGAGGAGCTGCGGGTGCGCCTCGCCTCCCACCTGCGCAAGCTGCGTAAGCGGCTCCTCCGCGATGCCGATGACCTGCAGAAGCGCCTGGCAGTGTACCAGGCCGGGGCGAAT

>Seq203 [organism=Homo sapiens] Control ApoE ε3ε3

GACGTAGCGGGGCGCGGAATGGAGGACGTGTGCGGCCGCCTGGTGCAGTACCGCGGCGAGGTGCAGGCCATGCTCGGCCAGAGCACCGAGGAGCTGCGGGTGCGCCTCGCCTCCCACCTGCGCAAGCTGCGTAAGCGGCTCCTCCGCGATGCCGATGACCTGCAGAAGCGCCTGGCAGTGTACCAGGCCGGGGCGAAT

>Seq204 [organism=Homo sapiens] Control ApoE ε3ε3

GACGTAGTGGGGCGCGGAATGGAGGACGTGTGCGGCCGCCTGGTGCAGTACCGCGGCGAGGTGCAGGCCATGCTCGGCCAGAGCACCGAGGAGCTGCGGGTGCGCCTCGCCTCCCACCTGCGCAAGCTGCGTAAGCGGCTCCTCCGCGATGCCGATGACCTGCAGAAGCGCCTGGCAGTGTACCAGGCCGGGGCGAAT

>Seq205 [organism=Homo sapiens] Control ApoE ε3ε3

GACGTAGCGGGGCGCGGAATGGAGGACGTGTGCGGCCGCCTGGTGCAGTACCGCGGCGAGGTGCAGGCCATGCTCGGCCAGAGCACCGAGGAGCTGCGGGTGCGCCTCGCCTCCCACCTGCGCAAGCTGCGTAAGCGGCTCCTCCGCGATGCCGATGACCTGCAGAAGCGCCTGGCAGTGTACCAGGCCGGGGCGAAT

>Seq206 [organism=Homo sapiens] Control ApoE ε3ε3

GACGTAGGGGGGCGCGGAATGGAGGACGTGTGCGGCCGCCTGGTGCAGTACCGCGGCGAGGTGCAGGCCATGCTCGGCCAGAGCACCGAGGAGCTGCGGGTGCGCCTCGCCTCCCACCTGCGCAAGCTGCGTAAGCGGCTCCTCCGCGATGCCGATGACCTGCAGAAGCGCCTGGCAGTGTACCAGGCCGGGGCGAAT

>Seq207 [organism=Homo sapiens] Control ApoE ε3ε3

GACGTAGCGGGGCGCGGAATGGAGGACGTGTGCGGCCGCCTGGTGCAGTACCGCGGCGAGGTGCAGGCCATGCTCGGCCAGAGCACCGAGGAGCTGCGGGTGCGCCTCGCCTCCCACCTGCGCAAGCTGCGTAAGCGGCTCCTCCGCGATGCCGATGACCTGCAGAAGCGCCTGGCAGTGTACCAGGCCGGGGCGAAT

>Seq208 [organism=Homo sapiens] Control ApoE ε3ε3

GACGTAGTGGGGCGCGGAATGGAGGACGTGTGCGGCCGCCTGGTGCAGTACCGCGGCGAGGTGCAGGCCATGCTCGGCCAGAGCACCGAGGAGCTGCGGGTGCGCCTCGCCTCCCACCTGCGCAAGCTGCGTAAGCGGCTCCTCCGCGATGCCGATGACCTGCAGAAGCGCCTGGCAGTGTACCAGGCCGGGGCGAAT

>Seq209 [organism=Homo sapiens] Control ApoE ε3ε3

GACGTAGCGGGGCGCGGAATGGAGGACGTGTGCGGCCGCCTGGTGCAGTACCGCGGCGAGGTGCAGGCCATGCTCGGCCAGAGCACCGAGGAGCTGCGGGTGCGCCTCGCCTCCCACCTGCGCAAGCTGCGTAAGCGGCTCCTCCGCGATGCCGATGACCTGCAGAAGCGCCTGGCAGTGTACCAGGCCGGGGCGAAT

>Seq210 [organism=Homo sapiens] Control ApoE ε3ε3

GACGTAGGGGGGCGCGGAATGGAGGACGTGTGCGGCCGCCTGGTGCAGTACCGCGGCGAGGTGCAGGCCATGCTCGGCCAGAGCACCGAGGAGCTGCGGGTGCGCCTCGCCTCCCACCTGCGCAAGCTGCGTAAGCGGCTCCTCCGCGATGCCGATGACCTGCAGAAGCGCCTGGCAGTGTACCAGGCCGGGGCGAAT

>Seq211 [organism=Homo sapiens] Control ApoE ε3ε3

GACGTAGTGGGGCGCGGAATGGAGGACGTGTGCGGCCGCCTGGTGCAGTACCGCGGCGAGGTGCAGGCCATGCTCGGCCAGAGCACCGAGGAGCTGCGGGTGCGCCTCGCCTCCCACCTGCGCAAGCTGCGTAAGCGGCTCCTCCGCGATGCCGATGACCTGCAGAAGCGCCTGGCAGTGTACCAGGCCGGGGCGAAT

>Seq212 [organism=Homo sapiens] Control ApoE ε3ε3

GACGTAGCGGGGCGCGGAATGGAGGACGTGTGCGGCCGCCTGGTGCAGTACCGCGGCGAGGTGCAGGCCATGCTCGGCCAGAGCACCGAGGAGCTGCGGGTGCGCCTCGCCTCCCACCTGCGCAAGCTGCGTAAGCGGCTCCTCCGCGATGCCGATGACCTGCAGAAGCGCCTGGCAGTGTACCAGGCCGGGGCGAAT

>Seq213 [organism=Homo sapiens] Control ApoE ε3ε3

ATCGTAGCGGGGCGCGGAATGGAGGACGTGTGCGGCCGCCTGGTGCAGTACCGCGGCGAGGTGCAGGCCATGCTCGGCCAGAGCACCGAGGAGCTGCGGGTGCGCCTCGCCTCCCACCTGCGCAAGCTGCGTAAGCGGCTCCTCCGCGATGCCGATGACCTGCAGAAGCGCCTGGCAGTGTACCAGGCCGGGGCGAAT

>Seq214 [organism=Homo sapiens] Control ApoE ε3ε3

GACCTAGCGGGGCGCGGAATGGAGGACGTGTGCGGCCGCCTGGTGCAGTACCGCGGCGAGGTGCAGGCCATGCTCGGCCAGAGCACCGAGGAGCTGCGGGTGCGCCTCGCCTCCCACCTGCGCAAGCTGCGTAAGCGGCTCCTCCGCGATGCCGATGACCTGCAGAAGCGCCTGGCAGTGTACCAGGCCGGGGCGAAT

>Seq215 [organism=Homo sapiens] Control ApoE ε3ε3

GACGTTCGGGGGCGCGGAATGGAGGACGTGTGCGGCCGCCTGGTGCAGTACCGCGGCGAGGTGCAGGCCATGCTCGGCCAGAGCACCGAGGAGCTGCGGGTGCGCCTCGCCTCCCACCTGCGCAAGCTGCGTAAGCGGCTCCTCCGCGATGCCGATGACCTGCAGAAGCGCCTGGCAGTGTACCAGGCCGGGGCGAAT

>Seq216 [organism=Homo sapiens] Control ApoE ε3ε3

GACGTAGTGGGGCGCGGAATGGAGGACGTGTGCGGCCGCCTGGTGCAGTACCGCGGCGAGGTGCAGGCCATGCTCGGCCAGAGCACCGAGGAGCTGCGGGTGCGCCTCGCCTCCCACCTGCGCAAGCTGCGTAAGCGGCTCCTCCGCGATGCCGATGACCTGCAGAAGCGCCTGGCAGTGTACCAGGCCGGGGCGAAT

>Seq217 [organism=Homo sapiens] Control ApoE ε3ε3

GACGTAGCGGGGCGCGGAATGGAGGACGTGTGCGGCCGCCTGGTGCAGTACCGCGGCGAGGTGCAGGCCATGCTCGGCCAGAGCACCGAGGAGCTGCGGGTGCGCCTCGCCTCCCACCTGCGCAAGCTGCGTAAGCGGCTCCTCCGCGATGCCGATGACCTGCAGAAGCGCCTGGCAGTGTACCAGGCCGGGGCGAAT

>Seq218 [organism=Homo sapiens] Control ApoE ε3ε3

GTCGTAGCGGGGCGCGGAATGGAGGACGTGTGCGGCCGCCTGGTGCAGTACCGCGGCGAGGTGCAGGCCATGCTCGGCCAGAGCACCGAGGAGCTGCGGGTGCGCCTCGCCTCCCACCTGCGCAAGCTGCGTAAGCGGCTCCTCCGCGATGCCGATGACCTGCAGAAGCGCCTGGCAGTGTACCAGGCCGGGGCGAAT

>Seq219 [organism=Homo sapiens] Control ApoE ε3ε3

GACATAGCGGGGCGCGGAATGGAGGACGTGTGCGGCCGCCTGGTGCAGTACCGCGGCGAGGTGCAGGCCATGCTCGGCCAGAGCACCGAGGAGCTGCGGGTGCGCCTCGCCTCCCACCTGCGCAAGCTGCGTAAGCGGCTCCTCCGCGATGCCGATGACCTGCAGAAGCGCCTGGCAGTGTACCAGGCCGGGGCGAAT

>Seq220 [organism=Homo sapiens] Control ApoE ε3ε3

GACGGAGCGGGGCGCGGAATGGAGGACGTGTGCGGCCGCCTGGTGCAGTACCGCGGCGAGGTGCAGGCCATGCTCGGCCAGAGCACCGAGGAGCTGCGGGTGCGCCTCGCCTCCCACCTGCGCAAGCTGCGTAAGCGGCTCCTCCGCGATGCCGATGACCTGCAGAAGCGCCTGGCAGTGTACCAGGCCGGGGCGAAT

>Seq221 [organism=Homo sapiens] Control ApoE ε3ε3

GACGTAGGGGGGCGCGGAATGGAGGACGTGTGCGGCCGCCTGGTGCAGTACCGCGGCGAGGTGCAGGCCATGCTCGGCCAGAGCACCGAGGAGCTGCGGGTGCGCCTCGCCTCCCACCTGCGCAAGCTGCGTAAGCGGCTCCTCCGCGATGCCGATGACCTGCAGAAGCGCCTGGCAGTGTACCAGGCCGGGGCGAAT

>Seq222 [organism=Homo sapiens] Control ApoE ε3ε3

GACGTAGTGGGGCGCGGAATGGAGGACGTGTGCGGCCGCCTGGTGCAGTACCGCGGCGAGGTGCAGGCCATGCTCGGCCAGAGCACCGAGGAGCTGCGGGTGCGCCTCGCCTCCCACCTGCGCAAGCTGCGTAAGCGGCTCCTCCGCGATGCCGATGACCTGCAGAAGCGCCTGGCAGTGTACCAGGCCGGGGCGAAT

>Seq223 [organism=Homo sapiens] Control ApoE ε3ε3

GACGTAGCGGGGCGCGGAATGGAGGACGTGTGCGGCCGCCTGGTGCAGTACCGCGGCGAGGTGCAGGCCATGCTCGGCCAGAGCACCGAGGAGCTGCGGGTGCGCCTCGCCTCCCACCTGCGCAAGCTGCGTAAGCGGCTCCTCCGCGATGCCGATGACCTGCAGAAGCGCCTGGCAGTGTACCAGGCCGGGGCGAAT

>Seq224 [organism=Homo sapiens] Control ApoE ε3ε3

GACGTAGTGGGGCGCGGAATGGAGGACGTGTGCGGCCGCCTGGTGCAGTACCGCGGCGAGGTGCAGGCCATGCTCGGCCAGAGCACCGAGGAGCTGCGGGTGCGCCTCGCCTCCCACCTGCGCAAGCTGCGTAAGCGGCTCCTCCGCGATGCCGATGACCTGCAGAAGCGCCTGGCAGTGTACCAGGCCGGGGCGAAT

>Seq225 [organism=Homo sapiens] Control ApoE ε3ε3

GACGTAGCGGGGCGCGGAATGGAGGACGTGTGCGGCCGCCTGGTGCAGTACCGCGGCGAGGTGCAGGCCATGCTCGGCCAGAGCACCGAGGAGCTGCGGGTGCGCCTCGCCTCCCACCTGCGCAAGCTGCGTAAGCGGCTCCTCCGCGATGCCGATGACCTGCAGAAGCGCCTGGCAGTGTACCAGGCCGGGGCGAAT

>Seq226 [organism=Homo sapiens] Control ApoE ε3ε3

GACGTAGGGGGGCGCGGAATGGAGGACGTGTGCGGCCGCCTGGTGCAGTACCGCGGCGAGGTGCAGGCCATGCTCGGCCAGAGCACCGAGGAGCTGCGGGTGCGCCTCGCCTCCCACCTGCGCAAGCTGCGTAAGCGGCTCCTCCGCGATGCCGATGACCTGCAGAAGCGCCTGGCAGTGTACCAGGCCGGGGCGAAT

>Seq227 [organism=Homo sapiens] Control ApoE ε3ε3

GACGTAGCGGGGCGCGGAATGGAGGACGTGTGCGGCCGCCTGGTGCAGTACCGCGGCGAGGTGCAGGCCATGCTCGGCCAGAGCACCGAGGAGCTGCGGGTGCGCCTCGCCTCCCACCTGCGCAAGCTGCGTAAGCGGCTCCTCCGCGATGCCGATGACCTGCAGAAGCGCCTGGCAGTGTACCAGGCCGGGGCGAAT

>Seq228 [organism=Homo sapiens] Control ApoE ε3ε3

GACGTAGTGGGGCGCGGAATGGAGGACGTGTGCGGCCGCCTGGTGCAGTACCGCGGCGAGGTGCAGGCCATGCTCGGCCAGAGCACCGAGGAGCTGCGGGTGCGCCTCGCCTCCCACCTGCGCAAGCTGCGTAAGCGGCTCCTCCGCGATGCCGATGACCTGCAGAAGCGCCTGGCAGTGTACCAGGCCGGGGCGAAT

>Seq229 [organism=Homo sapiens] Control ApoE ε3ε3

GACGTAGCGGGGCGCGGAATGGAGGACGTGTGCGGCCGCCTGGTGCAGTACCGCGGCGAGGTGCAGGCCATGCTCGGCCAGAGCACCGAGGAGCTGCGGGTGCGCCTCGCCTCCCACCTGCGCAAGCTGCGTAAGCGGCTCCTCCGCGATGCCGATGACCTGCAGAAGCGCCTGGCAGTGTACCAGGCCGGGGCGAAT

>Seq230 [organism=Homo sapiens] Control ApoE ε3ε3

GACGTAGGGGGGCGCGGAATGGAGGACGTGTGCGGCCGCCTGGTGCAGTACCGCGGCGAGGTGCAGGCCATGCTCGGCCAGAGCACCGAGGAGCTGCGGGTGCGCCTCGCCTCCCACCTGCGCAAGCTGCGTAAGCGGCTCCTCCGCGATGCCGATGACCTGCAGAAGCGCCTGGCAGTGTACCAGGCCGGGGCGAAT

>Seq231 [organism=Homo sapiens] Control ApoE ε3ε3

GACGTAGTGGGGCGCGGAATGGAGGACGTGTGCGGCCGCCTGGTGCAGTACCGCGGCGAGGTGCAGGCCATGCTCGGCCAGAGCACCGAGGAGCTGCGGGTGCGCCTCGCCTCCCACCTGCGCAAGCTGCGTAAGCGGCTCCTCCGCGATGCCGATGACCTGCAGAAGCGCCTGGCAGTGTACCAGGCCGGGGCGAAT

>Seq232 [organism=Homo sapiens] Control ApoE ε3ε3

GACGTAGCGGGGCGCGGAATGGAGGACGTGTGCGGCCGCCTGGTGCAGTACCGCGGCGAGGTGCAGGCCATGCTCGGCCAGAGCACCGAGGAGCTGCGGGTGCGCCTCGCCTCCCACCTGCGCAAGCTGCGTAAGCGGCTCCTCCGCGATGCCGATGACCTGCAGAAGCGCCTGGCAGTGTACCAGGCCGGGGCGAAT

>Seq233 [organism=Homo sapiens] Control ApoE ε3ε3

ATCGTAGCGGGGCGCGGAATGGAGGACGTGTGCGGCCGCCTGGTGCAGTACCGCGGCGAGGTGCAGGCCATGCTCGGCCAGAGCACCGAGGAGCTGCGGGTGCGCCTCGCCTCCCACCTGCGCAAGCTGCGTAAGCGGCTCCTCCGCGATGCCGATGACCTGCAGAAGCGCCTGGCAGTGTACCAGGCCGGGGCGAAT

>Seq234 [organism=Homo sapiens] Control ApoE ε3ε3

GACCTAGCGGGGCGCGGAATGGAGGACGTGTGCGGCCGCCTGGTGCAGTACCGCGGCGAGGTGCAGGCCATGCTCGGCCAGAGCACCGAGGAGCTGCGGGTGCGCCTCGCCTCCCACCTGCGCAAGCTGCGTAAGCGGCTCCTCCGCGATGCCGATGACCTGCAGAAGCGCCTGGCAGTGTACCAGGCCGGGGCGAAT

>Seq235 [organism=Homo sapiens] Control ApoE ε3ε3

GACGTTCGGGGGCGCGGAATGGAGGACGTGTGCGGCCGCCTGGTGCAGTACCGCGGCGAGGTGCAGGCCATGCTCGGCCAGAGCACCGAGGAGCTGCGGGTGCGCCTCGCCTCCCACCTGCGCAAGCTGCGTAAGCGGCTCCTCCGCGATGCCGATGACCTGCAGAAGCGCCTGGCAGTGTACCAGGCCGGGGCGAAT

>Seq236 [organism=Homo sapiens] Control ApoE ε3ε3

GACGTAGTGGGGCGCGGAATGGAGGACGTGTGCGGCCGCCTGGTGCAGTACCGCGGCGAGGTGCAGGCCATGCTCGGCCAGAGCACCGAGGAGCTGCGGGTGCGCCTCGCCTCCCACCTGCGCAAGCTGCGTAAGCGGCTCCTCCGCGATGCCGATGACCTGCAGAAGCGCCTGGCAGTGTACCAGGCCGGGGCGAAT

>Seq237 [organism=Homo sapiens] Control ApoE ε3ε3

GACGTAGCGGGGCGCGGAATGGAGGACGTGTGCGGCCGCCTGGTGCAGTACCGCGGCGAGGTGCAGGCCATGCTCGGCCAGAGCACCGAGGAGCTGCGGGTGCGCCTCGCCTCCCACCTGCGCAAGCTGCGTAAGCGGCTCCTCCGCGATGCCGATGACCTGCAGAAGCGCCTGGCAGTGTACCAGGCCGGGGCGAAT

>Seq238 [organism=Homo sapiens] Control ApoE ε3ε3

GTCGTAGCGGGGCGCGGAATGGAGGACGTGTGCGGCCGCCTGGTGCAGTACCGCGGCGAGGTGCAGGCCATGCTCGGCCAGAGCACCGAGGAGCTGCGGGTGCGCCTCGCCTCCCACCTGCGCAAGCTGCGTAAGCGGCTCCTCCGCGATGCCGATGACCTGCAGAAGCGCCTGGCAGTGTACCAGGCCGGGGCGAAT

>Seq239 [organism=Homo sapiens] Control ApoE ε3ε3

GACATAGCGGGGCGCGGAATGGAGGACGTGTGCGGCCGCCTGGTGCAGTACCGCGGCGAGGTGCAGGCCATGCTCGGCCAGAGCACCGAGGAGCTGCGGGTGCGCCTCGCCTCCCACCTGCGCAAGCTGCGTAAGCGGCTCCTCCGCGATGCCGATGACCTGCAGAAGCGCCTGGCAGTGTACCAGGCCGGGGCGAAT

>Seq240 [organism=Homo sapiens] Control ApoE ε3ε3

GACGGAGCGGGGCGCGGAATGGAGGACGTGTGCGGCCGCCTGGTGCAGTACCGCGGCGAGGTGCAGGCCATGCTCGGCCAGAGCACCGAGGAGCTGCGGGTGCGCCTCGCCTCCCACCTGCGCAAGCTGCGTAAGCGGCTCCTCCGCGATGCCGATGACCTGCAGAAGCGCCTGGCAGTGTACCAGGCCGGGGCGAAT

>Seq241 [organism=Homo sapiens] Control ApoE ε3ε3

GACGTAGGGGGGCGCGGAATGGAGGACGTGTGCGGCCGCCTGGTGCAGTACCGCGGCGAGGTGCAGGCCATGCTCGGCCAGAGCACCGAGGAGCTGCGGGTGCGCCTCGCCTCCCACCTGCGCAAGCTGCGTAAGCGGCTCCTCCGCGATGCCGATGACCTGCAGAAGCGCCTGGCAGTGTACCAGGCCGGGGCGAAT

>Seq242 [organism=Homo sapiens] Control ApoE ε3ε3

GACGTAGTGGGGCGCGGAATGGAGGACGTGTGCGGCCGCCTGGTGCAGTACCGCGGCGAGGTGCAGGCCATGCTCGGCCAGAGCACCGAGGAGCTGCGGGTGCGCCTCGCCTCCCACCTGCGCAAGCTGCGTAAGCGGCTCCTCCGCGATGCCGATGACCTGCAGAAGCGCCTGGCAGTGTACCAGGCCGGGGCGAAT

>Seq243 [organism=Homo sapiens] Control ApoE ε3ε3

GACGTAGCGGGGCGCGGAATGGAGGACGTGTGCGGCCGCCTGGTGCAGTACCGCGGCGAGGTGCAGGCCATGCTCGGCCAGAGCACCGAGGAGCTGCGGGTGCGCCTCGCCTCCCACCTGCGCAAGCTGCGTAAGCGGCTCCTCCGCGATGCCGATGACCTGCAGAAGCGCCTGGCAGTGTACCAGGCCGGGGCGAAT

>Seq244 [organism=Homo sapiens] Control ApoE ε3ε3

GACGTAGTGGGGCGCGGAATGGAGGACGTGTGCGGCCGCCTGGTGCAGTACCGCGGCGAGGTGCAGGCCATGCTCGGCCAGAGCACCGAGGAGCTGCGGGTGCGCCTCGCCTCCCACCTGCGCAAGCTGCGTAAGCGGCTCCTCCGCGATGCCGATGACCTGCAGAAGCGCCTGGCAGTGTACCAGGCCGGGGCGAAT

>Seq245 [organism=Homo sapiens] Control ApoE ε3ε3

GACGTAGCGGGGCGCGGAATGGAGGACGTGTGCGGCCGCCTGGTGCAGTACCGCGGCGAGGTGCAGGCCATGCTCGGCCAGAGCACCGAGGAGCTGCGGGTGCGCCTCGCCTCCCACCTGCGCAAGCTGCGTAAGCGGCTCCTCCGCGATGCCGATGACCTGCAGAAGCGCCTGGCAGTGTACCAGGCCGGGGCGAAT

>Seq246 [organism=Homo sapiens] Control ApoE ε3ε3

GACGTAGGGGGGCGCGGAATGGAGGACGTGTGCGGCCGCCTGGTGCAGTACCGCGGCGAGGTGCAGGCCATGCTCGGCCAGAGCACCGAGGAGCTGCGGGTGCGCCTCGCCTCCCACCTGCGCAAGCTGCGTAAGCGGCTCCTCCGCGATGCCGATGACCTGCAGAAGCGCCTGGCAGTGTACCAGGCCGGGGCGAAT

>Seq247 [organism=Homo sapiens] Control ApoE ε3ε3

GACGTAGCGGGGCGCGGAATGGAGGACGTGTGCGGCCGCCTGGTGCAGTACCGCGGCGAGGTGCAGGCCATGCTCGGCCAGAGCACCGAGGAGCTGCGGGTGCGCCTCGCCTCCCACCTGCGCAAGCTGCGTAAGCGGCTCCTCCGCGATGCCGATGACCTGCAGAAGCGCCTGGCAGTGTACCAGGCCGGGGCGAAT

>Seq248 [organism=Homo sapiens] Control ApoE ε3ε3

GACGTAGTGGGGCGCGGAATGGAGGACGTGTGCGGCCGCCTGGTGCAGTACCGCGGCGAGGTGCAGGCCATGCTCGGCCAGAGCACCGAGGAGCTGCGGGTGCGCCTCGCCTCCCACCTGCGCAAGCTGCGTAAGCGGCTCCTCCGCGATGCCGATGACCTGCAGAAGCGCCTGGCAGTGTACCAGGCCGGGGCGAAT

>Seq249 [organism=Homo sapiens] Control ApoE ε3ε3

GACGTAGCGGGGCGCGGAATGGAGGACGTGTGCGGCCGCCTGGTGCAGTACCGCGGCGAGGTGCAGGCCATGCTCGGCCAGAGCACCGAGGAGCTGCGGGTGCGCCTCGCCTCCCACCTGCGCAAGCTGCGTAAGCGGCTCCTCCGCGATGCCGATGACCTGCAGAAGCGCCTGGCAGTGTACCAGGCCGGGGCGAAT

>Seq250 [organism=Homo sapiens] Control ApoE ε3ε3

GACGTAGGGGGGCGCGGAATGGAGGACGTGTGCGGCCGCCTGGTGCAGTACCGCGGCGAGGTGCAGGCCATGCTCGGCCAGAGCACCGAGGAGCTGCGGGTGCGCCTCGCCTCCCACCTGCGCAAGCTGCGTAAGCGGCTCCTCCGCGATGCCGATGACCTGCAGAAGCGCCTGGCAGTGTACCAGGCCGGGGCGAAT

>Seq251 [organism=Homo sapiens] Control ApoE ε3ε3

GACGTAGTGGGGCGCGGAATGGAGGACGTGTGCGGCCGCCTGGTGCAGTACCGCGGCGAGGTGCAGGCCATGCTCGGCCAGAGCACCGAGGAGCTGCGGGTGCGCCTCGCCTCCCACCTGCGCAAGCTGCGTAAGCGGCTCCTCCGCGATGCCGATGACCTGCAGAAGCGCCTGGCAGTGTACCAGGCCGGGGCGAAT

>Seq252 [organism=Homo sapiens] Control ApoE ε3ε3

GACGTAGCGGGGCGCGGAATGGAGGACGTGTGCGGCCGCCTGGTGCAGTACCGCGGCGAGGTGCAGGCCATGCTCGGCCAGAGCACCGAGGAGCTGCGGGTGCGCCTCGCCTCCCACCTGCGCAAGCTGCGTAAGCGGCTCCTCCGCGATGCCGATGACCTGCAGAAGCGCCTGGCAGTGTACCAGGCCGGGGCGAAT

>Seq253 [organism=Homo sapiens] Control ApoE ε3ε3

ATCGTAGCGGGGCGCGGAATGGAGGACGTGTGCGGCCGCCTGGTGCAGTACCGCGGCGAGGTGCAGGCCATGCTCGGCCAGAGCACCGAGGAGCTGCGGGTGCGCCTCGCCTCCCACCTGCGCAAGCTGCGTAAGCGGCTCCTCCGCGATGCCGATGACCTGCAGAAGCGCCTGGCAGTGTACCAGGCCGGGGCGAAT

>Seq254 [organism=Homo sapiens] Control ApoE ε3ε3

GACCTAGCGGGGCGCGGAATGGAGGACGTGTGCGGCCGCCTGGTGCAGTACCGCGGCGAGGTGCAGGCCATGCTCGGCCAGAGCACCGAGGAGCTGCGGGTGCGCCTCGCCTCCCACCTGCGCAAGCTGCGTAAGCGGCTCCTCCGCGATGCCGATGACCTGCAGAAGCGCCTGGCAGTGTACCAGGCCGGGGCGAAT

>Seq255 [organism=Homo sapiens] Control ApoE ε3ε3

GACGTTCGGGGGCGCGGAATGGAGGACGTGTGCGGCCGCCTGGTGCAGTACCGCGGCGAGGTGCAGGCCATGCTCGGCCAGAGCACCGAGGAGCTGCGGGTGCGCCTCGCCTCCCACCTGCGCAAGCTGCGTAAGCGGCTCCTCCGCGATGCCGATGACCTGCAGAAGCGCCTGGCAGTGTACCAGGCCGGGGCGAAT

>Seq256 [organism=Homo sapiens] Control ApoE ε3ε3

GACGTAGTGGGGCGCGGAATGGAGGACGTGTGCGGCCGCCTGGTGCAGTACCGCGGCGAGGTGCAGGCCATGCTCGGCCAGAGCACCGAGGAGCTGCGGGTGCGCCTCGCCTCCCACCTGCGCAAGCTGCGTAAGCGGCTCCTCCGCGATGCCGATGACCTGCAGAAGCGCCTGGCAGTGTACCAGGCCGGGGCGAAT

>Seq257 [organism=Homo sapiens] Control ApoE ε3ε3

GACGTAGCGGGGCGCGGAATGGAGGACGTGTGCGGCCGCCTGGTGCAGTACCGCGGCGAGGTGCAGGCCATGCTCGGCCAGAGCACCGAGGAGCTGCGGGTGCGCCTCGCCTCCCACCTGCGCAAGCTGCGTAAGCGGCTCCTCCGCGATGCCGATGACCTGCAGAAGCGCCTGGCAGTGTACCAGGCCGGGGCGAAT

>Seq258 [organism=Homo sapiens] Control ApoE ε3ε3

GTCGTAGCGGGGCGCGGAATGGAGGACGTGTGCGGCCGCCTGGTGCAGTACCGCGGCGAGGTGCAGGCCATGCTCGGCCAGAGCACCGAGGAGCTGCGGGTGCGCCTCGCCTCCCACCTGCGCAAGCTGCGTAAGCGGCTCCTCCGCGATGCCGATGACCTGCAGAAGCGCCTGGCAGTGTACCAGGCCGGGGCGAAT

>Seq259 [organism=Homo sapiens] Control ApoE ε3ε3

GACATAGCGGGGCGCGGAATGGAGGACGTGTGCGGCCGCCTGGTGCAGTACCGCGGCGAGGTGCAGGCCATGCTCGGCCAGAGCACCGAGGAGCTGCGGGTGCGCCTCGCCTCCCACCTGCGCAAGCTGCGTAAGCGGCTCCTCCGCGATGCCGATGACCTGCAGAAGCGCCTGGCAGTGTACCAGGCCGGGGCGAAT

>Seq260 [organism=Homo sapiens] Control ApoE ε3ε3

GACGGAGCGGGGCGCGGAATGGAGGACGTGTGCGGCCGCCTGGTGCAGTACCGCGGCGAGGTGCAGGCCATGCTCGGCCAGAGCACCGAGGAGCTGCGGGTGCGCCTCGCCTCCCACCTGCGCAAGCTGCGTAAGCGGCTCCTCCGCGATGCCGATGACCTGCAGAAGCGCCTGGCAGTGTACCAGGCCGGGGCGAAT

>Seq261 [organism=Homo sapiens] Control ApoE ε3ε3

GACGTAGGGGGGCGCGGAATGGAGGACGTGTGCGGCCGCCTGGTGCAGTACCGCGGCGAGGTGCAGGCCATGCTCGGCCAGAGCACCGAGGAGCTGCGGGTGCGCCTCGCCTCCCACCTGCGCAAGCTGCGTAAGCGGCTCCTCCGCGATGCCGATGACCTGCAGAAGCGCCTGGCAGTGTACCAGGCCGGGGCGAAT

>Seq262 [organism=Homo sapiens] Control ApoE ε3ε3

GACGTAGTGGGGCGCGGAATGGAGGACGTGTGCGGCCGCCTGGTGCAGTACCGCGGCGAGGTGCAGGCCATGCTCGGCCAGAGCACCGAGGAGCTGCGGGTGCGCCTCGCCTCCCACCTGCGCAAGCTGCGTAAGCGGCTCCTCCGCGATGCCGATGACCTGCAGAAGCGCCTGGCAGTGTACCAGGCCGGGGCGAAT

>Seq263 [organism=Homo sapiens] Control ApoE ε3ε3

GACGTAGCGGGGCGCGGAATGGAGGACGTGTGCGGCCGCCTGGTGCAGTACCGCGGCGAGGTGCAGGCCATGCTCGGCCAGAGCACCGAGGAGCTGCGGGTGCGCCTCGCCTCCCACCTGCGCAAGCTGCGTAAGCGGCTCCTCCGCGATGCCGATGACCTGCAGAAGCGCCTGGCAGTGTACCAGGCCGGGGCGAAT

>Seq264 [organism=Homo sapiens] Control ApoE ε3ε3

GACGTAGTGGGGCGCGGAATGGAGGACGTGTGCGGCCGCCTGGTGCAGTACCGCGGCGAGGTGCAGGCCATGCTCGGCCAGAGCACCGAGGAGCTGCGGGTGCGCCTCGCCTCCCACCTGCGCAAGCTGCGTAAGCGGCTCCTCCGCGATGCCGATGACCTGCAGAAGCGCCTGGCAGTGTACCAGGCCGGGGCGAAT

>Seq265 [organism=Homo sapiens] Control ApoE ε3ε3

GACGTAGCGGGGCGCGGAATGGAGGACGTGTGCGGCCGCCTGGTGCAGTACCGCGGCGAGGTGCAGGCCATGCTCGGCCAGAGCACCGAGGAGCTGCGGGTGCGCCTCGCCTCCCACCTGCGCAAGCTGCGTAAGCGGCTCCTCCGCGATGCCGATGACCTGCAGAAGCGCCTGGCAGTGTACCAGGCCGGGGCGAAT

>Seq266 [organism=Homo sapiens] Control ApoE ε3ε3

GACGTAGGGGGGCGCGGAATGGAGGACGTGTGCGGCCGCCTGGTGCAGTACCGCGGCGAGGTGCAGGCCATGCTCGGCCAGAGCACCGAGGAGCTGCGGGTGCGCCTCGCCTCCCACCTGCGCAAGCTGCGTAAGCGGCTCCTCCGCGATGCCGATGACCTGCAGAAGCGCCTGGCAGTGTACCAGGCCGGGGCGAAT

>Seq267 [organism=Homo sapiens] Control ApoE ε3ε3

GACGTAGCGGGGCGCGGAATGGAGGACGTGTGCGGCCGCCTGGTGCAGTACCGCGGCGAGGTGCAGGCCATGCTCGGCCAGAGCACCGAGGAGCTGCGGGTGCGCCTCGCCTCCCACCTGCGCAAGCTGCGTAAGCGGCTCCTCCGCGATGCCGATGACCTGCAGAAGCGCCTGGCAGTGTACCAGGCCGGGGCGAAT

>Seq268 [organism=Homo sapiens] Control ApoE ε3ε3

GACGTAGTGGGGCGCGGAATGGAGGACGTGTGCGGCCGCCTGGTGCAGTACCGCGGCGAGGTGCAGGCCATGCTCGGCCAGAGCACCGAGGAGCTGCGGGTGCGCCTCGCCTCCCACCTGCGCAAGCTGCGTAAGCGGCTCCTCCGCGATGCCGATGACCTGCAGAAGCGCCTGGCAGTGTACCAGGCCGGGGCGAAT

>Seq269 [organism=Homo sapiens] Control ApoE ε3ε3

GACGTAGCGGGGCGCGGAATGGAGGACGTGTGCGGCCGCCTGGTGCAGTACCGCGGCGAGGTGCAGGCCATGCTCGGCCAGAGCACCGAGGAGCTGCGGGTGCGCCTCGCCTCCCACCTGCGCAAGCTGCGTAAGCGGCTCCTCCGCGATGCCGATGACCTGCAGAAGCGCCTGGCAGTGTACCAGGCCGGGGCGAAT

>Seq270 [organism=Homo sapiens] Control ApoE ε3ε3

GACGTAGGGGGGCGCGGAATGGAGGACGTGTGCGGCCGCCTGGTGCAGTACCGCGGCGAGGTGCAGGCCATGCTCGGCCAGAGCACCGAGGAGCTGCGGGTGCGCCTCGCCTCCCACCTGCGCAAGCTGCGTAAGCGGCTCCTCCGCGATGCCGATGACCTGCAGAAGCGCCTGGCAGTGTACCAGGCCGGGGCGAAT

>Seq271 [organism=Homo sapiens] Control ApoE ε3ε3

GACGTAGTGGGGCGCGGAATGGAGGACGTGTGCGGCCGCCTGGTGCAGTACCGCGGCGAGGTGCAGGCCATGCTCGGCCAGAGCACCGAGGAGCTGCGGGTGCGCCTCGCCTCCCACCTGCGCAAGCTGCGTAAGCGGCTCCTCCGCGATGCCGATGACCTGCAGAAGCGCCTGGCAGTGTACCAGGCCGGGGCGAAT

>Seq272 [organism=Homo sapiens] Control ApoE ε3ε3

GACGTAGCGGGGCGCGGAATGGAGGACGTGTGCGGCCGCCTGGTGCAGTACCGCGGCGAGGTGCAGGCCATGCTCGGCCAGAGCACCGAGGAGCTGCGGGTGCGCCTCGCCTCCCACCTGCGCAAGCTGCGTAAGCGGCTCCTCCGCGATGCCGATGACCTGCAGAAGCGCCTGGCAGTGTACCAGGCCGGGGCGAAT

>Seq273 [organism=Homo sapiens] Control ApoE ε3ε3

ATCGTAGCGGGGCGCGGAATGGAGGACGTGTGCGGCCGCCTGGTGCAGTACCGCGGCGAGGTGCAGGCCATGCTCGGCCAGAGCACCGAGGAGCTGCGGGTGCGCCTCGCCTCCCACCTGCGCAAGCTGCGTAAGCGGCTCCTCCGCGATGCCGATGACCTGCAGAAGCGCCTGGCAGTGTACCAGGCCGGGGCGAAT

>Seq274 [organism=Homo sapiens] Control ApoE ε3ε3

GACCTAGCGGGGCGCGGAATGGAGGACGTGTGCGGCCGCCTGGTGCAGTACCGCGGCGAGGTGCAGGCCATGCTCGGCCAGAGCACCGAGGAGCTGCGGGTGCGCCTCGCCTCCCACCTGCGCAAGCTGCGTAAGCGGCTCCTCCGCGATGCCGATGACCTGCAGAAGCGCCTGGCAGTGTACCAGGCCGGGGCGAAT

>Seq275 [organism=Homo sapiens] Control ApoE ε3ε3

GACGTTCGGGGGCGCGGAATGGAGGACGTGTGCGGCCGCCTGGTGCAGTACCGCGGCGAGGTGCAGGCCATGCTCGGCCAGAGCACCGAGGAGCTGCGGGTGCGCCTCGCCTCCCACCTGCGCAAGCTGCGTAAGCGGCTCCTCCGCGATGCCGATGACCTGCAGAAGCGCCTGGCAGTGTACCAGGCCGGGGCGAAT

>Seq276 [organism=Homo sapiens] Control ApoE ε3ε3

GACGTAGTGGGGCGCGGAATGGAGGACGTGTGCGGCCGCCTGGTGCAGTACCGCGGCGAGGTGCAGGCCATGCTCGGCCAGAGCACCGAGGAGCTGCGGGTGCGCCTCGCCTCCCACCTGCGCAAGCTGCGTAAGCGGCTCCTCCGCGATGCCGATGACCTGCAGAAGCGCCTGGCAGTGTACCAGGCCGGGGCGAAT

>Seq277 [organism=Homo sapiens] Control ApoE ε3ε3

GACGTAGCGGGGCGCGGAATGGAGGACGTGTGCGGCCGCCTGGTGCAGTACCGCGGCGAGGTGCAGGCCATGCTCGGCCAGAGCACCGAGGAGCTGCGGGTGCGCCTCGCCTCCCACCTGCGCAAGCTGCGTAAGCGGCTCCTCCGCGATGCCGATGACCTGCAGAAGCGCCTGGCAGTGTACCAGGCCGGGGCGAAT

>Seq278 [organism=Homo sapiens] Control ApoE ε3ε3

GTCGTAGCGGGGCGCGGAATGGAGGACGTGTGCGGCCGCCTGGTGCAGTACCGCGGCGAGGTGCAGGCCATGCTCGGCCAGAGCACCGAGGAGCTGCGGGTGCGCCTCGCCTCCCACCTGCGCAAGCTGCGTAAGCGGCTCCTCCGCGATGCCGATGACCTGCAGAAGCGCCTGGCAGTGTACCAGGCCGGGGCGAAT

>Seq279 [organism=Homo sapiens] Control ApoE ε3ε3

GACATAGCGGGGCGCGGAATGGAGGACGTGTGCGGCCGCCTGGTGCAGTACCGCGGCGAGGTGCAGGCCATGCTCGGCCAGAGCACCGAGGAGCTGCGGGTGCGCCTCGCCTCCCACCTGCGCAAGCTGCGTAAGCGGCTCCTCCGCGATGCCGATGACCTGCAGAAGCGCCTGGCAGTGTACCAGGCCGGGGCGAAT

>Seq280 [organism=Homo sapiens] Control ApoE ε3ε3

GACGGAGCGGGGCGCGGAATGGAGGACGTGTGCGGCCGCCTGGTGCAGTACCGCGGCGAGGTGCAGGCCATGCTCGGCCAGAGCACCGAGGAGCTGCGGGTGCGCCTCGCCTCCCACCTGCGCAAGCTGCGTAAGCGGCTCCTCCGCGATGCCGATGACCTGCAGAAGCGCCTGGCAGTGTACCAGGCCGGGGCGAAT

>Seq281 [organism=Homo sapiens] Control ApoE ε3ε3

GACGTAGGGGGGCGCGGAATGGAGGACGTGTGCGGCCGCCTGGTGCAGTACCGCGGCGAGGTGCAGGCCATGCTCGGCCAGAGCACCGAGGAGCTGCGGGTGCGCCTCGCCTCCCACCTGCGCAAGCTGCGTAAGCGGCTCCTCCGCGATGCCGATGACCTGCAGAAGCGCCTGGCAGTGTACCAGGCCGGGGCGAAT

>Seq282 [organism=Homo sapiens] Control ApoE ε3ε3

GACGTAGTGGGGCGCGGAATGGAGGACGTGTGCGGCCGCCTGGTGCAGTACCGCGGCGAGGTGCAGGCCATGCTCGGCCAGAGCACCGAGGAGCTGCGGGTGCGCCTCGCCTCCCACCTGCGCAAGCTGCGTAAGCGGCTCCTCCGCGATGCCGATGACCTGCAGAAGCGCCTGGCAGTGTACCAGGCCGGGGCGAAT

>Seq283 [organism=Homo sapiens] Control ApoE ε3ε3

GACGTAGCGGGGCGCGGAATGGAGGACGTGTGCGGCCGCCTGGTGCAGTACCGCGGCGAGGTGCAGGCCATGCTCGGCCAGAGCACCGAGGAGCTGCGGGTGCGCCTCGCCTCCCACCTGCGCAAGCTGCGTAAGCGGCTCCTCCGCGATGCCGATGACCTGCAGAAGCGCCTGGCAGTGTACCAGGCCGGGGCGAAT

>Seq284 [organism=Homo sapiens] Control ApoE ε3ε3

GACGTAGTGGGGCGCGGAATGGAGGACGTGTGCGGCCGCCTGGTGCAGTACCGCGGCGAGGTGCAGGCCATGCTCGGCCAGAGCACCGAGGAGCTGCGGGTGCGCCTCGCCTCCCACCTGCGCAAGCTGCGTAAGCGGCTCCTCCGCGATGCCGATGACCTGCAGAAGCGCCTGGCAGTGTACCAGGCCGGGGCGAAT

>Seq285 [organism=Homo sapiens] Control ApoE ε3ε3

GACGTAGCGGGGCGCGGAATGGAGGACGTGTGCGGCCGCCTGGTGCAGTACCGCGGCGAGGTGCAGGCCATGCTCGGCCAGAGCACCGAGGAGCTGCGGGTGCGCCTCGCCTCCCACCTGCGCAAGCTGCGTAAGCGGCTCCTCCGCGATGCCGATGACCTGCAGAAGCGCCTGGCAGTGTACCAGGCCGGGGCGAAT

>Seq286 [organism=Homo sapiens] Control ApoE ε3ε3

GACGTAGGGGGGCGCGGAATGGAGGACGTGTGCGGCCGCCTGGTGCAGTACCGCGGCGAGGTGCAGGCCATGCTCGGCCAGAGCACCGAGGAGCTGCGGGTGCGCCTCGCCTCCCACCTGCGCAAGCTGCGTAAGCGGCTCCTCCGCGATGCCGATGACCTGCAGAAGCGCCTGGCAGTGTACCAGGCCGGGGCGAAT

>Seq287 [organism=Homo sapiens] Control ApoE ε3ε3

GACGTAGCGGGGCGCGGAATGGAGGACGTGTGCGGCCGCCTGGTGCAGTACCGCGGCGAGGTGCAGGCCATGCTCGGCCAGAGCACCGAGGAGCTGCGGGTGCGCCTCGCCTCCCACCTGCGCAAGCTGCGTAAGCGGCTCCTCCGCGATGCCGATGACCTGCAGAAGCGCCTGGCAGTGTACCAGGCCGGGGCGAAT

>Seq288 [organism=Homo sapiens] Control ApoE ε3ε3

GACGTAGTGGGGCGCGGAATGGAGGACGTGTGCGGCCGCCTGGTGCAGTACCGCGGCGAGGTGCAGGCCATGCTCGGCCAGAGCACCGAGGAGCTGCGGGTGCGCCTCGCCTCCCACCTGCGCAAGCTGCGTAAGCGGCTCCTCCGCGATGCCGATGACCTGCAGAAGCGCCTGGCAGTGTACCAGGCCGGGGCGAAT

>Seq289 [organism=Homo sapiens] Control ApoE ε3ε3

GACGTAGCGGGGCGCGGAATGGAGGACGTGTGCGGCCGCCTGGTGCAGTACCGCGGCGAGGTGCAGGCCATGCTCGGCCAGAGCACCGAGGAGCTGCGGGTGCGCCTCGCCTCCCACCTGCGCAAGCTGCGTAAGCGGCTCCTCCGCGATGCCGATGACCTGCAGAAGCGCCTGGCAGTGTACCAGGCCGGGGCGAAT

>Seq290 [organism=Homo sapiens] Control ApoE ε3ε3

GACGTAGGGGGGCGCGGAATGGAGGACGTGTGCGGCCGCCTGGTGCAGTACCGCGGCGAGGTGCAGGCCATGCTCGGCCAGAGCACCGAGGAGCTGCGGGTGCGCCTCGCCTCCCACCTGCGCAAGCTGCGTAAGCGGCTCCTCCGCGATGCCGATGACCTGCAGAAGCGCCTGGCAGTGTACCAGGCCGGGGCGAAT

>Seq291 [organism=Homo sapiens] Control ApoE ε3ε3

GACGTAGTGGGGCGCGGAATGGAGGACGTGTGCGGCCGCCTGGTGCAGTACCGCGGCGAGGTGCAGGCCATGCTCGGCCAGAGCACCGAGGAGCTGCGGGTGCGCCTCGCCTCCCACCTGCGCAAGCTGCGTAAGCGGCTCCTCCGCGATGCCGATGACCTGCAGAAGCGCCTGGCAGTGTACCAGGCCGGGGCGAAT

>Seq292 [organism=Homo sapiens] Control ApoE ε3ε3

GACGTAGCGGGGCGCGGAATGGAGGACGTGTGCGGCCGCCTGGTGCAGTACCGCGGCGAGGTGCAGGCCATGCTCGGCCAGAGCACCGAGGAGCTGCGGGTGCGCCTCGCCTCCCACCTGCGCAAGCTGCGTAAGCGGCTCCTCCGCGATGCCGATGACCTGCAGAAGCGCCTGGCAGTGTACCAGGCCGGGGCGAAT

>Seq293 [organism=Homo sapiens] Control ApoE ε3ε3 ATCGTAGCGGGGCGCGGAATGGAGGACGTGTGCGGCCGCCTGGTGCAGTACCGCGGCGAGGTGCAGGCCATGCTCGGCCAGAGCACCGAGGAGCTGCGGGTGCGCCTCGCCTCCCACCTGCGCAAGCTGCGTAAGCGGCTCCTCCGCGATGCCGATGACCTGCAGAAGCGCCTGGCAGTGTACCAGGCCGGGGCGAAT

>Seq294 [organism=Homo sapiens] Control ApoE ε3ε3

GACCTAGCGGGGCGCGGAATGGAGGACGTGTGCGGCCGCCTGGTGCAGTACCGCGGCGAGGTGCAGGCCATGCTCGGCCAGAGCACCGAGGAGCTGCGGGTGCGCCTCGCCTCCCACCTGCGCAAGCTGCGTAAGCGGCTCCTCCGCGATGCCGATGACCTGCAGAAGCGCCTGGCAGTGTACCAGGCCGGGGCGAAT

>Seq295 [organism=Homo sapiens] Control ApoE ε3ε3

GACGTTCGGGGGCGCGGAATGGAGGACGTGTGCGGCCGCCTGGTGCAGTACCGCGGCGAGGTGCAGGCCATGCTCGGCCAGAGCACCGAGGAGCTGCGGGTGCGCCTCGCCTCCCACCTGCGCAAGCTGCGTAAGCGGCTCCTCCGCGATGCCGATGACCTGCAGAAGCGCCTGGCAGTGTACCAGGCCGGGGCGAAT

>Seq296 [organism=Homo sapiens] Control ApoE ε3ε3

GACGTAGTGGGGCGCGGAATGGAGGACGTGTGCGGCCGCCTGGTGCAGTACCGCGGCGAGGTGCAGGCCATGCTCGGCCAGAGCACCGAGGAGCTGCGGGTGCGCCTCGCCTCCCACCTGCGCAAGCTGCGTAAGCGGCTCCTCCGCGATGCCGATGACCTGCAGAAGCGCCTGGCAGTGTACCAGGCCGGGGCGAAT

>Seq297 [organism=Homo sapiens] Control ApoE ε3ε3

GACGTAGCGGGGCGCGGAATGGAGGACGTGTGCGGCCGCCTGGTGCAGTACCGCGGCGAGGTGCAGGCCATGCTCGGCCAGAGCACCGAGGAGCTGCGGGTGCGCCTCGCCTCCCACCTGCGCAAGCTGCGTAAGCGGCTCCTCCGCGATGCCGATGACCTGCAGAAGCGCCTGGCAGTGTACCAGGCCGGGGCGAAT

>Seq298 [organism=Homo sapiens] Control ApoE ε3ε3

GTCGTAGCGGGGCGCGGAATGGAGGACGTGTGCGGCCGCCTGGTGCAGTACCGCGGCGAGGTGCAGGCCATGCTCGGCCAGAGCACCGAGGAGCTGCGGGTGCGCCTCGCCTCCCACCTGCGCAAGCTGCGTAAGCGGCTCCTCCGCGATGCCGATGACCTGCAGAAGCGCCTGGCAGTGTACCAGGCCGGGGCGAAT

>Seq299 [organism=Homo sapiens] Control ApoE ε3ε3

GACATAGCGGGGCGCGGAATGGAGGACGTGTGCGGCCGCCTGGTGCAGTACCGCGGCGAGGTGCAGGCCATGCTCGGCCAGAGCACCGAGGAGCTGCGGGTGCGCCTCGCCTCCCACCTGCGCAAGCTGCGTAAGCGGCTCCTCCGCGATGCCGATGACCTGCAGAAGCGCCTGGCAGTGTACCAGGCCGGGGCGAAT

>Seq300 [organism=Homo sapiens] Control ApoE ε3ε3

GACGGAGCGGGGCGCGGAATGGAGGACGTGTGCGGCCGCCTGGTGCAGTACCGCGGCGAGGTGCAGGCCATGCTCGGCCAGAGCACCGAGGAGCTGCGGGTGCGCCTCGCCTCCCACCTGCGCAAGCTGCGTAAGCGGCTCCTCCGCGATGCCGATGACCTGCAGAAGCGCCTGGCAGTGTACCAGGCCGGGGCGAAT

>Seq301 [organism=Homo sapiens] Control ApoE ε3ε3

GACGTAGGGGGGCGCGGAATGGAGGACGTGTGCGGCCGCCTGGTGCAGTACCGCGGCGAGGTGCAGGCCATGCTCGGCCAGAGCACCGAGGAGCTGCGGGTGCGCCTCGCCTCCCACCTGCGCAAGCTGCGTAAGCGGCTCCTCCGCGATGCCGATGACCTGCAGAAGCGCCTGGCAGTGTACCAGGCCGGGGCGAAT

>Seq302 [organism=Homo sapiens] Control ApoE ε3ε3

GACGTAGTGGGGCGCGGAATGGAGGACGTGTGCGGCCGCCTGGTGCAGTACCGCGGCGAGGTGCAGGCCATGCTCGGCCAGAGCACCGAGGAGCTGCGGGTGCGCCTCGCCTCCCACCTGCGCAAGCTGCGTAAGCGGCTCCTCCGCGATGCCGATGACCTGCAGAAGCGCCTGGCAGTGTACCAGGCCGGGGCGAAT

>Seq303 [organism=Homo sapiens] Control ApoE ε3ε3

GACGTAGCGGGGCGCGGAATGGAGGACGTGTGCGGCCGCCTGGTGCAGTACCGCGGCGAGGTGCAGGCCATGCTCGGCCAGAGCACCGAGGAGCTGCGGGTGCGCCTCGCCTCCCACCTGCGCAAGCTGCGTAAGCGGCTCCTCCGCGATGCCGATGACCTGCAGAAGCGCCTGGCAGTGTACCAGGCCGGGGCGAAT

>Seq304 [organism=Homo sapiens] Control ApoE ε3ε3

GACGTAGTGGGGCGCGGAATGGAGGACGTGTGCGGCCGCCTGGTGCAGTACCGCGGCGAGGTGCAGGCCATGCTCGGCCAGAGCACCGAGGAGCTGCGGGTGCGCCTCGCCTCCCACCTGCGCAAGCTGCGTAAGCGGCTCCTCCGCGATGCCGATGACCTGCAGAAGCGCCTGGCAGTGTACCAGGCCGGGGCGAAT

>Seq305 [organism=Homo sapiens] Control ApoE ε3ε3

GACGTAGCGGGGCGCGGAATGGAGGACGTGTGCGGCCGCCTGGTGCAGTACCGCGGCGAGGTGCAGGCCATGCTCGGCCAGAGCACCGAGGAGCTGCGGGTGCGCCTCGCCTCCCACCTGCGCAAGCTGCGTAAGCGGCTCCTCCGCGATGCCGATGACCTGCAGAAGCGCCTGGCAGTGTACCAGGCCGGGGCGAAT

>Seq306 [organism=Homo sapiens] Control ApoE ε3ε3

GACGTAGGGGGGCGCGGAATGGAGGACGTGTGCGGCCGCCTGGTGCAGTACCGCGGCGAGGTGCAGGCCATGCTCGGCCAGAGCACCGAGGAGCTGCGGGTGCGCCTCGCCTCCCACCTGCGCAAGCTGCGTAAGCGGCTCCTCCGCGATGCCGATGACCTGCAGAAGCGCCTGGCAGTGTACCAGGCCGGGGCGAAT

>Seq307 [organism=Homo sapiens] Control ApoE ε3ε3

GACGTAGCGGGGCGCGGAATGGAGGACGTGTGCGGCCGCCTGGTGCAGTACCGCGGCGAGGTGCAGGCCATGCTCGGCCAGAGCACCGAGGAGCTGCGGGTGCGCCTCGCCTCCCACCTGCGCAAGCTGCGTAAGCGGCTCCTCCGCGATGCCGATGACCTGCAGAAGCGCCTGGCAGTGTACCAGGCCGGGGCGAAT

>Seq308 [organism=Homo sapiens] Control ApoE ε3ε3

GACGTAGTGGGGCGCGGAATGGAGGACGTGTGCGGCCGCCTGGTGCAGTACCGCGGCGAGGTGCAGGCCATGCTCGGCCAGAGCACCGAGGAGCTGCGGGTGCGCCTCGCCTCCCACCTGCGCAAGCTGCGTAAGCGGCTCCTCCGCGATGCCGATGACCTGCAGAAGCGCCTGGCAGTGTACCAGGCCGGGGCGAAT

>Seq309 [organism=Homo sapiens] Control ApoE ε3ε3

GACGTAGCGGGGCGCGGAATGGAGGACGTGTGCGGCCGCCTGGTGCAGTACCGCGGCGAGGTGCAGGCCATGCTCGGCCAGAGCACCGAGGAGCTGCGGGTGCGCCTCGCCTCCCACCTGCGCAAGCTGCGTAAGCGGCTCCTCCGCGATGCCGATGACCTGCAGAAGCGCCTGGCAGTGTACCAGGCCGGGGCGAAT

>Seq310 [organism=Homo sapiens] Control ApoE ε3ε3

GACGTAGGGGGGCGCGGAATGGAGGACGTGTGCGGCCGCCTGGTGCAGTACCGCGGCGAGGTGCAGGCCATGCTCGGCCAGAGCACCGAGGAGCTGCGGGTGCGCCTCGCCTCCCACCTGCGCAAGCTGCGTAAGCGGCTCCTCCGCGATGCCGATGACCTGCAGAAGCGCCTGGCAGTGTACCAGGCCGGGGCGAAT

>Seq311 [organism=Homo sapiens] Control ApoE ε3ε3

GACGTAGTGGGGCGCGGAATGGAGGACGTGTGCGGCCGCCTGGTGCAGTACCGCGGCGAGGTGCAGGCCATGCTCGGCCAGAGCACCGAGGAGCTGCGGGTGCGCCTCGCCTCCCACCTGCGCAAGCTGCGTAAGCGGCTCCTCCGCGATGCCGATGACCTGCAGAAGCGCCTGGCAGTGTACCAGGCCGGGGCGAAT

>Seq312 [organism=Homo sapiens] Control ApoE ε3ε3

GACGTAGCGGGGCGCGGAATGGAGGACGTGTGCGGCCGCCTGGTGCAGTACCGCGGCGAGGTGCAGGCCATGCTCGGCCAGAGCACCGAGGAGCTGCGGGTGCGCCTCGCCTCCCACCTGCGCAAGCTGCGTAAGCGGCTCCTCCGCGATGCCGATGACCTGCAGAAGCGCCTGGCAGTGTACCAGGCCGGGGCGAAT

>Seq313 [organism=Homo sapiens] Control ApoE ε3ε3

ATCGTAGCGGGGCGCGGAATGGAGGACGTGTGCGGCCGCCTGGTGCAGTACCGCGGCGAGGTGCAGGCCATGCTCGGCCAGAGCACCGAGGAGCTGCGGGTGCGCCTCGCCTCCCACCTGCGCAAGCTGCGTAAGCGGCTCCTCCGCGATGCCGATGACCTGCAGAAGCGCCTGGCAGTGTACCAGGCCGGGGCGAAT

>Seq314 [organism=Homo sapiens] Control ApoE ε3ε3

GACCTAGCGGGGCGCGGAATGGAGGACGTGTGCGGCCGCCTGGTGCAGTACCGCGGCGAGGTGCAGGCCATGCTCGGCCAGAGCACCGAGGAGCTGCGGGTGCGCCTCGCCTCCCACCTGCGCAAGCTGCGTAAGCGGCTCCTCCGCGATGCCGATGACCTGCAGAAGCGCCTGGCAGTGTACCAGGCCGGGGCGAAT

>Seq315 [organism=Homo sapiens] Control ApoE ε3ε3

GACGTTCGGGGGCGCGGAATGGAGGACGTGTGCGGCCGCCTGGTGCAGTACCGCGGCGAGGTGCAGGCCATGCTCGGCCAGAGCACCGAGGAGCTGCGGGTGCGCCTCGCCTCCCACCTGCGCAAGCTGCGTAAGCGGCTCCTCCGCGATGCCGATGACCTGCAGAAGCGCCTGGCAGTGTACCAGGCCGGGGCGAAT

>Seq316 [organism=Homo sapiens] Control ApoE ε3ε3

GACGTAGTGGGGCGCGGAATGGAGGACGTGTGCGGCCGCCTGGTGCAGTACCGCGGCGAGGTGCAGGCCATGCTCGGCCAGAGCACCGAGGAGCTGCGGGTGCGCCTCGCCTCCCACCTGCGCAAGCTGCGTAAGCGGCTCCTCCGCGATGCCGATGACCTGCAGAAGCGCCTGGCAGTGTACCAGGCCGGGGCGAAT

>Seq317 [organism=Homo sapiens] Control ApoE ε3ε3

GACGTAGCGGGGCGCGGAATGGAGGACGTGTGCGGCCGCCTGGTGCAGTACCGCGGCGAGGTGCAGGCCATGCTCGGCCAGAGCACCGAGGAGCTGCGGGTGCGCCTCGCCTCCCACCTGCGCAAGCTGCGTAAGCGGCTCCTCCGCGATGCCGATGACCTGCAGAAGCGCCTGGCAGTGTACCAGGCCGGGGCGAAT

>Seq318 [organism=Homo sapiens] Control ApoE ε3ε3

GTCGTAGCGGGGCGCGGAATGGAGGACGTGTGCGGCCGCCTGGTGCAGTACCGCGGCGAGGTGCAGGCCATGCTCGGCCAGAGCACCGAGGAGCTGCGGGTGCGCCTCGCCTCCCACCTGCGCAAGCTGCGTAAGCGGCTCCTCCGCGATGCCGATGACCTGCAGAAGCGCCTGGCAGTGTACCAGGCCGGGGCGAAT

>Seq319 [organism=Homo sapiens] Control ApoE ε3ε3

GACATAGCGGGGCGCGGAATGGAGGACGTGTGCGGCCGCCTGGTGCAGTACCGCGGCGAGGTGCAGGCCATGCTCGGCCAGAGCACCGAGGAGCTGCGGGTGCGCCTCGCCTCCCACCTGCGCAAGCTGCGTAAGCGGCTCCTCCGCGATGCCGATGACCTGCAGAAGCGCCTGGCAGTGTACCAGGCCGGGGCGAAT

>Seq320 [organism=Homo sapiens] Control ApoE ε3ε3

GACGGAGCGGGGCGCGGAATGGAGGACGTGTGCGGCCGCCTGGTGCAGTACCGCGGCGAGGTGCAGGCCATGCTCGGCCAGAGCACCGAGGAGCTGCGGGTGCGCCTCGCCTCCCACCTGCGCAAGCTGCGTAAGCGGCTCCTCCGCGATGCCGATGACCTGCAGAAGCGCCTGGCAGTGTACCAGGCCGGGGCGAAT

>Seq321 [organism=Homo sapiens] Control ApoE ε3ε3

GACGTAGGGGGGCGCGGAATGGAGGACGTGTGCGGCCGCCTGGTGCAGTACCGCGGCGAGGTGCAGGCCATGCTCGGCCAGAGCACCGAGGAGCTGCGGGTGCGCCTCGCCTCCCACCTGCGCAAGCTGCGTAAGCGGCTCCTCCGCGATGCCGATGACCTGCAGAAGCGCCTGGCAGTGTACCAGGCCGGGGCGAAT

>Seq321 [organism=Homo sapiens] Control ApoE ε2ε4

TACGGCGGCGGCGCGGAATGGAGGACGTGTGCGGCCGCCTGGTGCAGTACCGCGGCGAGGTGCAGGCCATGCTCGGCCAGAGCACCGAGGAGCTGCGGGTGCGCCTCGCCTCCCACCTGCGCAAGCTGCGTAAGCGGCTCCTCCGCGATGCCGATGACCTGCAGAAGCGCCTGGCAGTGTACCAGGCCGGGGCGAAT

>Seq322 [organism=Homo sapiens] Control ApoE ε2ε4

GACCGCGGCGGCGCGGAATGGAGGACGTGTGCGGCCGCCTGGTGCAGTACCGCGGCGAGGTGCAGGCCATGCTCGGCCAGAGCACCGAGGAGCTGCGGGTGCGCCTCGCCTCCCACCTGCGCAAGCTGCGTAAGCGGCTCCTCCGCGATGCCGATGACCTGCAGAAGCGCCTGGCAGTGTACCAGGCCGGGGCGAAT

>Seq323 [organism=Homo sapiens] Control ApoE ε2ε4

AGCTGCGGCGGCGCGGAATGGAGGACGTGTGCGGCCGCCTGGTGCAGTACCGCGGCGAGGTGCAGGCCATGCTCGGCCAGAGCACCGAGGAGCTGCGGGTGCGCCTCGCCTCCCACCTGCGCAAGCTGCGTAAGCGGCTCCTCCGCGATGCCGATGACCTGCAGAAGCGCCTGGCAGTGTACCAGGCCGGGGCGAAT

>Seq324 [organism=Homo sapiens] Control ApoE ε2ε4

TCGTGCGGCGGCGCGGAATGGAGGACGTGTGCGGCCGCCTGGTGCAGTACCGCGGCGAGGTGCAGGCCATGCTCGGCCAGAGCACCGAGGAGCTGCGGGTGCGCCTCGCCTCCCACCTGCGCAAGCTGCGTAAGCGGCTCCTCCGCGATGCCGATGACCTGCAGAAGCGCCTGGCAGTGTACCAGGCCGGGGCGAAT

>Seq325 [organism=Homo sapiens] Control ApoE ε2ε4

GATCGCGGCGGCGCGGAATGGAGGACGTGTGCGGCCGCCTGGTGCAGTACCGCGGCGAGGTGCAGGCCATGCTCGGCCAGAGCACCGAGGAGCTGCGGGTGCGCCTCGCCTCCCACCTGCGCAAGCTGCGTAAGCGGCTCCTCCGCGATGCCGATGACCTGCAGAAGCGCCTGGCAGTGTACCAGGCCGGGGCGAAT

>Seq326 [organism=Homo sapiens] Control ApoE ε2ε4

CGTAGCGGCGGCGCGGAATGGAGGACGTGTGCGGCCGCCTGGTGCAGTACCGCGGCGAGGTGCAGGCCATGCTCGGCCAGAGCACCGAGGAGCTGCGGGTGCGCCTCGCCTCCCACCTGCGCAAGCTGCGTAAGCGGCTCCTCCGCGATGCCGATGACCTGCAGAAGCGCCTGGCAGTGTACCAGGCCGGGGCGAAT

>Seq327 [organism=Homo sapiens] Control ApoE ε2ε4

ATGCGCGGCGGCGCGGAATGGAGGACGTGTGCGGCCGCCTGGTGCAGTACCGCGGCGAGGTGCAGGCCATGCTCGGCCAGAGCACCGAGGAGCTGCGGGTGCGCCTCGCCTCCCACCTGCGCAAGCTGCGTAAGCGGCTCCTCCGCGATGCCGATGACCTGCAGAAGCGCCTGGCAGTGTACCAGGCCGGGGCGAAT

>Seq328 [organism=Homo sapiens] Control ApoE ε2ε4

GCTAGCGGCGGCGCGGAATGGAGGACGTGTGCGGCCGCCTGGTGCAGTACCGCGGCGAGGTGCAGGCCATGCTCGGCCAGAGCACCGAGGAGCTGCGGGTGCGCCTCGCCTCCCACCTGCGCAAGCTGCGTAAGCGGCTCCTCCGCGATGCCGATGACCTGCAGAAGCGCCTGGCAGTGTACCAGGCCGGGGCGAAT

>Seq329 [organism=Homo sapiens] Control ApoE ε2ε4

TGACGCGGCGGCGCGGAATGGAGGACGTGTGCGGCCGCCTGGTGCAGTACCGCGGCGAGGTGCAGGCCATGCTCGGCCAGAGCACCGAGGAGCTGCGGGTGCGCCTCGCCTCCCACCTGCGCAAGCTGCGTAAGCGGCTCCTCCGCGATGCCGATGACCTGCAGAAGCGCCTGGCAGTGTACCAGGCCGGGGCGAAT

>Seq330 [organism=Homo sapiens] Control ApoE ε2ε4

AGCTCCGGCGGCGCGGAATGGAGGACGTGTGCGGCCGCCTGGTGCAGTACCGCGGCGAGGTGCAGGCCATGCTCGGCCAGAGCACCGAGGAGCTGCGGGTGCGCCTCGCCTCCCACCTGCGCAAGCTGCGTAAGCGGCTCCTCCGCGATGCCGATGACCTGCAGAAGCGCCTGGCAGTGTACCAGGCCGGGGCGAAT

>Seq331 [organism=Homo sapiens] Control ApoE ε2ε4

GACTCCGGCGGCGCGGAATGGAGGACGTGTGCGGCCGCCTGGTGCAGTACCGCGGCGAGGTGCAGGCCATGCTCGGCCAGAGCACCGAGGAGCTGCGGGTGCGCCTCGCCTCCCACCTGCGCAAGCTGCGTAAGCGGCTCCTCCGCGATGCCGATGACCTGCAGAAGCGCCTGGCAGTGTACCAGGCCGGGGCGAAT

>Seq332 [organism=Homo sapiens] Control ApoE ε2ε4

TACGCCGGCGGCGCGGAATGGAGGACGTGTGCGGCCGCCTGGTGCAGTACCGCGGCGAGGTGCAGGCCATGCTCGGCCAGAGCACCGAGGAGCTGCGGGTGCGCCTCGCCTCCCACCTGCGCAAGCTGCGTAAGCGGCTCCTCCGCGATGCCGATGACCTGCAGAAGCGCCTGGCAGTGTACCAGGCCGGGGCGAAT

>Seq333 [organism=Homo sapiens] Control ApoE ε2ε4

TCGACCGGCGGCGCGGAATGGAGGACGTGTGCGGCCGCCTGGTGCAGTACCGCGGCGAGGTGCAGGCCATGCTCGGCCAGAGCACCGAGGAGCTGCGGGTGCGCCTCGCCTCCCACCTGCGCAAGCTGCGTAAGCGGCTCCTCCGCGATGCCGATGACCTGCAGAAGCGCCTGGCAGTGTACCAGGCCGGGGCGAAT

>Seq334 [organism=Homo sapiens] Control ApoE ε2ε4

TGCTGCGGCGGCGCGGAATGGAGGACGTGTGCGGCCGCCTGGTGCAGTACCGCGGCGAGGTGCAGGCCATGCTCGGCCAGAGCACCGAGGAGCTGCGGGTGCGCCTCGCCTCCCACCTGCGCAAGCTGCGTAAGCGGCTCCTCCGCGATGCCGATGACCTGCAGAAGCGCCTGGCAGTGTACCAGGCCGGGGCGAAT

>Seq335 [organism=Homo sapiens] Control ApoE ε2ε4

GACCGCGGCGGCGCGGAATGGAGGACGTGTGCGGCCGCCTGGTGCAGTACCGCGGCGAGGTGCAGGCCATGCTCGGCCAGAGCACCGAGGAGCTGCGGGTGCGCCTCGCCTCCCACCTGCGCAAGCTGCGTAAGCGGCTCCTCCGCGATGCCGATGACCTGCAGAAGCGCCTGGCAGTGTACCAGGCCGGGGCGAAT

>Seq336 [organism=Homo sapiens] Control ApoE ε2ε4

TAGCGCGGCGGCGCGGAATGGAGGACGTGTGCGGCCGCCTGGTGCAGTACCGCGGCGAGGTGCAGGCCATGCTCGGCCAGAGCACCGAGGAGCTGCGGGTGCGCCTCGCCTCCCACCTGCGCAAGCTGCGTAAGCGGCTCCTCCGCGATGCCGATGACCTGCAGAAGCGCCTGGCAGTGTACCAGGCCGGGGCGAAT

>Seq337 [organism=Homo sapiens] Control ApoE ε2ε4

ATCCGCGGCGGCGCGGAATGGAGGACGTGTGCGGCCGCCTGGTGCAGTACCGCGGCGAGGTGCAGGCCATGCTCGGCCAGAGCACCGAGGAGCTGCGGGTGCGCCTCGCCTCCCACCTGCGCAAGCTGCGTAAGCGGCTCCTCCGCGATGCCGATGACCTGCAGAAGCGCCTGGCAGTGTACCAGGCCGGGGCGAAT

>Seq338 [organism=Homo sapiens] Control ApoE ε2ε4

CGGCGCGGCGGCGCGGAATGGAGGACGTGTGCGGCCGCCTGGTGCAGTACCGCGGCGAGGTGCAGGCCATGCTCGGCCAGAGCACCGAGGAGCTGCGGGTGCGCCTCGCCTCCCACCTGCGCAAGCTGCGTAAGCGGCTCCTCCGCGATGCCGATGACCTGCAGAAGCGCCTGGCAGTGTACCAGGCCGGGGCGAAT

>Seq339 [organism=Homo sapiens] Control ApoE ε2ε4

TGGAGCGGCGGCGCGGAATGGAGGACGTGTGCGGCCGCCTGGTGCAGTACCGCGGCGAGGTGCAGGCCATGCTCGGCCAGAGCACCGAGGAGCTGCGGGTGCGCCTCGCCTCCCACCTGCGCAAGCTGCGTAAGCGGCTCCTCCGCGATGCCGATGACCTGCAGAAGCGCCTGGCAGTGTACCAGGCCGGGGCGAAT

>Seq340 [organism=Homo sapiens] Control ApoE ε2ε4

GACTGCGGCGGCGCGGAATGGAGGACGTGTGCGGCCGCCTGGTGCAGTACCGCGGCGAGGTGCAGGCCATGCTCGGCCAGAGCACCGAGGAGCTGCGGGTGCGCCTCGCCTCCCACCTGCGCAAGCTGCGTAAGCGGCTCCTCCGCGATGCCGATGACCTGCAGAAGCGCCTGGCAGTGTACCAGGCCGGGGCGAAT

>Seq341 [organism=Homo sapiens] Control ApoE ε2ε4

TGAGCCGGCGGCGCGGAATGGAGGACGTGTGCGGCCGCCTGGTGCAGTACCGCGGCGAGGTGCAGGCCATGCTCGGCCAGAGCACCGAGGAGCTGCGGGTGCGCCTCGCCTCCCACCTGCGCAAGCTGCGTAAGCGGCTCCTCCGCGATGCCGATGACCTGCAGAAGCGCCTGGCAGTGTACCAGGCCGGGGCGAAT

>Seq342 [organism=Homo sapiens] Control ApoE ε3ε4

TACAGCGGCGGCGCGGAATGGAGGACGTGTGCGGCCGCCTGGTGCAGTACCGCGGCGAGGTGCAGGCCATGCTCGGCCAGAGCACCGAGGAGCTGCGGGTGCGCCTCGCCTCCCACCTGCGCAAGCTGCGTAAGCGGCTCCTCCGCGATGCCGATGACCTGCAGAAGCGCCTGGCAGTGTACCAGGCCGGGGCGAAT

>Seq343 [organism=Homo sapiens] Control ApoE ε3ε4

AGCTGCGGCGGCGCGGAATGGAGGACGTGTGCGGCCGCCTGGTGCAGTACCGCGGCGAGGTGCAGGCCATGCTCGGCCAGAGCACCGAGGAGCTGCGGGTGCGCCTCGCCTCCCACCTGCGCAAGCTGCGTAAGCGGCTCCTCCGCGATGCCGATGACCTGCAGAAGCGCCTGGCAGTGTACCAGGCCGGGGCGAAT

>Seq344 [organism=Homo sapiens] Control ApoE ε3ε4

CGACTCGGCGGCGCGGAATGGAGGACGTGTGCGGCCGCCTGGTGCAGTACCGCGGCGAGGTGCAGGCCATGCTCGGCCAGAGCACCGAGGAGCTGCGGGTGCGCCTCGCCTCCCACCTGCGCAAGCTGCGTAAGCGGCTCCTCCGCGATGCCGATGACCTGCAGAAGCGCCTGGCAGTGTACCAGGCCGGGGCGAAT

>Seq345 [organism=Homo sapiens] Control ApoE ε3ε4

TGCGGCGGCGGCGCGGAATGGAGGACGTGTGCGGCCGCCTGGTGCAGTACCGCGGCGAGGTGCAGGCCATGCTCGGCCAGAGCACCGAGGAGCTGCGGGTGCGCCTCGCCTCCCACCTGCGCAAGCTGCGTAAGCGGCTCCTCCGCGATGCCGATGACCTGCAGAAGCGCCTGGCAGTGTACCAGGCCGGGGCGAAT

>Seq346 [organism=Homo sapiens] Control ApoE ε3ε4

ACGGCCGGCGGCGCGGAATGGAGGACGTGTGCGGCCGCCTGGTGCAGTACCGCGGCGAGGTGCAGGCCATGCTCGGCCAGAGCACCGAGGAGCTGCGGGTGCGCCTCGCCTCCCACCTGCGCAAGCTGCGTAAGCGGCTCCTCCGCGATGCCGATGACCTGCAGAAGCGCCTGGCAGTGTACCAGGCCGGGGCGAAT

>Seq347 [organism=Homo sapiens] Control ApoE ε3ε4

TACCGCGGCGGCGCGGAATGGAGGACGTGTGCGGCCGCCTGGTGCAGTACCGCGGCGAGGTGCAGGCCATGCTCGGCCAGAGCACCGAGGAGCTGCGGGTGCGCCTCGCCTCCCACCTGCGCAAGCTGCGTAAGCGGCTCCTCCGCGATGCCGATGACCTGCAGAAGCGCCTGGCAGTGTACCAGGCCGGGGCGAAT

>Seq348 [organism=Homo sapiens] Control ApoE ε3ε4

GACTCCGGCGGCGCGGAATGGAGGACGTGTGCGGCCGCCTGGTGCAGTACCGCGGCGAGGTGCAGGCCATGCTCGGCCAGAGCACCGAGGAGCTGCGGGTGCGCCTCGCCTCCCACCTGCGCAAGCTGCGTAAGCGGCTCCTCCGCGATGCCGATGACCTGCAGAAGCGCCTGGCAGTGTACCAGGCCGGGGCGAAT

>Seq349 [organism=Homo sapiens] Control ApoE ε3ε4

TGCAGCGGCGGCGCGGAATGGAGGACGTGTGCGGCCGCCTGGTGCAGTACCGCGGCGAGGTGCAGGCCATGCTCGGCCAGAGCACCGAGGAGCTGCGGGTGCGCCTCGCCTCCCACCTGCGCAAGCTGCGTAAGCGGCTCCTCCGCGATGCCGATGACCTGCAGAAGCGCCTGGCAGTGTACCAGGCCGGGGCGAAT

>Seq350 [organism=Homo sapiens] Control ApoE ε3ε4

TACAGCGGCGGCGCGGAATGGAGGACGTGTGCGGCCGCCTGGTGCAGTACCGCGGCGAGGTGCAGGCCATGCTCGGCCAGAGCACCGAGGAGCTGCGGGTGCGCCTCGCCTCCCACCTGCGCAAGCTGCGTAAGCGGCTCCTCCGCGATGCCGATGACCTGCAGAAGCGCCTGGCAGTGTACCAGGCCGGGGCGAAT

>Seq351 [organism=Homo sapiens] Control ApoE ε3ε4

CGATGCGGCGGCGCGGAATGGAGGACGTGTGCGGCCGCCTGGTGCAGTACCGCGGCGAGGTGCAGGCCATGCTCGGCCAGAGCACCGAGGAGCTGCGGGTGCGCCTCGCCTCCCACCTGCGCAAGCTGCGTAAGCGGCTCCTCCGCGATGCCGATGACCTGCAGAAGCGCCTGGCAGTGTACCAGGCCGGGGCGAAT

>Seq352 [organism=Homo sapiens] Control ApoE ε3ε4

TGCACCGGCGGCGCGGAATGGAGGACGTGTGCGGCCGCCTGGTGCAGTACCGCGGCGAGGTGCAGGCCATGCTCGGCCAGAGCACCGAGGAGCTGCGGGTGCGCCTCGCCTCCCACCTGCGCAAGCTGCGTAAGCGGCTCCTCCGCGATGCCGATGACCTGCAGAAGCGCCTGGCAGTGTACCAGGCCGGGGCGAAT

>Seq353 [organism=Homo sapiens] Control ApoE ε3ε4

AGCGGCGGCGGCGCGGAATGGAGGACGTGTGCGGCCGCCTGGTGCAGTACCGCGGCGAGGTGCAGGCCATGCTCGGCCAGAGCACCGAGGAGCTGCGGGTGCGCCTCGCCTCCCACCTGCGCAAGCTGCGTAAGCGGCTCCTCCGCGATGCCGATGACCTGCAGAAGCGCCTGGCAGTGTACCAGGCCGGGGCGAAT

>Seq354 [organism=Homo sapiens] Control ApoE ε3ε4

TACAGCGGCGGCGCGGAATGGAGGACGTGTGCGGCCGCCTGGTGCAGTACCGCGGCGAGGTGCAGGCCATGCTCGGCCAGAGCACCGAGGAGCTGCGGGTGCGCCTCGCCTCCCACCTGCGCAAGCTGCGTAAGCGGCTCCTCCGCGATGCCGATGACCTGCAGAAGCGCCTGGCAGTGTACCAGGCCGGGGCGAAT

>Seq355 [organism=Homo sapiens] Control ApoE ε3ε4

TACGGCGGCGGCGCGGAATGGAGGACGTGTGCGGCCGCCTGGTGCAGTACCGCGGCGAGGTGCAGGCCATGCTCGGCCAGAGCACCGAGGAGCTGCGGGTGCGCCTCGCCTCCCACCTGCGCAAGCTGCGTAAGCGGCTCCTCCGCGATGCCGATGACCTGCAGAAGCGCCTGGCAGTGTACCAGGCCGGGGCGAAT

>Seq356 [organism=Homo sapiens] Control ApoE ε3ε4

TGCAGCGGCGGCGCGGAATGGAGGACGTGTGCGGCCGCCTGGTGCAGTACCGCGGCGAGGTGCAGGCCATGCTCGGCCAGAGCACCGAGGAGCTGCGGGTGCGCCTCGCCTCCCACCTGCGCAAGCTGCGTAAGCGGCTCCTCCGCGATGCCGATGACCTGCAGAAGCGCCTGGCAGTGTACCAGGCCGGGGCGAAT

>Seq357 [organism=Homo sapiens] Control ApoE ε3ε4

AGTCGCGGCGGCGCGGAATGGAGGACGTGTGCGGCCGCCTGGTGCAGTACCGCGGCGAGGTGCAGGCCATGCTCGGCCAGAGCACCGAGGAGCTGCGGGTGCGCCTCGCCTCCCACCTGCGCAAGCTGCGTAAGCGGCTCCTCCGCGATGCCGATGACCTGCAGAAGCGCCTGGCAGTGTACCAGGCCGGGGCGAAT

>Seq358 [organism=Homo sapiens] Control ApoE ε3ε4

GTCAGCGGCGGCGCGGAATGGAGGACGTGTGCGGCCGCCTGGTGCAGTACCGCGGCGAGGTGCAGGCCATGCTCGGCCAGAGCACCGAGGAGCTGCGGGTGCGCCTCGCCTCCCACCTGCGCAAGCTGCGTAAGCGGCTCCTCCGCGATGCCGATGACCTGCAGAAGCGCCTGGCAGTGTACCAGGCCGGGGCGAAT

>Seq359 [organism=Homo sapiens] Control ApoE ε3ε4

CGTCGCGGCGGCGCGGAATGGAGGACGTGTGCGGCCGCCTGGTGCAGTACCGCGGCGAGGTGCAGGCCATGCTCGGCCAGAGCACCGAGGAGCTGCGGGTGCGCCTCGCCTCCCACCTGCGCAAGCTGCGTAAGCGGCTCCTCCGCGATGCCGATGACCTGCAGAAGCGCCTGGCAGTGTACCAGGCCGGGGCGAAT

>Seq360 [organism=Homo sapiens] Control ApoE ε3ε4

TGCCGCGGCGGCGCGGAATGGAGGACGTGTGCGGCCGCCTGGTGCAGTACCGCGGCGAGGTGCAGGCCATGCTCGGCCAGAGCACCGAGGAGCTGCGGGTGCGCCTCGCCTCCCACCTGCGCAAGCTGCGTAAGCGGCTCCTCCGCGATGCCGATGACCTGCAGAAGCGCCTGGCAGTGTACCAGGCCGGGGCGAAT

>Seq361 [organism=Homo sapiens] Control ApoE ε3ε4

AGCGGCGGCGGCGCGGAATGGAGGACGTGTGCGGCCGCCTGGTGCAGTACCGCGGCGAGGTGCAGGCCATGCTCGGCCAGAGCACCGAGGAGCTGCGGGTGCGCCTCGCCTCCCACCTGCGCAAGCTGCGTAAGCGGCTCCTCCGCGATGCCGATGACCTGCAGAAGCGCCTGGCAGTGTACCAGGCCGGGGCGAAT

>Seq362 [organism=Homo sapiens] Control ApoE ε3ε4

TACAGCGGCGGCGCGGAATGGAGGACGTGTGCGGCCGCCTGGTGCAGTACCGCGGCGAGGTGCAGGCCATGCTCGGCCAGAGCACCGAGGAGCTGCGGGTGCGCCTCGCCTCCCACCTGCGCAAGCTGCGTAAGCGGCTCCTCCGCGATGCCGATGACCTGCAGAAGCGCCTGGCAGTGTACCAGGCCGGGGCGAAT

>Seq363 [organism=Homo sapiens] Control ApoE ε3ε4

TGCGGCGGCGGCGCGGAATGGAGGACGTGTGCGGCCGCCTGGTGCAGTACCGCGGCGAGGTGCAGGCCATGCTCGGCCAGAGCACCGAGGAGCTGCGGGTGCGCCTCGCCTCCCACCTGCGCAAGCTGCGTAAGCGGCTCCTCCGCGATGCCGATGACCTGCAGAAGCGCCTGGCAGTGTACCAGGCCGGGGCGAAT

>Seq364 [organism=Homo sapiens] Control ApoE ε3ε4

AGTCGCGGCGGCGCGGAATGGAGGACGTGTGCGGCCGCCTGGTGCAGTACCGCGGCGAGGTGCAGGCCATGCTCGGCCAGAGCACCGAGGAGCTGCGGGTGCGCCTCGCCTCCCACCTGCGCAAGCTGCGTAAGCGGCTCCTCCGCGATGCCGATGACCTGCAGAAGCGCCTGGCAGTGTACCAGGCCGGGGCGAAT

>Seq365 [organism=Homo sapiens] Control ApoE ε3ε4

TGCCGCGGCGGCGCGGAATGGAGGACGTGTGCGGCCGCCTGGTGCAGTACCGCGGCGAGGTGCAGGCCATGCTCGGCCAGAGCACCGAGGAGCTGCGGGTGCGCCTCGCCTCCCACCTGCGCAAGCTGCGTAAGCGGCTCCTCCGCGATGCCGATGACCTGCAGAAGCGCCTGGCAGTGTACCAGGCCGGGGCGAAT

>Seq366 [organism=Homo sapiens] Control ApoE ε3ε4

AGCGCCGGCGGCGCGGAATGGAGGACGTGTGCGGCCGCCTGGTGCAGTACCGCGGCGAGGTGCAGGCCATGCTCGGCCAGAGCACCGAGGAGCTGCGGGTGCGCCTCGCCTCCCACCTGCGCAAGCTGCGTAAGCGGCTCCTCCGCGATGCCGATGACCTGCAGAAGCGCCTGGCAGTGTACCAGGCCGGGGCGAAT

>Seq367 [organism=Homo sapiens] Control ApoE ε2ε4

TACCGCGGCGGCGCGGAATGGAGGACGTGTGCGGCCGCCTGGTGCAGTACCGCGGCGAGGTGCAGGCCATGCTCGGCCAGAGCACCGAGGAGCTGCGGGTGCGCCTCGCCTCCCACCTGCGCAAGCTGCGTAAGCGGCTCCTCCGCGATGCCGATGACCTGCAGAAGCGCCTGGCAGTGTACCAGGCCGGGGCGAAT

>Seq368 [organism=Homo sapiens] Control ApoE ε3ε4

GACCGCGGCGGCGCGGAATGGAGGACGTGTGCGGCCGCCTGGTGCAGTACCGCGGCGAGGTGCAGGCCATGCTCGGCCAGAGCACCGAGGAGCTGCGGGTGCGCCTCGCCTCCCACCTGCGCAAGCTGCGTAAGCGGCTCCTCCGCGATGCCGATGACCTGCAGAAGCGCCTGGCAGTGTACCAGGCCGGGGCGAAT

>Seq369 [organism=Homo sapiens] Control ApoE ε3ε4

TGCAGCGGCGGCGCGGAATGGAGGACGTGTGCGGCCGCCTGGTGCAGTACCGCGGCGAGGTGCAGGCCATGCTCGGCCAGAGCACCGAGGAGCTGCGGGTGCGCCTCGCCTCCCACCTGCGCAAGCTGCGTAAGCGGCTCCTCCGCGATGCCGATGACCTGCAGAAGCGCCTGGCAGTGTACCAGGCCGGGGCGAAT

>Seq370 [organism=Homo sapiens] Control ApoE ε3ε4

TACGGCGGCGGCGCGGAATGGAGGACGTGTGCGGCCGCCTGGTGCAGTACCGCGGCGAGGTGCAGGCCATGCTCGGCCAGAGCACCGAGGAGCTGCGGGTGCGCCTCGCCTCCCACCTGCGCAAGCTGCGTAAGCGGCTCCTCCGCGATGCCGATGACCTGCAGAAGCGCCTGGCAGTGTACCAGGCCGGGGCGAAT

>Seq371 [organism=Homo sapiens] Control ApoE ε3ε4

GACTGCGGCGGCGCGGAATGGAGGACGTGTGCGGCCGCCTGGTGCAGTACCGCGGCGAGGTGCAGGCCATGCTCGGCCAGAGCACCGAGGAGCTGCGGGTGCGCCTCGCCTCCCACCTGCGCAAGCTGCGTAAGCGGCTCCTCCGCGATGCCGATGACCTGCAGAAGCGCCTGGCAGTGTACCAGGCCGGGGCGAAT

>Seq372 [organism=Homo sapiens] Control ApoE ε3ε4

AGCTGCGGCGGCGCGGAATGGAGGACGTGTGCGGCCGCCTGGTGCAGTACCGCGGCGAGGTGCAGGCCATGCTCGGCCAGAGCACCGAGGAGCTGCGGGTGCGCCTCGCCTCCCACCTGCGCAAGCTGCGTAAGCGGCTCCTCCGCGATGCCGATGACCTGCAGAAGCGCCTGGCAGTGTACCAGGCCGGGGCGAAT

>Seq373 [organism=Homo sapiens] Control ApoE ε3ε4

GACAGCGGCGGCGCGGAATGGAGGACGTGTGCGGCCGCCTGGTGCAGTACCGCGGCGAGGTGCAGGCCATGCTCGGCCAGAGCACCGAGGAGCTGCGGGTGCGCCTCGCCTCCCACCTGCGCAAGCTGCGTAAGCGGCTCCTCCGCGATGCCGATGACCTGCAGAAGCGCCTGGCAGTGTACCAGGCCGGGGCGAAT

>Seq374 [organism=Homo sapiens] Control ApoE ε3ε4

GCGGGTGGGCGGCGCGGAATGGAGGACGTGTGCGGCCGCCTGGTGCAGTACCGCGGCGAGGTGCAGGCCATGCTCGGCCAGAGCACCGAGGAGCTGCGGGTGCGCCTCGCCTCCCACCTGCGCAAGCTGCGTAAGCGGCTCCTCCGCGATGCCGATGACCTGCAGAAGCGCCTGGCAGTGTACCAGGCCGGGGCGAAT

>Seq375 [organism=Homo sapiens] Control ApoE ε3ε4

CTCCTCCGGGCGCGGAATGGAGGACGTGTGCGGCCGCCTGGTGCAGTACCGCGGCGAGGTGCAGGCCATGCTCGGCCAGAGCACCGAGGAGCTGCGGGTGCGCCTCGCCTCCCACCTGCGCAAGCTGCGTAAGCGGCTCCTCCGCGATGCCGATGACCTGCAGAAGCGCCTGGCAGTGTACCAGGCCGGGGCGAAT

>Seq376 [organism=Homo sapiens] Control ApoE ε3ε4

CGGCCAGAGCTGGAGGACGTGTGCGGCCGCCTGGTGCAGTACCGCGGCGAGGTGCAGGCCATGCTCGGCCAGAGCACCGAGGAGCTGCGGGTGCGCCTCGCCTCCCACCTGCGCAAGCTGCGTAAGCGGCTCCTCCGCGATGCCGATGACCTGCAGAAGCGCCTGGCAGTGTACCAGGCCGGGGCGAAT

>Seq377 [organism=Homo sapiens] Control ApoE ε3ε4

AGCGGCTCGAGGACGTGTGCGGCCGCCTGGTGCAGTACCGCGGCGAGGTGCAGGCCATGCTCGGCCAGAGCACCGAGGAGCTGCGGGTGCGCCTCGCCTCCCACCTGCGCAAGCTGCGTAAGCGGCTCCTCCGCGATGCCGATGACCTGCAGAAGCGCCTGGCAGTGTACCAGGCCGGGGCGAAT

>Seq378 [organism=Homo sapiens] Control ApoE ε3ε4

AGCGGCTCGAGGACGTGTGCGGCCGCCTGGTGCAGTACCGCGGCGAGGTGCAGGCCATGCTCGGCCAGAGCACCGAGGAGCTGCGGGTGCGCCTCGCCTCCCACCTGCGCAAGCTGCGTAAGCGGCTCCTCCGCGATGCCGATGACCTGCAGAAGCGCCTGGCAGTGTACCAGGCCGGGGCGAAT

>Seq379 [organism=Homo sapiens] Control ApoE ε3ε4

AGCTGCGGGTGGACGTGTGCGGCCGCCTGGTGCAGTACCGCGGCGAGGTGCAGGCCATGCTCGGCCAGAGCACCGAGGAGCTGCGGGTGCGCCTCGCCTCCCACCTGCGCAAGCTGCGTAAGCGGCTCCTCCGCGATGCCGATGACCTGCAGAAGCGCCTGGCAGTGTACCAGGCCGGGGCGAAT

>Seq379 [organism=Homo sapiens] Control ApoE ε3ε4

AGCTGCGGGTGGACGTGTGCGGCCGCCTGGTGCAGTACCGCGGCGAGGTGCAGGCCATGCTCGGCCAGAGCACCGAGGAGCTGCGGGTGCGCCTCGCCTCCCACCTGCGCAAGCTGCGTAAGCGGCTCCTCCGCGATGCCGATGACCTGCAGAAGCGCCTGGCAGTGTACCAGGCCGGGGCGAAT

>Seq381 [organism=Homo sapiens] Control ApoE ε3ε4

AGTCGCGGCGGCGCGGAATGGAGGACGTGTGCGGCCGCCTGGTGCAGTACCGCGGCGAGGTGCAGGCCATGCTCGGCCAGAGCACCGAGGAGCTGCGGGTGCGCCTCGCCTCCCACCTGCGCAAGCTGCGTAAGCGGCTCCTCCGCGATGCCGATGACCTGCAGAAGCGCCTGGCAGTGTACCAGGCCGGGGCGAAT

>Seq382 [organism=Homo sapiens] Control ApoE ε3ε4

TGCCGCGGCGGCGCGGAATGGAGGACGTGTGCGGCCGCCTGGTGCAGTACCGCGGCGAGGTGCAGGCCATGCTCGGCCAGAGCACCGAGGAGCTGCGGGTGCGCCTCGCCTCCCACCTGCGCAAGCTGCGTAAGCGGCTCCTCCGCGATGCCGATGACCTGCAGAAGCGCCTGGCAGTGTACCAGGCCGGGGCGAAT

>Seq383 [organism=Homo sapiens] Control ApoE ε3ε4

TACAGCGGCGGCGCGGAATGGAGGACGTGTGCGGCCGCCTGGTGCAGTACCGCGGCGAGGTGCAGGCCATGCTCGGCCAGAGCACCGAGGAGCTGCGGGTGCGCCTCGCCTCCCACCTGCGCAAGCTGCGTAAGCGGCTCCTCCGCGATGCCGATGACCTGCAGAAGCGCCTGGCAGTGTACCAGGCCGGGGCGAAT

>Seq384 [organism=Homo sapiens] Control ApoE ε3ε4

GACTGCGGCGGCGCGGAATGGAGGACGTGTGCGGCCGCCTGGTGCAGTACCGCGGCGAGGTGCAGGCCATGCTCGGCCAGAGCACCGAGGAGCTGCGGGTGCGCCTCGCCTCCCACCTGCGCAAGCTGCGTAAGCGGCTCCTCCGCGATGCCGATGACCTGCAGAAGCGCCTGGCAGTGTACCAGGCCGGGGCGAAT

>Seq385 [organism=Homo sapiens] Control ApoE ε3ε4

AGTCGCGGCGGCGCGGAATGGAGGACGTGTGCGGCCGCCTGGTGCAGTACCGCGGCGAGGTGCAGGCCATGCTCGGCCAGAGCACCGAGGAGCTGCGGGTGCGCCTCGCCTCCCACCTGCGCAAGCTGCGTAAGCGGCTCCTCCGCGATGCCGATGACCTGCAGAAGCGCCTGGCAGTGTACCAGGCCGGGGCGAAT

>Seq386 [organism=Homo sapiens] Control ApoE ε3ε4

TACCGCGGCGGCGCGGAATGGAGGACGTGTGCGGCCGCCTGGTGCAGTACCGCGGCGAGGTGCAGGCCATGCTCGGCCAGAGCACCGAGGAGCTGCGGGTGCGCCTCGCCTCCCACCTGCGCAAGCTGCGTAAGCGGCTCCTCCGCGATGCCGATGACCTGCAGAAGCGCCTGGCAGTGTACCAGGCCGGGGCGAAT

>Seq387 [organism=Homo sapiens] Control ApoE ε3ε4

GACCGCGGCGGCGCGGAATGGAGGACGTGTGCGGCCGCCTGGTGCAGTACCGCGGCGAGGTGCAGGCCATGCTCGGCCAGAGCACCGAGGAGCTGCGGGTGCGCCTCGCCTCCCACCTGCGCAAGCTGCGTAAGCGGCTCCTCCGCGATGCCGATGACCTGCAGAAGCGCCTGGCAGTGTACCAGGCCGGGGCGAAT

>Seq388 [organism=Homo sapiens] Control ApoE ε3ε4

TACGGCGGCGGCGCGGAATGGAGGACGTGTGCGGCCGCCTGGTGCAGTACCGCGGCGAGGTGCAGGCCATGCTCGGCCAGAGCACCGAGGAGCTGCGGGTGCGCCTCGCCTCCCACCTGCGCAAGCTGCGTAAGCGGCTCCTCCGCGATGCCGATGACCTGCAGAAGCGCCTGGCAGTGTACCAGGCCGGGGCGAAT

>Seq389 [organism=Homo sapiens] Control ApoE ε3ε4

GACAGCGGCGGCGCGGAATGGAGGACGTGTGCGGCCGCCTGGTGCAGTACCGCGGCGAGGTGCAGGCCATGCTCGGCCAGAGCACCGAGGAGCTGCGGGTGCGCCTCGCCTCCCACCTGCGCAAGCTGCGTAAGCGGCTCCTCCGCGATGCCGATGACCTGCAGAAGCGCCTGGCAGTGTACCAGGCCGGGGCGAAT

>Seq390 [organism=Homo sapiens] Control ApoE ε3ε4

GACTGCGGCGCGGGCAGACCTAGCGCCCAGCGGCTGGGCGCGGAATGGAGGACGTGTGCGGCCGCCTGGTGCAGTACCGCGGCGAGGTGCAGGCCATGCTCGGCCAGAGCACCGAGGAGCTGCGGGTGCGCCTCGCCTCCCACCTGCGCAAGCTGCGTAAGCGGCTCCTCCGCGATGCCGATGACCTGCAGAAGCGCCTGGCAGTGTACCAGGCCGGGGCGAATGCGAATA

>Seq391 [organism=Homo sapiens] Control ApoE ε3ε4

TACAGCGGCGCGGGCAGACCTAGCGCCCAGCGGCTGGGCGCGGAATGGAGGACGTGTGCGGCCGCCTGGTGCAGTACCGCGGCGAGGTGCAGGCCATGCTCGGCCAGAGCACCGAGGAGCTGCGGGTGCGCCTCGCCTCCCACCTGCGCAAGCTGCGTAAGCGGCTCCTCCGCGATGCCGATGACCTGCAGAAGCGCCTGGCAGTGTACCAGGCCGGGGCGAATGCGAATA

>Seq392 [organism=Homo sapiens] Control ApoE ε4ε4

GACTGCGGCGGGCGCGGAATGGAGGACGTGCGCGGCCGCCTGGTGCAGTACCGCGGCGAGGTGCAGGCCATGCTCGGCCAGAGCACCGAGGAGCTGCGGGTGCGCCTCGCCTCCCACCTGCGCAAGCTGCGTAAGCGGCTCCTCCGCGATGCCGATGACCTGCAGAAGCGCCTGGCAGTGTACCAGGCCGGGGCGAAT

>Seq393 [organism=Homo sapiens] Control ApoE ε4ε4

TACGGCGGCGGGCGCGGAATGGAGGACGTGCGCGGCCGCCTGGTGCAGTACCGCGGCGAGGTGCAGGCCATGCTCGGCCAGAGCACCGAGGAGCTGCGGGTGCGCCTCGCCTCCCACCTGCGCAAGCTGCGTAAGCGGCTCCTCCGCGATGCCGATGACCTGCAGAAGCGCCTGGCAGTGTACCAGGCCGGGGCGAAT

>Seq394 [organism=Homo sapiens] Control ApoE ε4ε4

GACCGCGGCGGCGCGGAATGGAGGACGTGCGCGGCCGCCTGGTGCAGTACCGCGGCGAGGTGCAGGCCATGCTCGGCCAGAGCACCGAGGAGCTGCGGGTGCGCCTCGCCTCCCACCTGCGCAAGCTGCGTAAGCGGCTCCTCCGCGATGCCGATGACCTGCAGAAGCGCCTGGCAGTGTACCAGGCCGGGGCGAAT

>Seq395 [organism=Homo sapiens] Control ApoE ε4ε4

TGCGGCGGGGCGCGGAATGGAGGACGTGCGCGGCCGCCTGGTGCAGTACCGCGGCGAGGTGCAGGCCATGCTCGGCCAGAGCACCGAGGAGCTGCGGGTGCGCCTCGCCTCCCACCTGCGCAAGCTGCGTAAGCGGCTCCTCCGCGATGCCGATGACCTGCAGAAGCGCCTGGCAGTGTACCAGGCCGGGGCGAAT

>Seq396 [organism=Homo sapiens] Control ApoE ε4ε4

TACCGCGGCGGGCGCGGAATGGAGGACGTGCGCGGCCGCCTGGTGCAGTACCGCGGCGAGGTGCAGGCCATGCTCGGCCAGAGCACCGAGGAGCTGCGGGTGCGCCTCGCCTCCCACCTGCGCAAGCTGCGTAAGCGGCTCCTCCGCGATGCCGATGACCTGCAGAAGCGCCTGGCAGTGTACCAGGCCGGGGCGAAT

>Seq397 [organism=Homo sapiens] Control ApoE ε4ε4

AGCGGCGGGGCGCGGAATGGAGGACGTGCGCGGCCGCCTGGTGCAGTACCGCGGCGAGGTGCAGGCCATGCTCGGCCAGAGCACCGAGGAGCTGCGGGTGCGCCTCGCCTCCCACCTGCGCAAGCTGCGTAAGCGGCTCCTCCGCGATGCCGATGACCTGCAGAAGCGCCTGGCAGTGTACCAGGCCGGGGCGAAT

>Seq398 [organism=Homo sapiens] Control ApoE ε4ε4

TACAGCGGCGGGCGCGGAATGGAGGACGTGCGCGGCCGCCTGGTGCAGTACCGCGGCGAGGTGCAGGCCATGCTCGGCCAGAGCACCGAGGAGCTGCGGGTGCGCCTCGCCTCCCACCTGCGCAAGCTGCGTAAGCGGCTCCTCCGCGATGCCGATGACCTGCAGAAGCGCCTGGCAGTGTACCAGGCCGGGGCGAAT

>Seq399 [organism=Homo sapiens] Control ApoE ε4ε4

GACTGCGGCGGGCGGCGCGGAATGGAGGACGTGCGCGGCCGCCTGGTGCAGTACCGCGGCGAGGTGCAGGCCATGCTCGGCCAGAGCACCGAGGAGCTGCGGGTGCGCCTCGCCTCCCACCTGCGCAAGCTGCGTAAGCGGCTCCTCCGCGATGCCGATGACCTGCAGAAGCGCCTGGCAGTGTACCAGGCCGGGGCGAAT

>Seq400 [organism=Homo sapiens] Control ApoE ε4ε4

AGCTGCGGCGGGCGCGGAATGGAGGACGTGCGCGGCCGCCTGGTGCAGTACCGCGGCGAGGTGCAGGCCATGCTCGGCCAGAGCACCGAGGAGCTGCGGGTGCGCCTCGCCTCCCACCTGCGCAAGCTGCGTAAGCGGCTCCTCCGCGATGCCGATGACCTGCAGAAGCGCCTGGCAGTGTACCAGGCCGGGGCGAAT
