# Supplementary figures and images for: The role of APOE gene polymorphisms in lung adenocarcinoma susceptibility and lipid profile
Source: Front Immunol. 2024 Dec 23;15:1522761. doi: 10.3389/fimmu.2024.1522761 (PMC11701022; doi:10.3389/fimmu.2024.1522761)

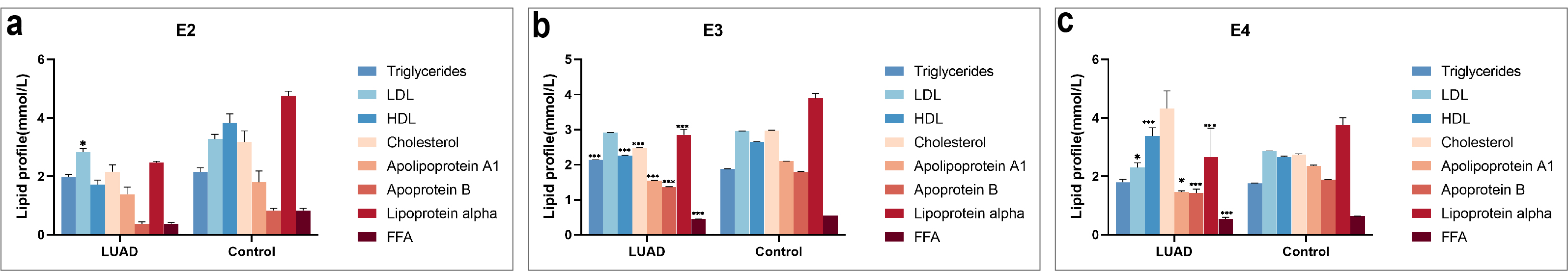

Supplement: Supplementary file 2 [file Image1.jpeg]

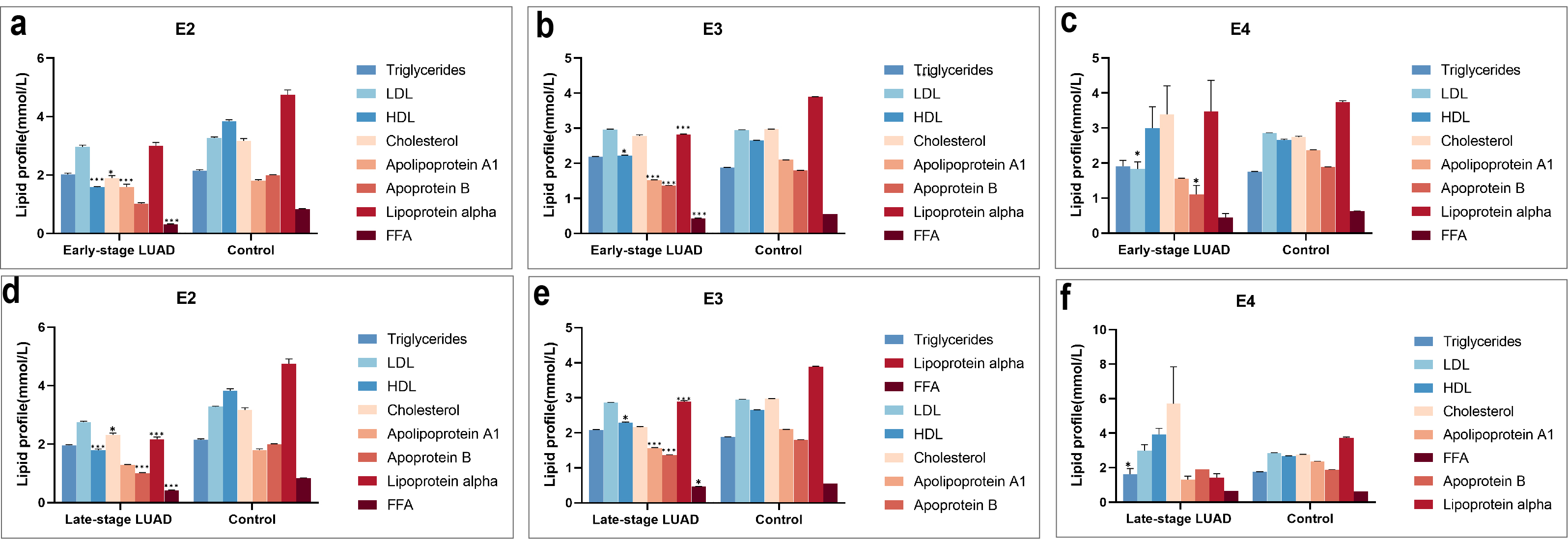

Supplement: Supplementary file 3 [file Image2.jpeg]

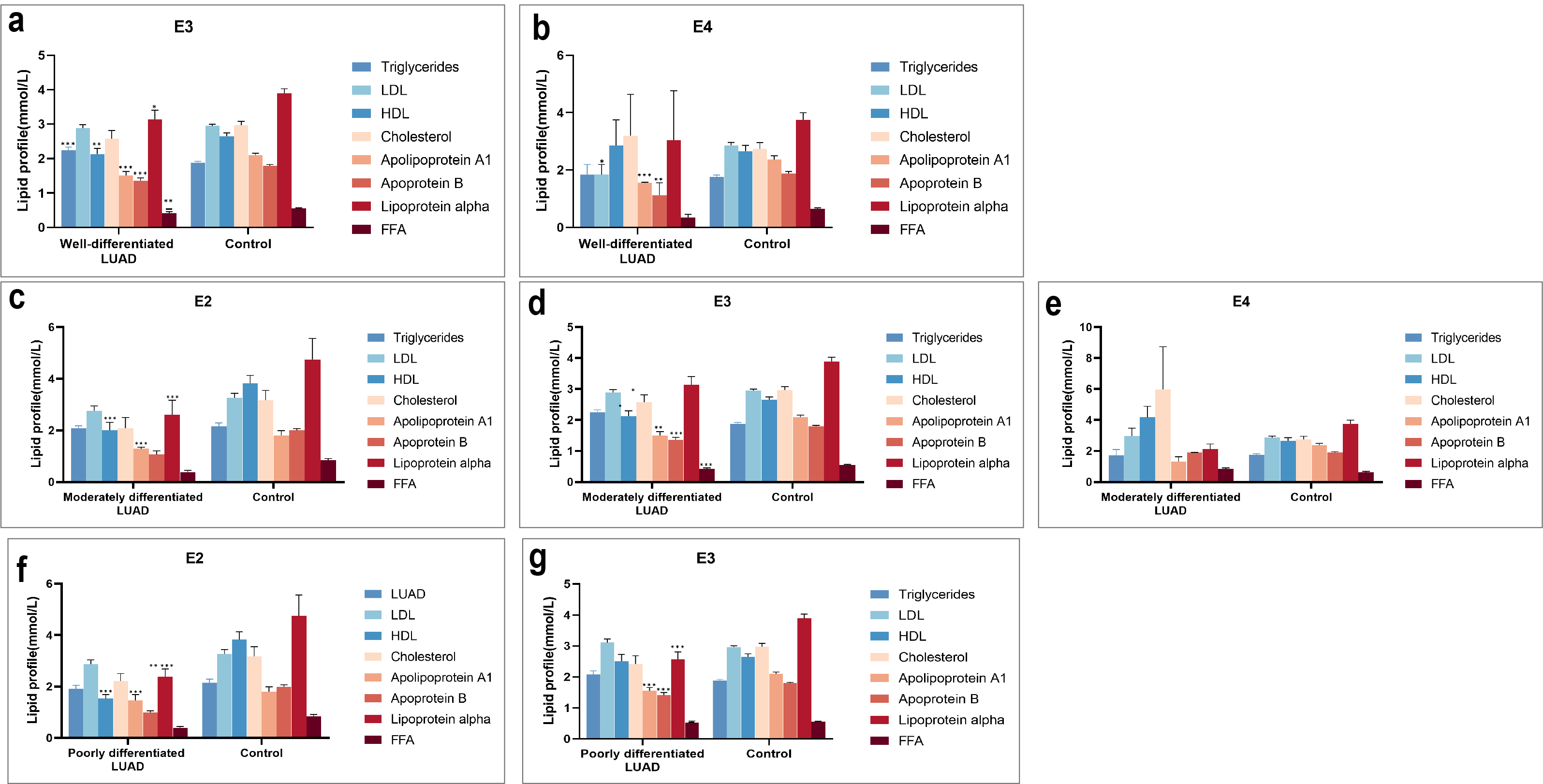

Supplement: Supplementary file 4 [file Image3.jpeg]
